# Cultured cells and wing disc size of silkworm can be controlled by the Hippo pathway

**DOI:** 10.1098/rsob.180029

**Published:** 2018-07-04

**Authors:** Zi Liang, Yahong Lu, Ying Qian, Liyuan Zhu, Sulan Kuang, Fei Chen, Yongjie Feng, Xiaolong Hu, Guangli Cao, Renyu Xue, Chengliang Gong

**Affiliations:** 1 School of Biology and Basic Medical Science, Agricultural Biotechnology and Ecological Research Institute, Soochow University, Suzhou, 215123, China; 2 National Engineering Laboratory for Modern Silk, Agricultural Biotechnology and Ecological Research Institute, Soochow University, Suzhou, 215123, China; 3 Agricultural Biotechnology Research Institute, Agricultural Biotechnology and Ecological Research Institute, Soochow University, Suzhou, 215123, China

**Keywords:** yorki, *Bombyx mori*, cell proliferation, wing disc size, differential gene expression

## Abstract

Hippo signalling represents a cell proliferation and organ-size control pathway. Yorki (Yki), a component of the Hippo pathway, induces the transcription of a number of targets that promote cell proliferation and survival. The functions of *Yki* have been characterized in *Drosophila* and mammals, while there are few reports on silkworm, *Bombyx mori*. In the present study, we found that *BmYki3* facilitates cell migration and cell division, and enlarges the cultured cell and wing disc size. Co-immunoprecipitation results indicated that BmYki3 may interact with thymosin, E3 ubiquitin-protein ligase, protein kinase ASK1, dedicator of cytokinesis protein 1, calcium-independent phospholipase A2 and beta-spectrin. RNA-seq results indicated that 4444 genes were upregulated and 10 291 genes were downregulated after *BmYki3* was overexpressed in the cultured cells. GO annotation indicated that the up/downregulated genes were enriched in 268/382 GO terms (*p* < 0.01); KEGG analysis showed that the up/downregulated genes were enriched in 49/101 pathways. These findings provided novel information to understand the functions of *BmYki3* in a cell proliferation and organ-size control pathway.

## 1. Background

The Hippo signalling pathway, first discovered in *Drosophila*, is a recently found growth control pathway [1]. It has been widely reported that the Hippo signalling pathway plays an important role in cell differentiation, pattern formation and organ size regulation. Furthermore, the Hippo kinase cascade has a close correlation with cancer initiation and progression, and its dysregulation may lead to tumorigenesis [2]. Recent studies have shown that the Hippo signalling pathway is evolutionary highly conserved and is shared by multicellular animals such as fruit flies, mice and other mammals.

The Hippo pathway controls *Drosophila* organ growth by regulating cell proliferation and apoptosis [3]. To date, over 30 components related to the Hippo pathway have been identified [4]. The Hippo pathway is defined by a kinase cascade whereby the serine-threonine-like kinase protein Hippo (Hpo), facilitated by the WW-domain-containing adaptor protein Salvador (Sav), phosphorylates and activates the NDR family kinase protein Warts (Wts). Mob-as-tumour-suppressor (Mats) is an essential cofactor for Wts. Wts, in turn, phosphorylates and inactivates the transcriptional coactivator Yorkie (Yki), leading to transcriptional downregulation of a series of target genes [5]. Inactivation of Hpo, Sav, Wts or Mats, or overexpression of *Yki*, results in massive tissue overgrowth characterized by excessive cell proliferation and diminished apoptosis [6]. Yki is phosphorylated by Wts in three serine (Ser) residues, of which the most important phosphorylation site is Yki Ser^168^ [7]. The 14-3-3 protein, a cytoskeletal protein, interacts with Yki Ser^168^ so that Yki is locked in the cytoplasm, unable to exert its transcriptional activation function [8–10].

The Hippo pathway has been extensively studied in both *Drosophila* and mammals. Both the structure and function of the Hippo pathway main core components are conserved between *Drosophila* and mammals, but there are some differences in some upstream components between *Drosophila* and mammals [11]. The silkworm *Bombyx mori*, an insect of economic importance, is a model species of the Lepidoptera and its whole genome sequence has been determined [12,13]. Many genes related to growth, development, metamorphosis, immunologic response and fibroin synthesis have been well studied. Control of organ size is very important in sericulture; the ability to regulate the size of the silk gland and ovary could improve the silk yield and oviposition number, respectively [14,15]. Hippo signalling represents a cell proliferation and organ-size control pathway; however, little is known about the genes related to the Hippo pathway.

In our previous study, *Hpo, Sav, Mats, Wts* and *Yki* were identified as genes related to the Hippo pathway in silkworm. Although the sequence identities of proteins from different species were not high, the conserved domains were prominent [16]. Yki has three isoforms in the silkworm. The results reported by Liu *et al*. [17] indicated that BmYki1 potentially facilitates posterior silk gland growth and metamorphosis in the silkworm. Furthermore, Zhang *et al*. [18] found that overexpression of Yorki^CA^ (Ser^97^ of the BmYki1 was mutated to Ala^97^) in the posterior silk gland significantly increased the weight of the posterior silk gland, cocoon, larval body and pupal body.

In the present study, we characterized the functions of a novel alternative splicing variant (*BmYki3*) of the *BmYki* gene and found that cultured cell and wing disc sizes can be controlled by regulating *BmYki3* expression. The comparative transcriptome showed that 4444 genes were upregulated and 10 291 genes were downregulated after *BmYki3* was overexpressed in the cultured cells. Functional analysis of differential gene expression showed that the expression levels of genes involved in the cell cycle, cell migration, apoptosis, innate immune response, steroid hormone biosynthesis, juvenile hormone biosynthetic process and MAPK signalling pathway were obviously changed by regulating *BmYki3* expression. These results will contribute to our understanding of the influence of the Hippo pathway on cell proliferation, organ size, resistance to pathogens and development in the silkworm.

## 2. Material and methods

### 2.1. RNA isolation, cDNA synthesis and cloning

Total RNA was isolated from silkworm (strain Dazhao) tissues using a total RNA Isolation Kit (TaKaRa, DaLian, China), followed by treatment with DNaseI to remove possible contamination from genomic DNA. cDNA was synthesized by PrimeScript™ Reverse Transcriptase (TaKaRa, DaLian, China), following the manufacturer's protocol. The cDNA was used as a template. The amplified products with gene-specific primers BmYki-1 and BmYki-2 were cloned into vector pMD19-T (TaKaRa, DaLian, China). *BmYki* cDNA was sequenced after the recombinant plasmids were identified.

### 2.2. qPCR

The relative expression level of genes was determined with qPCR. The housekeeping gene *actin A3* of *B. mori* was used as an internal control for normalization. A 20 µl volume containing 0.2 µg cDNA, 5 pmol of each primer and 10 µl of iTaq™ Universal SYBR Green Supermix (Bio-Rad, Berkeley, CA, Hercules, USA) was used for qPCR. qPCR was carried out using a real-time PCR system (Bio-Rad CFX96) according to the following programme: one cycle at 50°C for 2 min; one cycle at 95°C for 10 min; 40 cycles at 95°C for 15 s, 60°C for 1 min; one final cycle for dissociation at 95°C for 15 s, 60°C for 30 s and 95°C for 15 s. This experiment was repeated three times. The primers used in the present study were listed in the electronic supplementary material, table S1. The relative expression level of genes was estimated according to the 2^−ΔΔCt^ method [19].

### 2.3. *BmYki1* expression in *Escherichia coli* and antibody preparation

The *BmYki1* gene (GenBank accession no. KF904339.1) (1.3 kb) was cloned into the BamHI/XhoI sites of the expression vector pET-28a(+) (Novagen, Darmstadt, Germany) to generate the recombinant plasmid pET-28a (+)-*BmYki1*. The fusion protein was expressed in *E*. *coli* strain Transetta (DE3). The recombinant protein was used to immunize ICR mice (Soochow University, Suzhou, China) by subcutaneous injection. The prepared antibody was then identified by western blotting.

### 2.4. SDS-PAGE and western blotting

The bacteria that were transformed with pET-28a(+)-*BmYki1* were mixed with 2×SDS loading buffer (0.1 mol l^−1^ Tris-Cl, 0.2 mol l^−1^ dithiothreitol, 4% SDS, 20% glycerol, 0.2% bromophenol blue, 4% β-mercaptoethanol) and boiled in 100°C water for 5 min. After centrifugation at 12 000*g* for 3 min, the supernatant was electrophoresed on acrylamide gels—the stacking gel and the separating gel were at 5% (v/v) and 10% (v/v), respectively. The gel for protein staining was treated with Coomassie Brilliant Blue R250. The gel was transferred to a PVDF membrane using an electrophoretic transfer cell for western blotting.

Western blotting was then performed using a mouse anti-His6 (TianGen, Beijing, China) (1:1000) or prepared mouse anti-BmYki1 (1:500) and HRP-conjugated goat anti-mouse IgG (Biosynthesis Biotechnology, Beijing, China) (1:2000). The protein band was visualized with 3, 3′-diaminobenzidine tetrahydrochloride (DAB) chromogenic substrate (2 mg DAB and 20 µl of 30% H_2_O_2_ in 10 ml of PBS).

To detect the expression of BmYki in different tissues, the proteins (about 20 µg per lane) from different tissues were separated with SDS-PAGE, and western blotting was performed with the prepared mouse anti-BmYki1 (1:500) and HRP-conjugated goat anti-mouse IgG.

### 2.5. Construction of transformed BmN cells overexpressing BmYki3

*BmYki3* was cloned into the KpnI/EcoRI sites of vector pIZT/V5-His (Invitrogen, Frederick, MD, USA) to generate recombinant plasmid pIZT/V5-His-*BmYki3*. The cultured BmN cells derived from silkworm ovaries were transfected with the recombinant plasmid using Lipofectin (Roche, Indianapolis, Germany) following the manufacturer's protocol. The cells were screened continuously for a month with zeocin (final concentration 200 µg ml^−1^) 3 days after transfection to generate BmN-Yki3 transformed cells. Simultaneously, control transformed cells (BmN-null) were generated by transfecting the pIZT/V5-His into BmN cells and screening with zeocin.

### 2.6. Cell wound healing assay

Cultured cells (5 × 10^5^) were added to a six-well plate. The cells were cultured overnight. A scratch wound was made across each well of the six-well plate using a pipette tip and washed three times with PBS to remove any loosely held cells. The cells were cultured in fetal bovine serum (FBS)-free TC-100 medium at 26.5°C. An image was taken every 6–12 h post-scratch to determine the migration speed of cells.

### 2.7. Cell proliferation

BmN cells (2 × 10^5^/ml, 2 ml) were added to a culture flask, after adherent culture for 24 h. Images were taken of five fixed views every 24 h to determine cell number.

### 2.8. Cell size

Cells (1×10^6^) were transfected with either pIZT/V5-His-*BmYki3* or pIZT/V5-His 24 h after transfection. The size of cells with and without fluorescence was determined every 24 h in the same culture flask.

### 2.9. Flow cytometry

Cells (1 × 10^3^) were cultured in FBS-free TC-100 medium (Gibco BRL, Rockville, MD, USA) at 26.5°C for 24 h; then the cells were cultured in the TC-100 insect medium supplemented with 10% FBS for 24 h. After this process was repeated two times, the cells were cultured in TC-100 medium supplemented with 10% FBS for 48 h and digested with trypsin. The collected cells were fixed with 70% ethanol for 4 h after the medium was removed. The fixed cells were washed with PBS buffer twice and then stained with 50 µg ml^−1^ propidium iodide. The cell cycle was detected at an excitation wavelength of 488 nm with a flow cytometer (Beckman Coulter, Fort Worth, USA).

### 2.10. Immunofluorescence

BmN cells were collected and fixed with 4% paraformaldehyde for 15 min, and then rinsed with 0.01 M PBST (0.05% Tween-20 in PBS) and incubated with the prepared mouse anti-BmYki1 antiserum at 4°C overnight. At the same time, BmN cells were incubated with pre-immune antiserum as a negative control. After rinsing with 0.01 M PBST three times, the cells were incubated with TRITC-conjugated goat anti-mouse IgG (Tiangen, Beijing, China) at 37°C for 1 h. The cells were then observed by a laser scanning confocal microscope (LEICA TCS SP5, Mennharm, Germany) after removing the non-combined TRITC-conjugated goat anti-mouse IgG and staining with DAPI.

### 2.11. RNAi

Cells (1 × 10^5^) were transfected with 1 µg Yki-siRNA-298 or GFP-siRNA-274 (electronic supplementary material, table S2), which was synthesized by Integrated Biotech Solutions Corporation (Shanghai, China). The total RNAs were extracted from the cells at 48 h post-transfection and cDNA was synthesized by reverse transcription to determine the relative expression level of the *Yki* gene to the *actin A3* gene with qPCR.

The *BmYki*-specific Yki-siRNA-298 was used to silence the *BmYki* gene *in vivo*. At 3 days after pupation, 22 pupae were injected with Yki-siRNA-298 (2 µg per pupa) below the right wing disc. The negative control group was treated with DEPC water (14 pupae) or GFP-siRNA-274 (8 pupae), while the pupa in the blank group was not injected.

### 2.12. Overexpressing the *BmYki3* gene

To investigate the effect of elevating the *BmYki* gene expression level in the wing disc, 10 pupae were injected with pIZT/V5-His-*BmYki3* below the right wing disc ((1 µg µl^−1^) × 2 μl, per pupa). The negative controls were injected with ddH_2_O (2 µl per pupa), while the pupa in the blank group was not injected.

### 2.13. Co-immunoprecipitation

Proteins extracted from the BmN-Yki3 transformed cells were pre-combined with Pierce Protein A/G Plus Agarose (Biotech, Shanghai, China) for 10 min at 4°C and centrifuged for 15 min at 15 300*g*. The total protein in the supernatant was quantified using the Bradford method and then incubated with anti-BmYki antibody at 4°C overnight. Simultaneously, the serum of a non-immune mouse was used as a control. Subsequently, the total protein–antibody complexes were incubated with Pierce Protein A/G plus Agarose at 4°C overnight. For SDS-PAGE, the total protein–antibody–protein A/G plus agarose complexes collected through centrifugation at 15 300*g* for 5 s were washed with 0.01 M PBS three times, mixed with 2× SDS loading buffer (0.2% bromophenol blue, 4% SDS, 20% glycerol, 0.2M dithiothreitol, 0.1M Tris-Cl, pH 6.8), and boiled for 5 min in a water bath. Following centrifugation for 3 min at 15 300*g*, the supernatant was subjected to SDS-PAGE with 5% spacer gel and 12% separation gel. The separated proteins on the gel were visualized by silver staining.

### 2.14. In-gel digestion, LC-MS/MS analysis and database search

To identify the proteins that interacted with BmYki3, the different protein bands in the SDS-polyacrylamide gel were recovered. In-gel digestion, LC-MS/MS analysis and database search were performed as in our previous report [20].

### 2.15. RNA-Seq and data analysis

To assess the regulation of BmYki3 on gene expression at the genome-wide level, RNA-Seq for BmN-Yki3 and BmN-null transformed cells were carried out following our previous report [21]. All data were submitted to the public database (accession no. SRR6315447). The differentially expressed genes (DEGs) between BmN-Yki3 and BmN-null transformed cells were further analysed with GO annotation and KEGG pathway data.

## 3. Results

### 3.1. Three isoforms of *BmYki* were generated by alternative splicing in silkworm

**Figure 1.**
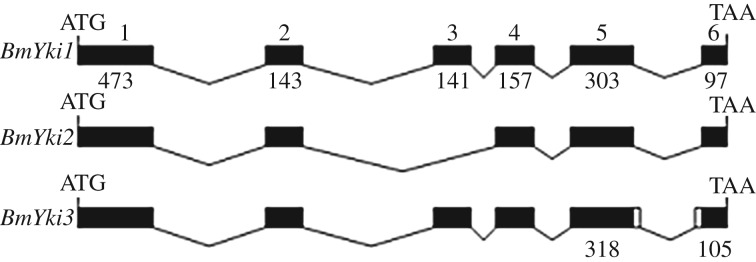
Three isoforms of *BmYki* are generated by alternative splicing in the silkworm. *BmYki1* (KF904339) has six exons 473, 143, 141, 157, 303 and 97 nt in length. Exon 3 of *BmYki1* is absent in *BmYki2* (KF904340), while exon 5 of *BmYki3* (KF904341) has an additional 15 nt at its 3′-end. Exon 6 of *BmYki3* has an additional 6 nt at its 5′-end when compared with the corresponding exons.

To clone the *BmYki* gene, the cDNA prepared from gonads was used as a template and PCR was carried out with primers BmYki-1 and BmYki-2 (electronic supplementary material, table S1). PCR products were cloned into a T-vector and sequenced. The results indicated that the *BmYki* gene had three different isoforms, *BmYki1* (KF904339), *BmYki2* (KF904340) and *BmYki3* (KF904341) that encoded 437, 390 and 444 amino acid residues, respectively. The cDNA sequence of *BmYki* was compared with the genomic DNA sequence. The results show that *BmYki*1 has six exons. Exon 3 of *BmYki1* was deleted in *BmYki*2, while exon 5 of *BmYki3* had an additional 15 nt at its 3′-end. Exon 6 of *BmYki3* had an additional 6 nt at its 5′-end compared with the corresponding exons (figure 1). Conserved domains were predicted by online software (http://au.expasy.org/prosite/). The results showed that both the BmYki1 and BmYki3 proteins had two WW domains which were located at 138–171 aa and 219–252 aa, respectively, while the BmYki2 protein had one WW domain (138–171 aa). The amino acid sequence of BmYki was similar to that of the *Drosophila* Yki, suggesting that the function of the Yki protein is conserved between *Bombyx* and *Drosophila*.

### 3.2. Expression level of *BmYki3* positively correlated with tissue size

**Figure 2.**
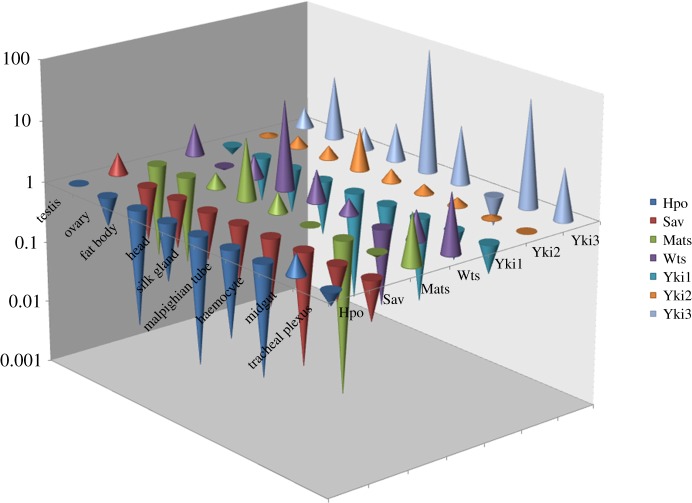
Relative abundance of major genes in the Hippo pathway in diverse tissues at day 3 of the fifth instar stage. The expression level of *BmHpo* in testis is assumed to be 1. The values of the 2^−ΔΔCT^ were transformed into Log 10 data for analysis.

To understand the relationship between the *BmYki* gene expression level and tissue size, the relative expression level of *BmYki* in different tissues on day 3 of the fifth instar were determined with qPCR. The results generally showed that *BmYki1* was weakly expressed in the tissues assessed, and the lowest level was in the silk gland. The expression level of *BmYki2* in different tissues was lower compared with *BmYki3*, with the highest level in the head. *BmYki3* was highly expressed in the silk gland and midgut, and was weakly expressed in the haemocyte (figure 2). Both midgut and silk gland were the largest tissues at day 3 of the fifth instar. The *BmYki3* gene expression level positively correlated with tissue size.

**Figure 3.**
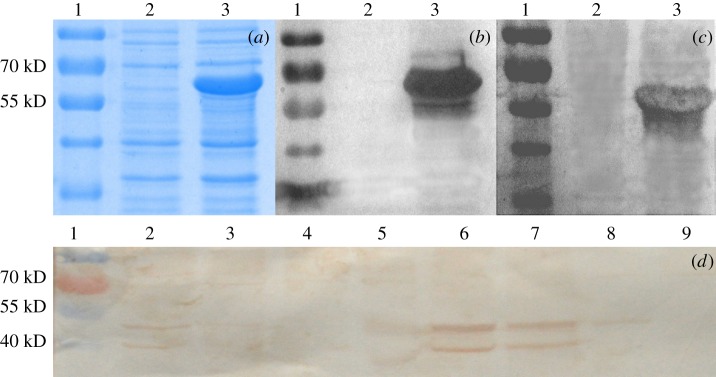
Expression of recombinant BmYki1 in *E. coli* and detection of BmYki in the different tissues of the silkworm. (*a*) SDS-PAGE for the recombinant BmYki1 expressed in *E. coli*, Lane 1, protein marker; Lane 2, transformed *E. coli* Transetta (DE3) strain with pET-28a(+); Lane 3, transformed *E .coli* Transetta (DE3) strain with pET-28a(+)-*BmYki1*. (*b*) Western blotting for recombinant BmYki1 expressed in *E. coli*; Lanes 1–3 are as described above. Mouse anti-His6 antibody was used as primary antibody; the secondary antibody was HRP-conjugated goat anti-mouse IgG. (*c*) Mouse anti-BmYki1 antibody was used to detect the recombinant BmYki1 expressed in *E. coli* with western blotting; the secondary antibody was HRP-conjugated goat anti-mouse IgG; Lane 1, protein marker; Lane 2, transformed *E. coli* Transetta (DE3) strain with pET-28a(+); Lane 3, transformed *E.coli* Transetta (DE3) strain with pET-28a(+)-*BmYki1*; (*d*) Detection of BmYki in the different tissues at day 3 of the 5th instar by western blotting; Lane 1, protein marker; Lane 2, testis; Lane 3, ovary; Lane 4, head; Lane 5, midgut; Lane 6, silk gland; Lane 7, fat body; Lane 8, malpighian tube; Lane 9, tracheal plexus. The primary antibody was a mouse anti-BmYki antibody and the secondary antibody was HRP-conjugated goat anti-mouse IgG.

Moreover, to detect the BmYki protein in different tissues using western blotting with anti-BmYki antibody, the recombinant BmYki1 protein expressed in *E. coli* was used to immunize mice by subcutaneous injection. Western blotting was used to assess the efficacy of the prepared mouse anti-BmYki1 antibody. As expected, a specific band representing the 6×His-BmYki1 fusion protein was also detected, indicating that the prepared antibody was suitable for further studies (figure 3).

The total proteins from different tissues at day 3 of the fifth instar were detected using western blotting using a prepared anti-BmYki1 antibody. A specific band (approx. 50 kDa) representing BmYki1 and BmYki3 could be detected in testis, ovary, midgut, silk gland and fat body, while a specific band (approx. 40 kDa) representing BmYki2 could be observed in testis, silk gland and fat body (figure 3).

### 3.3. *BmYki3* has the opposite expression pattern to upstream genes of the Hippo pathway

The core genes of the Hippo pathway consist of *Hpo*, *Sav*, *Wts* and *Mats*. Sav and Mats are the adaptor proteins of Hpo and Wts, respectively. The relative expression levels of the *Hpo* and *Sav* genes were lower in the different tissues on day 3 of the fifth instar. The *Wts* expression level was highest in the head, followed by the tracheal plexus; the *Mats* expression level was higher in the head and tracheal plexus, and was lower in other detected tissues. The comparison of expression level among Hippo pathway genes revealed that the expression pattern of *BmYki3* was nearly the opposite to that of the *Hpo* and *Sav* genes (figure 2).

### 3.4. BmYki protein was mainly located in the cytoplasm of cultured BmN cells

Immunostaining of the cultured BmN cells was performed with mouse anti-BmYki1 antibody, followed by treatment with TRITC-conjugated goat anti-mouse IgG; the BmYki1 protein (red) was mainly observed in the cytoplasm (electronic supplementary material, figure S1).

### 3.5. Cell size, cell migration and cell proliferation could be facilitated by BmYki3

**Figure 4.**
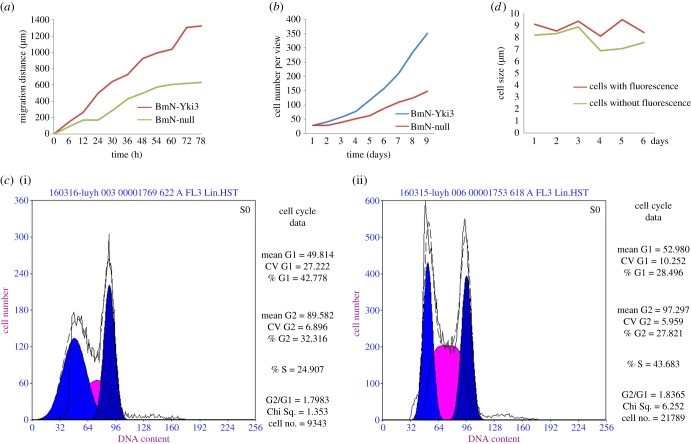
Effect of overexpression of the *BmYki3* gene on cell size, cell migration and cell proliferation. (*a*) Effect of overexpression of the *BmYki3* gene on cell migration; BmN-Yki3, the transformed cells overexpressing the *BmYki3* gene, BmN-null, the transformed cells with pIZT/V5-His. (*b*) Effect of overexpression of the *BmYki3* gene on cell proliferation. (*c*) Effect of overexpression of the *BmYki3* gene on cell cycle: (i) BmN-null transformed cells and (ii) BmN-Yki3 transformed cells. (*d*) Effect of overexpression of the *BmYki3* gene on cell size, the BmN cells were transfected with pIZT/V5-His-*BmYki3*, the size of cells with/without fluorescence was determined daily.

To assess the regulation of BmYki3 expression level in cultured BmN cells, BmN-Yki3 and BmN-null transformed cells were constructed. Western blotting results showed that the level of BmYki3 in BmN-Yki3 cells was significantly higher than in BmN-null cells (electronic supplementary material, figure S2). A cell wound healing assay showed that the migration rate of BmN-Yki3 transformed cells was higher than that of BmN-null transformed cells (figure 4*a*). The cell number per view was investigated to assess the cell proliferation. The initial value of cell number per view was similar between BmN-Yki3 and BmN-null transformed cells; 9 days later, the number of BmN-Yki3 transformed cells per view increased by 2.37 times (figure 4*b*). The cell ratios of the G1, S and G2 phases were estimated by flow cytometer. Synchronized cells were cultured in TC-100 medium supplemented with 10% FBS for 48 h. The results showed that the ratios of the G1, S and G2 phases were 43%, 25% and 32% in BmN-null transformed cells (figure 4*c*(i)), and 28%, 44% and 28% in BmN-Yki3 transformed cells (figure 4*c*(ii)), suggesting that BmYki3 could facilitate cell proliferation. Moreover, the size of cells with and without fluorescence was investigated at 2, 3, 4, 5 and 6 days post-transfection with pIZT/V5-His-*BmYki3*. The results indicated that the size of cells with fluorescence was larger than cells without fluorescence, suggesting that BmYki3 could increase the cell size (figure 4*d*).

In addition, the effect on cell size of silencing the *BmYki* gene in the cultured BmN cells was also investigated. After being transfected with the specific siRNA of the *BmYki* gene, *BmYki* gene expression level was assessed with qPCR. The results showed that siRNA-298 of *BmYki* had the best gene silencing efficacy. The *BmYki1/3* gene expression level was decreased by 8.25 times compared with GFP-siRNA expression. Using western blotting, we also detected a lower expression level of BmYki3 in cells transfected with Yki-siRNA-298 (electronic supplementary material, figure S3). Observations by microscopy showed that the size of the cultured BmN cells treated with Yki-siRNA-298 became smaller than that of cells treated with GFP-siRNA-274 (electronic supplementary material, figure S4).

### 3.6. BmYki3 enlarged the wing size

**Figure 5.**
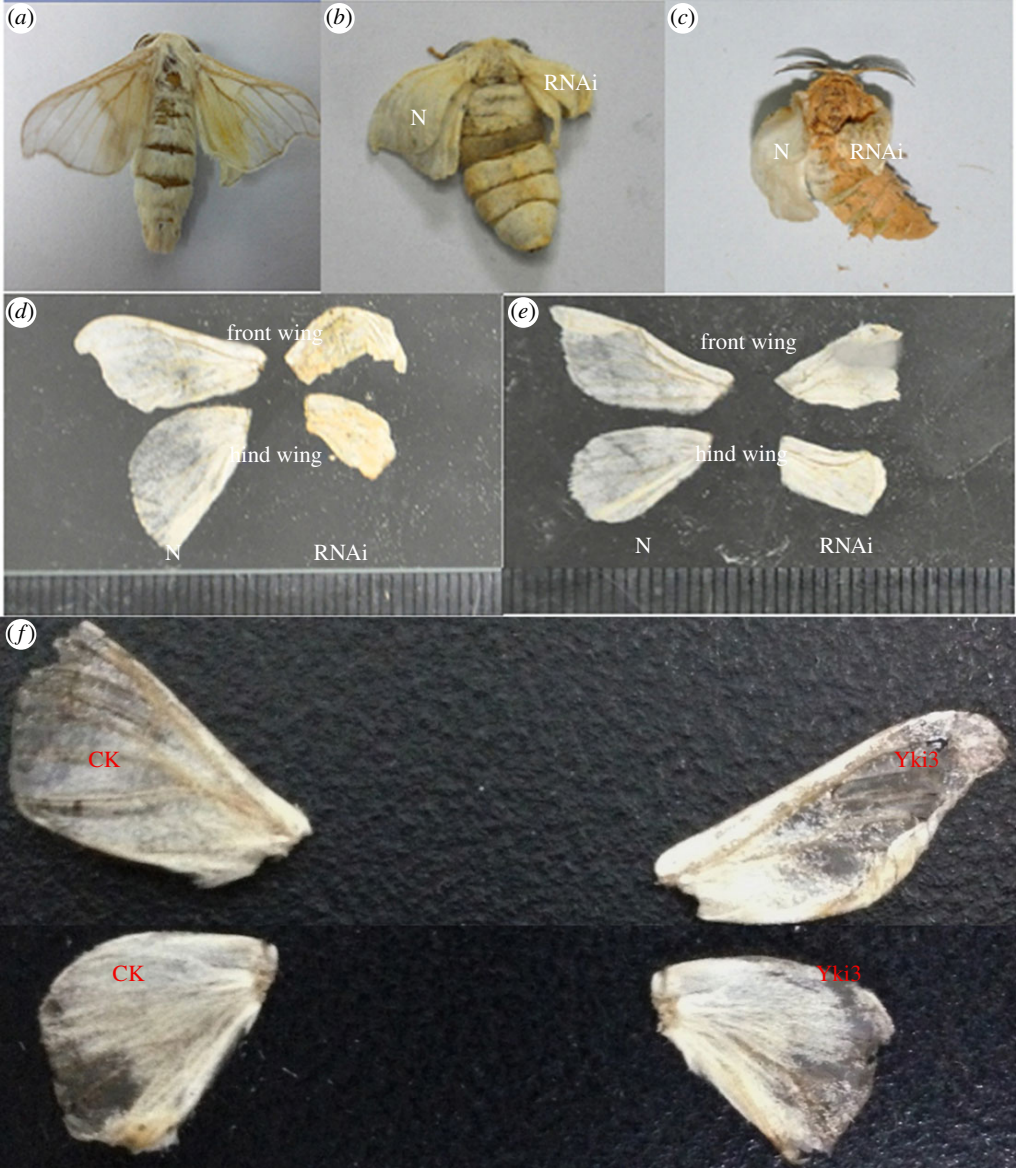
Effects of silencing the *BmYki* gene on wing disc development of pupae. (*a*) normal moth wing. (*b*) A moth which was injected with Yki-siRNA-298 and GFP-siRNA-274 below the right and left wing disc, respectively, at day 3 of pupation; RNAi, injected with Yki-siRNA-298; N, injected with GFP-siRNA-274. (*c*) A moth which was injected with Yki-siRNA-298 and DEPC-treated water below the right and left wing disc, respectively, at day 3 of pupation; RNAi, injected with Yki-siRNA-298; N, injected with DEPC-treated water. (*d*,*e*) Comparison of the size between left and right wings which were injected with GFP-siRNA-274 (DEPC-treated water) and Yki-siRNA-298, respectively, at day 3 of pupation; N, injected with GFP-siRNA-274 or DEPC-treated water; RNAi: injected with Yki-siRNA-298. (*f*) Comparison of the size between left and right wings which were injected with DEPC-treated water and pIZT/V5-His-*BmYki3*, respectively, at day 3 of pupation; CK, DEPC-treated water; Yki3: injected with pIZT/V5-His-*BmYki3*.

To understand the effects of silencing the *BmYki* gene on the development of the wing disc, the right and left wing discs of 22 pupae were injected with Yki-siRNA-298 (1 µg for each) and GFP-siRNA-274 (8 pupae) or DFPC-treated water (14 pupae). At day 3 of pupation, the ratio of the atrophied right wing reached 36% (table 1, figure 5). The length of the front and hind wings for the Yki-siRNA-298 treatment declined by >9% and 28%, respectively, compared with the control group. Moreover, the right and left wing discs of 10 pupae were injected with pIZT/V5-His-*BmYki3* and water, respectively. Approximately 30% of the front wing increased by more than 7% in length in the group injected with pIZT/V5-His-*BmYki3*, while the abnormal wing was not observed in the group injected with water. These results indicated that BmYki3 could enlarge the wing disc size (table 1, figure 5)

**Table 1.** Effects of down/upregulation of *BmYki* gene expression on the size of the wing disc. Serial numbers 1–5, at day 3 of pupation, the pupae were, respectively, injected with DEPC-treated water and Yki-siRNA-298 below the left and right wings. Serial numbers 6–8, at day 3 of pupation, the pupae were, respectively, injected with GFP-siRNA-274 and Yki-siRNA-298 below the left and right wings. Serial numbers 9–11, at day 3 of pupation, the pupae were, respectively, injected with DEPC-treated water and pIZT/V5-His-*BmYki3*.

serial number	length of the left front wing (cm)	length of the right front wing (cm)	atrophy ratio of length for the front wing	length of the left hind wing (cm)	length of the right hind wing (cm)	atrophy ratio of length for the hind wing
1	1.8	1.4	−22.2%	1.3	0.9	−30.8%
2	1.8	1.3	−27.8%	1.3	0.9	−30.8%
3	1.7	1.3	−23.5%	1.2	0.5	−58.3%
4	1.7	1.2	−29.4%	1.2	0.7	−41.7%
5	1.5	1	−33.3%	1.2	0.8	−33.3%
6	1.5	1	−33.30%	1.3	0.9	−30.70%
7	1	0.7	−30.00%	0.7	0.5	−28.57%
8	1.1	1	−9.09%	0.9	0.3	−66.67%
9	1.3	1.4	7.7%	0.9	0.9	0%
10	1.8	2.0	11.1%	1.2	1.2	0%
11	1.4	1.6	14.3%	1.0	1.0	0%

### 3.7. Expression of genes related to the Hippo pathway, cell cycle and innate immunity could be regulated by silencing *BmYki*

**Figure 6.**
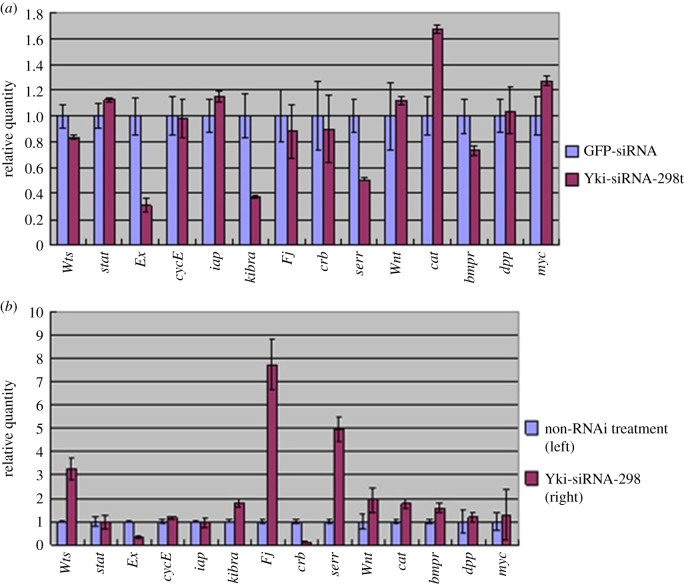
Relative expression of genes related to the Hippo pathway in BmN cells and wing discs when *BmYki1* genes are silenced. (*a*) Relative expression in the cultured cells; GFP-siRNA, the cells treated with GFP-siRNA-274; Yki-siRNA-298, the cells treated with Yki-siRNA-298. (*b*) Relative expression in wing discs; the pupa was injected with GFP-siRNA-274 and Yki-siRNA-298 below the left (no siRNA treatment) and right (Yki-siRNA-298) wing discs, respectively, at day 3 of pupation.

To understand how the Hippo pathway regulates cell size, the expression levels of the upstream regulatory genes of the canonical Hippo pathway and genes related to the innate immunity pathway, cell cycle and apoptosis in BmN cells were determined by qPCR after *BmYki* was silenced with Yki-siRNA-298. The results showed that the expression levels of *Ex* and *kibra* genes decreased by 2.25 and 1.67 times, respectively, while the *cat* gene expression level increased by 1.65 times. Significant changes in expression levels were not found in the other genes assessed compared with the control group treated with GFP-siRNA-274 (figure 6*a*). Furthermore, the gene expression pattern was investigated after *BmYki* was silenced in wing discs. The expression level of the *BmYki1/3* genes in the right wing treated with GFP-siRNA-274 decreased 2.76-fold compared with that in the left wing. At the same time, the expression levels of *Ex* and *crb* genes decreased by 1.95 and 8.20 times, while the expression levels of *Wts*, *Fj* and *serr* genes were significantly upregulated (figure 6*b*), indicating that the size of the wing discs can be regulated by the Hippo signalling pathway.

### 3.8. Expression of genes related to the cell cycle, signalling pathways, innate immunity, apoptosis, phagocytosis and hormone biosynthesis could be regulated by overexpressing *BmYki3*

RNA-Seq was carried out to provide a more comprehensive assessment of the changes in cultured cells by overexpressing *BmYki3*. A total of 71 056 856 and 75 800 310 paired-end reads with an average length of 122.39 bp and 123.98 bp were obtained from BmN-null and BmN-Yki3 transformed cells, respectively. The ratios of clean reads were 99.67% and 99.60% for BmN-null and BmN-Yki3 cells, respectively. *Bombyx mori* EST sequences were downloaded from GenBank and 30 646 mRNAs were obtained.

All clean data were mapped to the 30 646 mRNAs sequences using the TopHat software [22]. A total of 26 680 and 26 092 expression genes were found in BmN-null and BmN-Yki3 cells, respectively. RPKM (reads per kilobase of exon model per million mapped reads) was used to assess the gene expression level. DEGs were identified by a fold change (FC) value (FC ≥ 1 or FC ≤ −1); 4444 genes were upregulated and 10 291 genes were downregulated after *BmYki3* was overexpressed in cultured cells. Using more stringent criteria (Fisher test, *p* ≤ 0.05), 1033 and 4156 genes were up- and downregulated (electronic supplementary material, figure S5). The expression of seven selected DEGs in BmN-null and BmN-Yki3 cells was confirmed by qPCR to validate the RNA-Seq results. This result suggested that the RNA-Seq data were credible (electronic supplementary material, figure S6).

**Figure 7.**
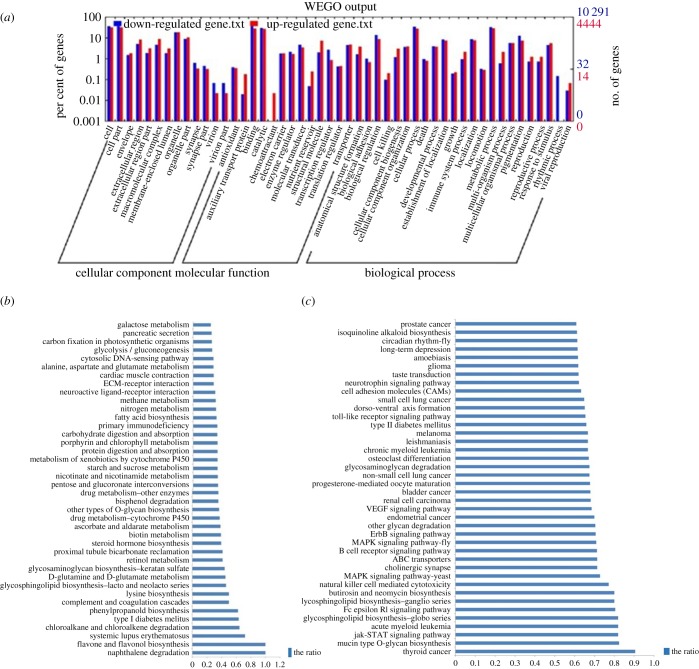
GO and KEGG analysis of DEGs. (*a*) GO analysis of DEGs defined by FC-test. (*b*) KEGG enrichment analysis for upregulated genes (top 50 pathways); (*c*) KEGG analysis for downregulated genes (top 50 pathways).

The possible functions of all DEGs (FC ≥ 1 or FC ≤ −1) were determined by using the GO classification system. All DEGs were involved in 48 GO categories (figure 7*a*). GO enrichment analysis showed that the up- and downregulated genes with GO annotation were enriched in 441 (268) and 537 (382) GO terms, respectively, *p* ≤ 0.05 (0.01) (electronic supplementary material, tables S3 and S4). The GO annotation of all the genes included phagocytosis, the G1/S transition of mitotic cell cycle, ecdysone receptor-mediated signalling pathway, cell morphogenesis, phosphatidylinositol 3-kinase complex, Rho GTPase binding, mitogen-activated protein kinase kinase kinase binding, target of rapamycin (TOR) signalling cascade and negative regulation of JAK-STAT cascade. A total of 133 GO terms were downregulated; 55.82% of the genes were related to the G-protein-coupled receptor signalling pathway, 81.25% of the genes were related to juvenile hormone esterase activity, 69.44% of the genes were related to the regulation of the cell cycle, 83.33% of the genes were involved in the regulation of G2/M transition of the mitotic cell cycle, 64.71% of the genes were related to the regulation of cyclin-dependent protein kinase activity, 80% of the genes were related to cell cycle arrest and 66.67% of the genes were related to the canonical Wnt receptor signalling pathway.

All genes with GO annotation related to the negative regulation of cell death, positive regulation of oxidative phosphorylation uncoupler activity, response to lipopolysaccharide and negative regulation of juvenile hormone biosynthetic process were upregulated; 51.80%, 44.75%, 75%, 66.67%, 39.13%, 60%, 60%, 60% and 50% of genes in defence response to bacterium, innate immune response, regulation of innate immune response, cellular response to reactive oxygen species, defence response to fungus, inactivation of MAPK activity, negative regulation of MAPK cascade, negative regulation of JNK cascade and induction of apoptosis by extracellular signals, respectively, were downregulated.

To further explore the functions of DEGs, KEGG enrichment analysis was assessed. Up- or downregulated genes were enriched in 49 or 101 pathways, respectively (*p* ≤ 0.01). Enriched pathways for the upregulated genes included steroid hormone biosynthesis, protein digestion and absorption, fatty acid biosynthesis, proteasome, oxidative phosphorylation and pyruvate metabolism (figure 7*b*). The enriched pathways for the downregulated genes included the JAK-STAT signalling pathway, natural killer cell-mediated cytotoxicity, MAPK signalling pathway, ErbB signalling pathway, pathways for several cancers, type II diabetes mellitus, Toll-like receptor signalling pathway, chemokine signalling pathway, GnRH signalling pathway, Fc gamma R-mediated phagocytosis, apoptosis, p53 signalling pathway, Wnt signalling pathway and insect hormone biosynthesis (figure 7*c*).

### 3.9. BmYki could interact with proteins related to immunoregulation, apoptosis, cell motility and cell cycle

In *Drosophila*, Yki can interact with transcription factors in the nucleus and lead to the transcriptional upregulation of a series of target genes [5]. Phosphorylated Yki can interact with cytoskeletal protein 14-3-3, which resulted in Yki being detained in the cytoplasm [8]. In the present study, the proteins that interacted with BmYki in the cultured BmN cells were screened with co-immunoprecipitation (CoIP) (electronic supplementary material, figure S7). Results identified by LC-MS/MS indicated that BmYki could interact with thymosin, protein kinase ASK1, E3 ubiquitin-protein ligase RNF123-like, dedicator of cytokinesis protein 1, transmembrane protein 151B-like, phosphatidylinositol 4-kinase alpha, calcium-independent phospholipase A2, Ccr4–Not transcription complex subunit 7 and putative beta-spectrin (table 2), suggesting that BmYki could interact with proteins related to immunoregulation, apoptosis, cell motility and the cell cycle.

**Table 2.** Proteins that interact with BmYki3.

serial number	protein description	protein accession	Mw (kDa)	pI
1	very long-chain specific acyl-CoA dehydrogenase, mitochondrial [*Bombyx mori*]	gi|827550011	66769.5	8.37
2	thymosin isoform X1 [*Bombyx mori*]	gi|827556818	23428.9	5.09
3	protein kinase ASK1 isoform X1 [*Bombyx mori*]	gi|827553804	157048.5	5.85
4	thymosin isoform X2 [*Bombyx mori*]	gi|827556820	19175.6	4.95
5	general odorant-binding protein 99a isoform X2 [*Bombyx mori*]	gi|827551251	16956.3	5.64
6	LOW QUALITY PROTEIN: alcohol dehydrogenase 2-like [*Bombyx mori*]	gi|827536287	24707.6	5.62
7	E3 ubiquitin-protein ligase RNF123-like [*Bombyx mori*]	gi|827545133	80752.5	6.43
8	thymosin isoform X3 [*Bombyx mori*]	gi|827556822	19152.8	5.02
9	protein kinase ASK1 [*Bombyx mori*]	gi|320202939	153593.7	5.79
10	pheromone-binding protein precursor [*Bombyx mori*]	gi|112984442	18415.8	4.86
11	uncharacterized protein LOC101739296 [*Bombyx mori*]	gi|512896558	30551.5	8.34
12	arginine–tRNA ligase, cytoplasmic	gi|512928912	78541.8	6.35
13	5-oxoprolinase [*Bombyx mori*]	gi|827538375	139527.7	5.9
14	dedicator of cytokinesis protein 1 isoform X3 [*Bombyx mori*]	gi|827537387	220309.7	6.97
15	transmembrane protein 151B-like [*Bombyx mori*]	gi|827551130	72905.4	8.55
16	dedicator of cytokinesis protein 1 isoform X2 [*Bombyx mori*]	gi|827537385	223900.7	7.2
17	dedicator of cytokinesis protein 1 isoform X1 [*Bombyx mori*]	gi|827537383	224355.9	7.12

## 4. Discussion

The Hippo signalling pathway, a signalling pathway that inhibits cell growth, is very conserved in the process of biological evolution [6]. Most components of the Hippo pathway have been identified from genetic screens in *Drosophila*. In silkworm, we have identified major components related to the Hippo pathway including BmHop, BmSav, BmMats, BmWts and BmYki. Although these protein sequences did not have high identities with corresponding proteins from different species, the conserved domains were prominent, suggesting that the roles of the Hippo pathway in silkworm are similar to those in other species [16].

Yki is a transcriptional co-activator protein with a WW domain that is negatively regulated by Hippo signalling, which promotes its cytoplasmic localization [23]. In *Drosophila*, there are four Yki isoforms, while three isoforms were found in the silkworm; both BmYki1 and BmYki3 have two WW domains, but BmYki2 has one WW domain. The WW domain binds to proteins with a particular proline-motif and/or a phosphoserine or phosphothreonine-containing motif, suggesting that BmYki has a transcriptional activation ability depending on the isoform.

qPCR results showed that there was a variation in the expression levels of major genes related to the Hippo pathway in different tissues at day 3 of the fifth instar in the silkworm, suggesting that the Hippo signalling pathway was related to the development of tissue. The expression of several genes, including *BmYki*, *Bmmats*, *Bmwts*, *Bmsav* and *BmHpo*, was low in haemolymph, which may indicate that the haemocytes were well differentiated and there was no further increase in size. Silk gland cells do not divide but are enlarged during the larval stage. An increase in the size of silk glands began mainly from day 3 of the fifth instar, and the expression level of *BmYki3* in the silk gland at that stage was higher. We therefore deduced that the enlargement and development of silk glands could be regulated by the Hippo pathway. The midgut is the largest tissue. The midgut cells are obviously enlarged during the fifth instar because the larvae consume most mulberry leaves for the synthesis of silk protein at this stage. Therefore, we suggest that the enlargement of the midgut cells was also regulated by the Hippo signalling pathway because *BmYki3* was highly expressed.

Interestingly, the expression level of *BmYki3* was obviously higher than that of *BmYki1* and *BmYki2* overall, and organ size positively correlated with the expression level of *BmYki3*, suggesting that organ size was mainly regulated by BmYki3. The different isoforms of BmYki may competitively bind to their interaction proteins to regulate the signalling of the Hippo pathway and expression of target genes. Moreover, the expression level of the *BmHpo* gene was generally opposite to that of *BmYki3*, suggesting that *BmHpo* could be negatively regulated by *BmYki3* through a feedback process.

Yki, the most important element in the Hippo signalling pathway, plays an important role in promoting cell growth and proliferation and in the inhibition of apoptosis [3].

YAP, homologues of *Drosophila* Yki, promote an early neural crest phenotype and migration [24]. The BmN cell line was derived from the ovary of the silkworm; in the present study, we found that the migration rate of transformed cells overexpressing *BmYki3* was higher and the cilia- and flagella-associated protein 44 gene (*Cfap44*) related to cilium-dependent cell motility [25] was upregulated compared with control cells, suggesting that an increase of cell migration ability may be related to the upregulation of *Cfap44* in cultured cells overexpressing *BmYki3*.

Yap plays an important role in controlling the size of tissues and organs. In the silkworm, BmYki1 facilitates organ growth [17]. Overexpression of BmYki1^CA^ in the posterior silk gland significantly increased the weight of the posterior silk gland [18]. We found that wing size was atrophied by silencing the *BmYki* gene and increased by overexpressing *BmYki3* at day 3 of pupation, and BmYki3 facilitated the proliferation and cycle of cultured BmN cells, suggesting that the organ is enlarged by virtue of its action on proliferation and/or size of the cell. KEGG analysis showed that the upregulated genes were enriched in protein digestion and absorption, carbohydrate digestion and absorption, fatty acid biosynthesis, glycolysis, pyruvate metabolism and oxidative phosphorylation. These upregulated pathways provide nutrients and energy for proliferation and enlargement of cells. Furthermore, we also found that the wing was huddled after *BmYki* expression was inhibited by RNAi. It was reported that organ growth is controlled by patterning signal proteins, including Wingless/Ints, bone morphogenetic proteins and Hedgehogs, and scaled by nutrient-dependent signals including insulin-like peptides transduced by the TOR pathway [26]. In this study, we found that the insulin, Wnt, mTOR and Hedgehog signalling pathways were inhibited in the cultured cells overexpressing *BmYki3*, which may be involved in wing shape. Wings are a tool of migratory movement for flying pests, therefore silencing the *Yki* gene in wing discs may be a novel strategy for interfering with pest migration and reducing the level of threat.

Recent studies have found that YAP negatively regulates an antiviral immune response. YAP deficiency results in enhanced innate immunity [27]. In the present study, we found that downregulated genes in the cultured cells overexpressing the *BmYki3* gene were enriched in the defence response to bacteria, innate immune response, regulation of innate immune response and defence response to fungus GO terms, while JAK-STAT, Fc epsilon RI, B cell receptor and Toll-like receptor signalling pathways, which are related to immunity or resistance to pathogens, were downregulated. Therefore, we predict that the antiviral immune system in silkworm may be negatively regulated by enhancing the expression of *BmYki3*.

Expanded (Ex) functions as an inhibitor of growth, and Hippo signalling suppresses the transcription of *Ex* [26]. Ex associates with the 14-3-3 proteins to sequester Yki in the cytoplasm, inhibiting the growth activity of the Hippo pathway [11]. In the present study, we found that *Ex* gene expression level in both cultured cells and wing discs decreased after silencing the *BmYki* gene, suggesting that the fine-tuned homeostasis of tissue during development could be maintained by the Hippo pathway via regulating *Ex*.

The activity and stability of several Hippo pathway components are regulated by ubiquitin-mediated protein turnover [28]. The coIP results in our study showed that BmYki3 could interact with E3 ubiquitin-protein ligase, suggesting that the degradation of BmYki3 could be mediated by ubiquitin.

The development of silkworm is controlled by JH and ecdysone; our results indicate that the expression level of *JH epoxide hydrolase* gene is associated with the degradation of JH [29]. However, the expression level of the *ecdysteroid 22-kinase* gene, which is responsible for the phosphorylation of ecdysteroids to form physiologically inactive ecdysteroid 22-phosphates, was upregulated by overexpression of *BmYki3* [30]. Therefore, we speculate that BmYki3 could postpone the development of the silkworm and result in the enlargement of tissues.

Recently, a high-confidence *Drosophila* Hippo protein–protein interaction network (Hippo-PPIN) consisting of 153 proteins and 204 interactions was generated by mass spectrometry using existing pathway components as baits [31]. In this study, we found BmYki may interact with thymosin, protein kinase ASK1 (known as mitogen-activated protein kinase kinase kinase 5, MAP3K5), E3 ubiquitin-protein ligase RNF123-like, dedicator of cytokinesis protein 1, transmembrane protein 151B-like, phosphatidylinositol 4-kinase alpha, calcium-independent phospholipase A2, Ccr4–Not transcription complex subunit 7 and putative beta-spectrin, which may result in alteration of the location, stability and activity of BmYki. It was reported that the MAP4K family kinases act in parallel to MST1/2 to activate LATS1/2 in the Hippo pathway [32]. BmYki3 could interact with MAP3K5, so we speculated that MAP3K5 has a similar function in the silkworm. The Ccr4–Not complex is a unique, essential and conserved multi-subunit complex that acts at many different levels of cellular function to regulate gene expression. The Ccr4–Not complex might function as a ‘chaperone platform’ [33]. In this study, we found that BmYki3 could bind to the Ccr4–Not transcription complex subunit 7, suggesting that expression of the target gene of the Hippo signalling pathway could be regulated by the interaction of BmYki3 with Ccr4–Not.

To further understand the regulation mechanism of BmYki3, the genome-wide change in gene transcriptional level after overexpressing the *BmYki3* gene was assessed by RNA-Seq. We found 14 735 DEGs were enriched in 650 GO terms (*p* ≤ 0.01) and 150 KEGG pathways (*p* ≤ 0.01). In general, the number of downregulated genes was 2.32 times higher than the number upregulated. This result provides insight into a comprehensive understanding of the Hippo pathway in the silkworm.

Some genes of other signalling pathways, including ligands for Notch, Wnt, EGFR and JAK-STAT pathways, were targets of the Hippo pathway [34–37]. In the present studies, we found that the MAPK, T cell receptor, insulin, p53, Hedgehog, adipocytokine, Wnt, calcium and PPAR signalling pathways could be regulated by the Hippo pathway in the silkworm.

Moreover, previous studies indicate that several upstream components (Ex, Mer, Kibra, Crb and Fj) of the Hippo pathway are also downstream targets of the Hippo pathway, providing a negative feedback mechanism to regulate the status of the Hippo pathway precisely [38]. In the present study, we found similar results in the silkworm.

## Supplementary Material

supplementary figures and Tables

## Data accessibility

Additional data are available as the electronic supplementary material.
