# Supplementary material for: Cultured cells and wing disc size of silkworm can be controlled by the Hippo pathway
Source: Open Biol. 2018 Jul 4;8(7):180029. doi: 10.1098/rsob.180029 (PMC6070717; doi:10.1098/rsob.180029)
Supplement: supplementary figures and Tables [file rsob180029supp1.zip › supplementary figures and tables/supplementary figures.docx]

**Cultured cells and wing disc size of silkworm can be controlled by Hippo pathway**

OPEN BIOLOGY. ID: RSOB-18-0029.R1

Zi Liang^a,c,1^Yahong Lu^a,c,1^, Ying Qian^a,c,1^, Liyuan Zhu^a,c^, Sulan Kuang^a,c^, Fei Chen^a,c^,Yongjie Feng^a,c^, Xiaolong Hu^a,b^, Guangli Cao^a,b^, Renyu Xue^a,b*^, Chengliang Gong^a,b,c*^

^a^School of Biology & Basic Medical Science, Soochow University, Suzhou 215123, China

^b^National Engineering Laboratory for Modern Silk, Soochow University, Suzhou, PR China

^C^Agricultural Biotechnology ResearchInstitute, Agricultural biotechnology and Ecological Research Institute, Soochow University, Suzhou, 215123, China

^1^These authors contributed equally to this work.

*Corresponding author: Tel: +86-512-65880183; Fax: +86-512-65880183.

E-mail address: xuery@suda.edu.cn, gongcl@suda.edu.cn


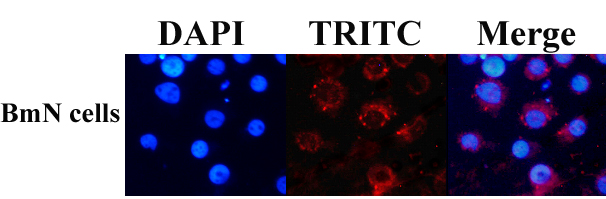


**Figure S1**

**Figure S1 Cellular location of BmYki (Magnification: 10×40).**

The nucleus was stained with DAPI (blue), BmYki was stained with TRITC (Red).

**70kDa**

**70kDa**

**55kDa**

**55kDa**


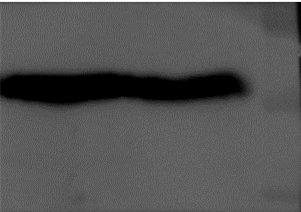

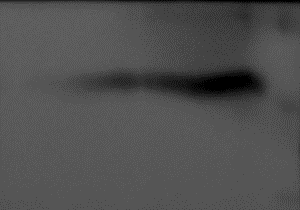


**BmN-Yki3**

**BmN-null**

A

B

**Figure S2**

**Figure S2 Expression level of BmYki3 in BmN-Yki3 and BmN-null transformed cells.**

Western blotting of BmYki3 after overexpressing *BmYki3* in cells, the secondary antibody was HRP-conjugated goat anti-mouse IgG (1:10000).

A, Mouse anti-alpha Tubulin antibody was used as primary antibody (1:10000); B, Mouse anti- BmYki3 antibody was used as primary antibody (1:1000).

**70kDa**

**70kDa**

**55kDa**

**55kDa**


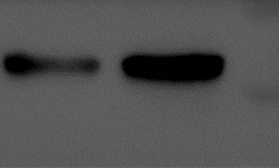

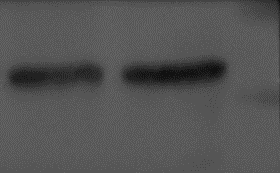


**Yki-siRNA-298**

**GFP-siRNA-274**

A

B

**Figure S3**

**Figure S3 Expression level of BmYki3 in cells treated with Yki-siRNA-298 or GFP-siRNA-274**

Western blotting of BmYki3 after silencing *BmYki3* in cells, the secondary antibody was HRP-conjugated goat anti-mouse IgG (1:10000).

A, Mouse anti-alpha Tubulin antibody was used as primary antibody (1:10000); B, Mouse anti- BmYki3 antibody was used as primary antibody (1:1000).


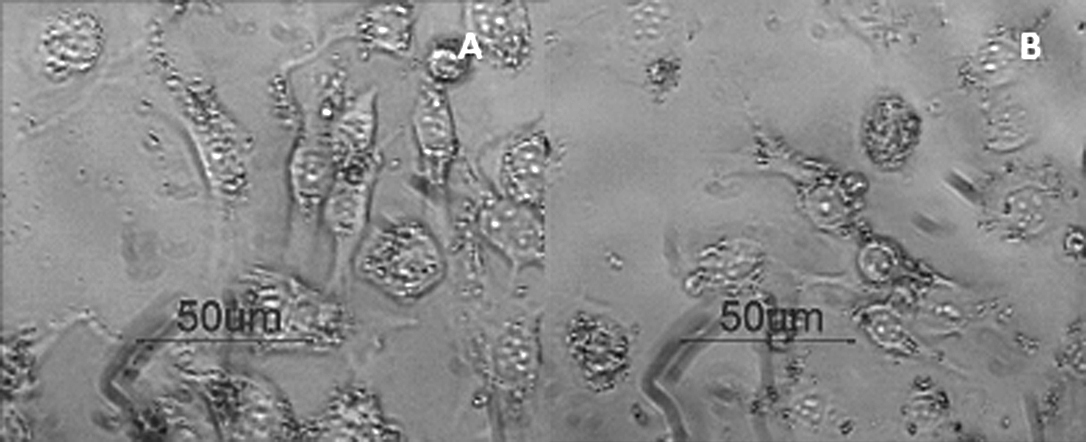


**Figure S4**

**Figure S4 Effect of silencing Bmyki gene on cell size.**

A, cells treated with GFP-siRNA-274; B, cells treated with Yki-siRNA-298.


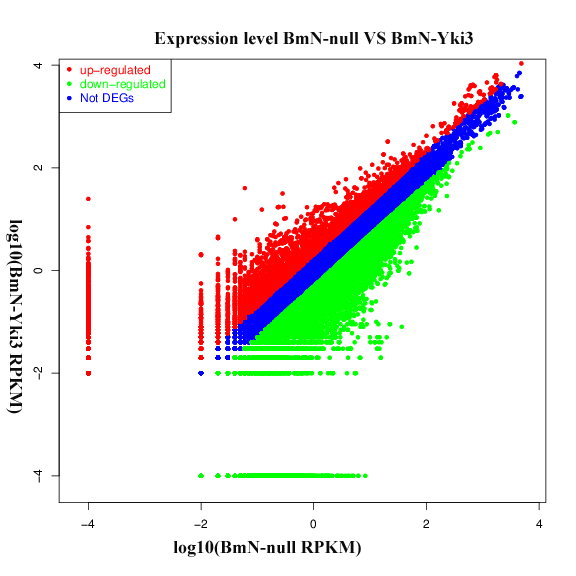


**Figure S5**

**Figure S5 Differentially expressed genes.**


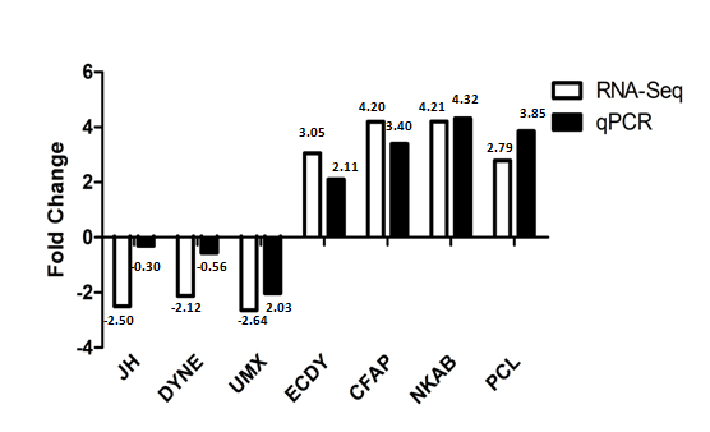


**Figure S6**

**Figure S6 7 DEGs identified with RNA-Seq were confirmed with qPCR.**


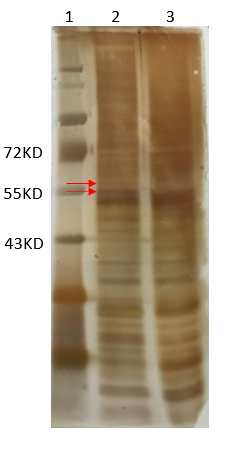


**Maker** **anti-BmYki** **con**

**Figure S7**

**72kDa**

**55kDa**

**43kDa**

**Figure S7 Co-immunoprecipitation**
