# Supplementary material for: Cultured cells and wing disc size of silkworm can be controlled by the Hippo pathway
Source: Open Biol. 2018 Jul 4;8(7):180029. doi: 10.1098/rsob.180029 (PMC6070717; doi:10.1098/rsob.180029)
Supplement: supplementary figures and Tables [file rsob180029supp1.zip › supplementary figures and tables/supplementary tables.docx]

**Cultured cells and wing disc size of silkworm can be controlled by Hippo pathway**

OPEN BIOLOGY. ID: RSOB-18-0029.R1

Zi Liang^a,c,1^Yahong Lu^a,c,1^, Ying Qian^a,c,1^, Liyuan Zhu^a,c^, Sulan Kuang^a,c^, Fei Chen^a,c^,Yongjie Feng^a,c^, Xiaolong Hu^a,b^, Guangli Cao^a,b^, Renyu Xue^a,b*^, Chengliang Gong^a,b,c*^

^a^School of Biology & Basic Medical Science, Soochow University, Suzhou 215123, China

^b^National Engineering Laboratory for Modern Silk, Soochow University, Suzhou, PR China

^C^Agricultural Biotechnology ResearchInstitute, Agricultural biotechnology and Ecological Research Institute, Soochow University, Suzhou, 215123, China

^1^These authors contributed equally to this work.

*Corresponding author: Tel: +86-512-65880183; Fax: +86-512-65880183.

E-mail address: xuery@suda.edu.cn, gongcl@suda.edu.cn

Table S1 The primers used in this study

| Primers | Sequences | GenBank accession No. | genes |
| --- | --- | --- | --- |
| Re-Hpo-1 | cccacttcgctggcctcact | NM_001123346.1 | *BmHpo* |
| Re-Hpo-2 | ccgctgttcttggagcactttga |  |  |
| Re-Sav-1 | ctcactaaatagcagcctccc | KF904337.1 | *BmSav* |
| Re-Sav-2 | cagcagcatcatatccagtaatc |  |  |
| Re-Wts2-1 | tagaaaggcggcggagaag | KF904338.1 | *BmWts* |
| Re-Wts2-2 | cgaccaccaatcgcaaagc |  |  |
| Re-Mats1-1 | aacactgtggatttcttcaatcaga | KF904334.1 | *BmMats1* |
| Re-Mats1-2 | aaataatctatatactttggggccg |  |  |
| Re-Yki1/3-1 | ggccacgacagcggaagg | KF904339.1(yki1), KF904341.1(yki3) | *BmYki1/3* |
| Re-Yki1/3-2 | tcgccaaagacgatacgatagaaga |  |  |
| Re-Yki1/2/3-1 | gcgtcttctatcgtatcgtctttggc | KF904340.1(yki2),KF904341.1(yki3) | *BmYki1/2/3* |
| Re-Yki1/2/3-2 | cgttgtcaggtttgctcggtgtt |  |  |
| Re-Yki3-2 | gttcagctgtaaagacggtac | KF904341.1(yki3) | *Bmyki3* |
| semi-A3-F | ctgcgtctggacttggc | NM_001126254.1 | *BmActin-3* |
| semi-A3-R | cgagggagctgctggat |  |  |
| Re-Iap-1 | ttgcaagacgagtgtcagtg | NM_001043559.1 | *BmIap* |
| Re-Iap-2 | caccaacgtggcggcagctcc |  |  |
| RE-Ex-1 | cgaggcatgaccgatacgga | XM_004929526.1 | *BmEx* |
| RE-Ex-2 | gcatctaatccatgagtgtg |  |  |
| RE-Kibra-1 | ccgcgtgacgtcacacctcg | XM_004922644.1 | *BmKibra* |
| RE-Kibra-2 | tcgggcaagggcatctcacc |  |  |
| Re-Crb-1 | gaacacgtgcattcaatgca | XM_004923952.1 | *BmCrb* |
| Re-Crb-2 | aggaccgctcatctcgcaag |  |  |
| Re-Fj-1 | gcggtgaaggtcctcgatga | XM_004925961.1 | *BmFj* |
| Re-Fj-2 | cggcctcgtgatgaggactcc |  |  |
| RE-Serr-1 | ctccatcatatggtagagtg | NM_001190840.1 | *BmSerr* |
| RE-Serr-2: | cgataggatatgtggtcagg |  |  |
| RE-Wnt-1 | cttgcacaacaacgaagctgg | NM_001043850.1 | *BmWnt* |
| RE-Wnt-2 | cctgaagctcggcagcctca |  |  |
| Re-Myc-1 | ccgaccgcatattctcagtgag | NM_001257008.1 | *BmMyc* |
| Re-Myc-2 | atgggttcaacgcacaccgtc |  |  |
| RE-Cat-1 | gtcagtggaacgcacactgg | XM_004924169.1 | *BmCat* |
| RE-Cat-2 | tctccgtggctcgttccacag |  |  |
| RE-Bmpr-1 | ctgttcctgctcgtagctgt | XM_004924066.1 | *BmBmpr* |
| RE-Bmpr-2 | gttgagacagcggcgacatg |  |  |
| RE-Dpp-1 | gcacgtaccgacggagctcg | NM_001145329.1 | *BmDpp* |
| RE-Dpp-2 | gagatcagcaccacgagctac |  |  |
| Re-CycE-1 | gacgttccatatctggtgcatg | AB457002.2 | *BmCycE* |
| Re-CycE-2 | ggagacccagattcagccata |  |  |
| Re-Stat-1 | ggagacggcaacgaagttttca | NM_001163916.1 | BmStat |
| Re-Stat-2 | ttgggaacgttcgggtacaa |  |  |
| Primers | Sequences | GenBank accession No. | genes |
| REJH-1 | aggagaccgggtattcgcatc | AK378544.1 | BmJH |
| REJH-2 | gtcaatgccgccttcatcttc |  |  |
| REDYNE-1 | tcgatcacagttaccggaag | XM_004921851.1 | BmDYNE |
| REDYNE-2 | cttcaagtttgtccagaagtccg |  |  |
| REUMX-1 | cagtcggtatctacgcacgtc | XM_004932559.1 | BmUMX |
| REUMX-2 | cgcagtttccacggcacacg |  |  |
| REECDY-1 | ggagactcgtggactaacaac | XM_004925371.1 | BmECDY |
| REECDY-2 | tggtcggtgcaattgaagatc |  |  |
| RECFAP-1 | caagtacggtccaattccagg | XM_004929546.1 | BmCFAP |
| RECFAP-2 | gtagtgacgttccggagtgtc |  |  |
| RENKAB-1 | aaggagcacatcaggaacatg | AK383274.1 | BmNKAB |
| RENKAB-2 | gcctcggtgaggtaggtactg |  |  |
| REPCL-1 | cacgaactggacgagtctgttg | XM_004923883.1 | BmPCL |
| REPCL-2 | cgatacgtagatatcgatcgag |  |  |
| Bmyki-1 | ggtaccatggctctcaactcggacgg | KF904339.1,KF904340.1,KF904341.1 | Bmyki |
| Bmyki-2 | gaattcttacagccacgtgagtacgttg |  |  |

Table S2 siRNA used in this study

| siRNAs | sense | antisense |
| --- | --- | --- |
| yki-siRNA-298 | 5’-GCAAGCUUGCAACAAACUUTT-3’ | 5’-AAGUUUGUUGCAAGCUUGCTT-3’ |
| yki-siRNA-608 | 5’-CCACACCUGCGCAACAUUUTT-3’ | 5’-AAAUGUUGCGCAGGUGUGGTT-3’ |
| yki-siRNA-928 | 5’-GCAGUCCCACAGUCCUAUUTT-3’ | 5’-AAUAGGACUGUGGGACUGCTT-3’ |
| siRNA-GFP-274 | 5’-GGCUACGUCCAGGAGCGCACC-3’ | 5’-UGCGCUCCUGGACGUAGCCUU-3’ |

Table 3 The enriched GO term for up-regulated DEGs

| GO | All_transcripts_with_GO_annotation(17360) | DETs_with_GO_annotation(6280) | Pvalue | Qvalue | GO_term | ratio |  |  |  |  |  |  |
| --- | --- | --- | --- | --- | --- | --- | --- | --- | --- | --- | --- | --- |
| GO:0006909 | 13 | 13 | 0 | 0 | phagocytosis | 1 |  |  |  |  |  |  |
| GO:0004467 | 12 | 12 | 0 | 0 | long-chain fatty acid-CoA ligase activity | 1 |  |  |  |  |  |  |
| GO:0005000 | 11 | 11 | 0 | 0 | vasopressin receptor activity | 1 |  |  |  |  |  |  |
| GO:0005550 | 11 | 11 | 0 | 0 | pheromone binding | 1 |  |  |  |  |  |  |
| GO:0016714 | 11 | 11 | 0 | 0 | oxidoreductase activity. acting on paired donors. with incorporation or reduction of molecular oxygen. reduced pteridine as one donor. and incorporation of one atom of oxygen | 1 |  |  |  |  |  |  |
| GO:0033280 | 11 | 11 | 0 | 0 | response to vitamin D | 1 |  |  |  |  |  |  |
| GO:0000082 | 10 | 10 | 0 | 0 | G1/S transition of mitotic cell cycle | 1 |  |  |  |  |  |  |
| GO:0001654 | 10 | 10 | 0 | 0 | eye development | 1 |  |  |  |  |  |  |
| GO:0000228 | 9 | 9 | 0 | 0 | nuclear chromosome | 1 |  |  |  |  |  |  |
| GO:0004884 | 9 | 9 | 0 | 0 | ecdysteroid hormone receptor activity | 1 |  |  |  |  |  |  |
| GO:0035076 | 9 | 9 | 0 | 0 | ecdysone receptor-mediated signaling pathway | 1 |  |  |  |  |  |  |
| GO:0045335 | 9 | 9 | 0 | 0 | phagocytic vesicle | 1 |  |  |  |  |  |  |
| GO:0000149 | 8 | 8 | 0 | 0 | SNARE binding | 1 |  |  |  |  |  |  |
| GO:0000790 | 8 | 8 | 0 | 0 | nuclear chromatin | 1 |  |  |  |  |  |  |
| GO:0000902 | 8 | 8 | 0 | 0 | cell morphogenesis | 1 |  |  |  |  |  |  |
| GO:0004368 | 8 | 8 | 0 | 0 | glycerol-3-phosphate dehydrogenase activity | 1 |  |  |  |  |  |  |
| GO:0007269 | 8 | 8 | 0 | 0 | neurotransmitter secretion | 1 |  |  |  |  |  |  |
| GO:0046274 | 8 | 8 | 0 | 0 | lignin catabolic process | 1 |  |  |  |  |  |  |
| GO:0001518 | 7 | 7 | 0 | 0 | voltage-gated sodium channel complex | 1 |  |  |  |  |  |  |
| GO:0004085 | 7 | 7 | 0 | 0 | butyryl-CoA dehydrogenase activity | 1 |  |  |  |  |  |  |
| GO:0005200 | 7 | 7 | 0 | 0 | structural constituent of cytoskeleton | 1 |  |  |  |  |  |  |
| GO:0005248 | 7 | 7 | 0 | 0 | voltage-gated sodium channel activity | 1 |  |  |  |  |  |  |
| GO:0005942 | 7 | 7 | 0 | 0 | phosphatidylinositol 3-kinase complex | 1 |  |  |  |  |  |  |
| GO:0008286 | 7 | 7 | 0 | 0 | insulin receptor signaling pathway | 1 |  |  |  |  |  |  |
| GO:0052590 | 7 | 7 | 0 | 0 | sn-glycerol-3-phosphate:ubiquinone oxidoreductase activity | 1 |  |  |  |  |  |  |
| GO:0052591 | 7 | 7 | 0 | 0 | sn-glycerol-3-phosphate:ubiquinone-8 oxidoreductase activity | 1 |  |  |  |  |  |  |
| GO:0055003 | 7 | 7 | 0 | 0 | cardiac myofibril assembly | 1 |  |  |  |  |  |  |
| GO:0003785 | 6 | 6 | 0 | 0 | actin monomer binding | 1 |  |  |  |  |  |  |
| GO:0004428 | 6 | 6 | 0 | 0 | inositol or phosphatidylinositol kinase activity | 1 |  |  |  |  |  |  |
| GO:0004459 | 6 | 6 | 0 | 0 | L-lactate dehydrogenase activity | 1 |  |  |  |  |  |  |
| GO:0004645 | 6 | 6 | 0 | 0 | phosphorylase activity | 1 |  |  |  |  |  |  |
| GO:0009279 | 6 | 6 | 0 | 0 | cell outer membrane | 1 |  |  |  |  |  |  |
| GO:0014070 | 6 | 6 | 0 | 0 | response to organic cyclic compound | 1 |  |  |  |  |  |  |
| GO:0019905 | 6 | 6 | 0 | 0 | syntaxin binding | 1 |  |  |  |  |  |  |
| GO:0031116 | 6 | 6 | 0 | 0 | positive regulation of microtubule polymerization | 1 |  |  |  |  |  |  |
| GO:0042694 | 6 | 6 | 0 | 0 | muscle cell fate specification | 1 |  |  |  |  |  |  |
| GO:0043560 | 6 | 6 | 0 | 0 | insulin receptor substrate binding | 1 |  |  |  |  |  |  |
| GO:0045197 | 6 | 6 | 0 | 0 | establishment or maintenance of epithelial cell apical/basal polarity | 1 |  |  |  |  |  |  |
| GO:0047886 | 6 | 6 | 0 | 0 | farnesol dehydrogenase activity | 1 |  |  |  |  |  |  |
| GO:0048015 | 6 | 6 | 0 | 0 | phosphatidylinositol-mediated signaling | 1 |  |  |  |  |  |  |
| GO:0055010 | 6 | 6 | 0 | 0 | ventricular cardiac muscle tissue morphogenesis | 1 |  |  |  |  |  |  |
| GO:0001741 | 5 | 5 | 0 | 0 | XY body | 1 |  |  |  |  |  |  |
| GO:0003918 | 5 | 5 | 0 | 0 | DNA topoisomerase (ATP-hydrolyzing) activity | 1 |  |  |  |  |  |  |
| GO:0003934 | 5 | 5 | 0 | 0 | GTP cyclohydrolase I activity | 1 |  |  |  |  |  |  |
| GO:0003983 | 5 | 5 | 0 | 0 | UTP:glucose-1-phosphate uridylyltransferase activity | 1 |  |  |  |  |  |  |
| GO:0004064 | 5 | 5 | 0 | 0 | arylesterase activity | 1 |  |  |  |  |  |  |
| GO:0004505 | 5 | 5 | 0 | 0 | phenylalanine 4-monooxygenase activity | 1 |  |  |  |  |  |  |
| GO:0004511 | 5 | 5 | 0 | 0 | tyrosine 3-monooxygenase activity | 1 |  |  |  |  |  |  |
| GO:0004514 | 5 | 5 | 0 | 0 | nicotinate-nucleotide diphosphorylase (carboxylating) activity | 1 |  |  |  |  |  |  |
| GO:0004516 | 5 | 5 | 0 | 0 | nicotinate phosphoribosyltransferase activity | 1 |  |  |  |  |  |  |
| GO:0004948 | 5 | 5 | 0 | 0 | calcitonin receptor activity | 1 |  |  |  |  |  |  |
| GO:0005657 | 5 | 5 | 0 | 0 | replication fork | 1 |  |  |  |  |  |  |
| GO:0005956 | 5 | 5 | 0 | 0 | protein kinase CK2 complex | 1 |  |  |  |  |  |  |
| GO:0006344 | 5 | 5 | 0 | 0 | maintenance of chromatin silencing | 1 |  |  |  |  |  |  |
| GO:0006750 | 5 | 5 | 0 | 0 | glutathione biosynthetic process | 1 |  |  |  |  |  |  |
| GO:0007288 | 5 | 5 | 0 | 0 | sperm axoneme assembly | 1 |  |  |  |  |  |  |
| GO:0008360 | 5 | 5 | 0 | 0 | regulation of cell shape | 1 |  |  |  |  |  |  |
| GO:0010845 | 5 | 5 | 0 | 0 | positive regulation of reciprocal meiotic recombination | 1 |  |  |  |  |  |  |
| GO:0010977 | 5 | 5 | 0 | 0 | negative regulation of neuron projection development | 1 |  |  |  |  |  |  |
| GO:0016234 | 5 | 5 | 0 | 0 | inclusion body | 1 |  |  |  |  |  |  |
| GO:0017048 | 5 | 5 | 0 | 0 | Rho GTPase binding | 1 |  |  |  |  |  |  |
| GO:0019226 | 5 | 5 | 0 | 0 | transmission of nerve impulse | 1 |  |  |  |  |  |  |
| GO:0019358 | 5 | 5 | 0 | 0 | nicotinate nucleotide salvage | 1 |  |  |  |  |  |  |
| GO:0019887 | 5 | 5 | 0 | 0 | protein kinase regulator activity | 1 |  |  |  |  |  |  |
| GO:0030272 | 5 | 5 | 0 | 0 | 5-formyltetrahydrofolate cyclo-ligase activity | 1 |  |  |  |  |  |  |
| GO:0030521 | 5 | 5 | 0 | 0 | androgen receptor signaling pathway | 1 |  |  |  |  |  |  |
| GO:0030539 | 5 | 5 | 0 | 0 | male genitalia development | 1 |  |  |  |  |  |  |
| GO:0031111 | 5 | 5 | 0 | 0 | negative regulation of microtubule polymerization or depolymerization | 1 |  |  |  |  |  |  |
| GO:0031143 | 5 | 5 | 0 | 0 | pseudopodium | 1 |  |  |  |  |  |  |
| GO:0032579 | 5 | 5 | 0 | 0 | apical lamina of hyaline layer | 1 |  |  |  |  |  |  |
| GO:0033128 | 5 | 5 | 0 | 0 | negative regulation of histone phosphorylation | 1 |  |  |  |  |  |  |
| GO:0033211 | 5 | 5 | 0 | 0 | adiponectin-mediated signaling pathway | 1 |  |  |  |  |  |  |
| GO:0033522 | 5 | 5 | 0 | 0 | histone H2A ubiquitination | 1 |  |  |  |  |  |  |
| GO:0043951 | 5 | 5 | 0 | 0 | negative regulation of cAMP-mediated signaling | 1 |  |  |  |  |  |  |
| GO:0044295 | 5 | 5 | 0 | 0 | axonal growth cone | 1 |  |  |  |  |  |  |
| GO:0046654 | 5 | 5 | 0 | 0 | tetrahydrofolate biosynthetic process | 1 |  |  |  |  |  |  |
| GO:0046912 | 5 | 5 | 0 | 0 | transferase activity. transferring acyl groups. acyl groups converted into alkyl on transfer | 1 |  |  |  |  |  |  |
| GO:0048156 | 5 | 5 | 0 | 0 | tau protein binding | 1 |  |  |  |  |  |  |
| GO:0051026 | 5 | 5 | 0 | 0 | chiasma assembly | 1 |  |  |  |  |  |  |
| GO:0070076 | 5 | 5 | 0 | 0 | histone lysine demethylation | 1 |  |  |  |  |  |  |
| GO:0070193 | 5 | 5 | 0 | 0 | synaptonemal complex organization | 1 |  |  |  |  |  |  |
| GO:0000832 | 4 | 4 | 0 | 0 | inositol hexakisphosphate 5-kinase activity | 1 |  |  |  |  |  |  |
| GO:0001671 | 4 | 4 | 0 | 0 | ATPase activator activity | 1 |  |  |  |  |  |  |
| GO:0003960 | 4 | 4 | 0 | 0 | NADPH:quinone reductase activity | 1 |  |  |  |  |  |  |
| GO:0004100 | 4 | 4 | 0 | 0 | chitin synthase activity | 1 |  |  |  |  |  |  |
| GO:0004144 | 4 | 4 | 0 | 0 | diacylglycerol O-acyltransferase activity | 1 |  |  |  |  |  |  |
| GO:0004385 | 4 | 4 | 0 | 0 | guanylate kinase activity | 1 |  |  |  |  |  |  |
| GO:0004439 | 4 | 4 | 0 | 0 | phosphatidylinositol-4.5-bisphosphate 5-phosphatase activity | 1 |  |  |  |  |  |  |
| GO:0004476 | 4 | 4 | 0 | 0 | mannose-6-phosphate isomerase activity | 1 |  |  |  |  |  |  |
| GO:0004496 | 4 | 4 | 0 | 0 | mevalonate kinase activity | 1 |  |  |  |  |  |  |
| GO:0004615 | 4 | 4 | 0 | 0 | phosphomannomutase activity | 1 |  |  |  |  |  |  |
| GO:0004617 | 4 | 4 | 0 | 0 | phosphoglycerate dehydrogenase activity | 1 |  |  |  |  |  |  |
| GO:0006105 | 4 | 4 | 0 | 0 | succinate metabolic process | 1 |  |  |  |  |  |  |
| GO:0006163 | 4 | 4 | 0 | 0 | purine nucleotide metabolic process | 1 |  |  |  |  |  |  |
| GO:0007090 | 4 | 4 | 0 | 0 | regulation of S phase of mitotic cell cycle | 1 |  |  |  |  |  |  |
| GO:0007306 | 4 | 4 | 0 | 0 | eggshell chorion assembly | 1 |  |  |  |  |  |  |
| GO:0008195 | 4 | 4 | 0 | 0 | phosphatidate phosphatase activity | 1 |  |  |  |  |  |  |
| GO:0009950 | 4 | 4 | 0 | 0 | dorsal/ventral axis specification | 1 |  |  |  |  |  |  |
| GO:0010975 | 4 | 4 | 0 | 0 | regulation of neuron projection development | 1 |  |  |  |  |  |  |
| GO:0012506 | 4 | 4 | 0 | 0 | vesicle membrane | 1 |  |  |  |  |  |  |
| GO:0015018 | 4 | 4 | 0 | 0 | galactosylgalactosylxylosylprotein 3-beta-glucuronosyltransferase activity | 1 |  |  |  |  |  |  |
| GO:0016079 | 4 | 4 | 0 | 0 | synaptic vesicle exocytosis | 1 |  |  |  |  |  |  |
| GO:0016499 | 4 | 4 | 0 | 0 | orexin receptor activity | 1 |  |  |  |  |  |  |
| GO:0016500 | 4 | 4 | 0 | 0 | protein-hormone receptor activity | 1 |  |  |  |  |  |  |
| GO:0017157 | 4 | 4 | 0 | 0 | regulation of exocytosis | 1 |  |  |  |  |  |  |
| GO:0019212 | 4 | 4 | 0 | 0 | phosphatase inhibitor activity | 1 |  |  |  |  |  |  |
| GO:0019307 | 4 | 4 | 0 | 0 | mannose biosynthetic process | 1 |  |  |  |  |  |  |
| GO:0030165 | 4 | 4 | 0 | 0 | PDZ domain binding | 1 |  |  |  |  |  |  |
| GO:0030540 | 4 | 4 | 0 | 0 | female genitalia development | 1 |  |  |  |  |  |  |
| GO:0031054 | 4 | 4 | 0 | 0 | pre-miRNA processing | 1 |  |  |  |  |  |  |
| GO:0031435 | 4 | 4 | 0 | 0 | mitogen-activated protein kinase kinase kinase binding | 1 |  |  |  |  |  |  |
| GO:0031929 | 4 | 4 | 0 | 0 | TOR signaling cascade | 1 |  |  |  |  |  |  |
| GO:0033018 | 4 | 4 | 0 | 0 | sarcoplasmic reticulum lumen | 1 |  |  |  |  |  |  |
| GO:0035014 | 4 | 4 | 0 | 0 | phosphatidylinositol 3-kinase regulator activity | 1 |  |  |  |  |  |  |
| GO:0035282 | 4 | 4 | 0 | 0 | segmentation | 1 |  |  |  |  |  |  |
| GO:0042169 | 4 | 4 | 0 | 0 | SH2 domain binding | 1 |  |  |  |  |  |  |
| GO:0042743 | 4 | 4 | 0 | 0 | hydrogen peroxide metabolic process | 1 |  |  |  |  |  |  |
| GO:0043278 | 4 | 4 | 0 | 0 | response to morphine | 1 |  |  |  |  |  |  |
| GO:0043407 | 4 | 4 | 0 | 0 | negative regulation of MAP kinase activity | 1 |  |  |  |  |  |  |
| GO:0043548 | 4 | 4 | 0 | 0 | phosphatidylinositol 3-kinase binding | 1 |  |  |  |  |  |  |
| GO:0045177 | 4 | 4 | 0 | 0 | apical part of cell | 1 |  |  |  |  |  |  |
| GO:0045496 | 4 | 4 | 0 | 0 | male analia development | 1 |  |  |  |  |  |  |
| GO:0045497 | 4 | 4 | 0 | 0 | female analia development | 1 |  |  |  |  |  |  |
| GO:0045948 | 4 | 4 | 0 | 0 | positive regulation of translational initiation | 1 |  |  |  |  |  |  |
| GO:0046320 | 4 | 4 | 0 | 0 | regulation of fatty acid oxidation | 1 |  |  |  |  |  |  |
| GO:0046330 | 4 | 4 | 0 | 0 | positive regulation of JNK cascade | 1 |  |  |  |  |  |  |
| GO:0046426 | 4 | 4 | 0 | 0 | negative regulation of JAK-STAT cascade | 1 |  |  |  |  |  |  |
| GO:0050254 | 4 | 4 | 0 | 0 | rhodopsin kinase activity | 1 |  |  |  |  |  |  |
| GO:0050773 | 4 | 4 | 0 | 0 | regulation of dendrite development | 1 |  |  |  |  |  |  |
| GO:0060171 | 4 | 4 | 0 | 0 | stereocilium membrane | 1 |  |  |  |  |  |  |
| GO:0070032 | 4 | 4 | 0 | 0 | synaptobrevin 2-SNAP-25-syntaxin-1a-complexin I complex | 1 |  |  |  |  |  |  |
| GO:0071383 | 4 | 4 | 0 | 0 | cellular response to steroid hormone stimulus | 1 |  |  |  |  |  |  |
| GO:0071385 | 4 | 4 | 0 | 0 | cellular response to glucocorticoid stimulus | 1 |  |  |  |  |  |  |
| GO:0046677 | 13 | 12 | 1.80E-06 | 2.27E-05 | response to antibiotic | 0.923077 |  |  |  |  |  |  |
| GO:0006013 | 12 | 11 | 4.99E-06 | 5.96E-05 | mannose metabolic process | 0.916667 |  |  |  |  |  |  |
| GO:0007422 | 12 | 11 | 4.99E-06 | 5.96E-05 | peripheral nervous system development | 0.916667 |  |  |  |  |  |  |
| GO:0000209 | 10 | 9 | 3.82E-05 | 0.000387 | protein polyubiquitination | 0.9 |  |  |  |  |  |  |
| GO:0004054 | 10 | 9 | 3.82E-05 | 0.000387 | arginine kinase activity | 0.9 |  |  |  |  |  |  |
| GO:0000272 | 27 | 24 | 1.33E-09 | 1.88E-08 | polysaccharide catabolic process | 0.888889 |  |  |  |  |  |  |
| GO:0004559 | 9 | 8 | 0.000106 | 0.000994 | alpha-mannosidase activity | 0.888889 |  |  |  |  |  |  |
| GO:0015923 | 9 | 8 | 0.000106 | 0.000994 | mannosidase activity | 0.888889 |  |  |  |  |  |  |
| GO:0016891 | 9 | 8 | 0.000106 | 0.000994 | endoribonuclease activity. producing 5'-phosphomonoesters | 0.888889 |  |  |  |  |  |  |
| GO:0004563 | 24 | 21 | 2.23E-08 | 3.04E-07 | beta-N-acetylhexosaminidase activity | 0.875 |  |  |  |  |  |  |
| GO:0005351 | 8 | 7 | 0.000292 | 0.002507 | sugar:hydrogen symporter activity | 0.875 |  |  |  |  |  |  |
| GO:0007032 | 8 | 7 | 0.000292 | 0.002507 | endosome organization | 0.875 |  |  |  |  |  |  |
| GO:0009435 | 8 | 7 | 0.000292 | 0.002507 | NAD biosynthetic process | 0.875 |  |  |  |  |  |  |
| GO:0034220 | 8 | 7 | 0.000292 | 0.002507 | ion transmembrane transport | 0.875 |  |  |  |  |  |  |
| GO:0001607 | 15 | 13 | 6.47E-06 | 7.36E-05 | neuromedin U receptor activity | 0.866667 |  |  |  |  |  |  |
| GO:0004161 | 15 | 13 | 6.47E-06 | 7.36E-05 | dimethylallyltranstransferase activity | 0.866667 |  |  |  |  |  |  |
| GO:0004337 | 15 | 13 | 6.47E-06 | 7.36E-05 | geranyltranstransferase activity | 0.866667 |  |  |  |  |  |  |
| GO:0030139 | 14 | 12 | 1.68E-05 | 0.000181 | endocytic vesicle | 0.857143 |  |  |  |  |  |  |
| GO:0004555 | 7 | 6 | 0.000809 | 0.006121 | alpha.alpha-trehalase activity | 0.857143 |  |  |  |  |  |  |
| GO:0005991 | 7 | 6 | 0.000809 | 0.006121 | trehalose metabolic process | 0.857143 |  |  |  |  |  |  |
| GO:0006452 | 7 | 6 | 0.000809 | 0.006121 | translational frameshifting | 0.857143 |  |  |  |  |  |  |
| GO:0008374 | 7 | 6 | 0.000809 | 0.006121 | O-acyltransferase activity | 0.857143 |  |  |  |  |  |  |
| GO:0016585 | 7 | 6 | 0.000809 | 0.006121 | chromatin remodeling complex | 0.857143 |  |  |  |  |  |  |
| GO:0030071 | 7 | 6 | 0.000809 | 0.006121 | regulation of mitotic metaphase/anaphase transition | 0.857143 |  |  |  |  |  |  |
| GO:0030100 | 7 | 6 | 0.000809 | 0.006121 | regulation of endocytosis | 0.857143 |  |  |  |  |  |  |
| GO:0030742 | 7 | 6 | 0.000809 | 0.006121 | GTP-dependent protein binding | 0.857143 |  |  |  |  |  |  |
| GO:0045901 | 7 | 6 | 0.000809 | 0.006121 | positive regulation of translational elongation | 0.857143 |  |  |  |  |  |  |
| GO:0045905 | 7 | 6 | 0.000809 | 0.006121 | positive regulation of translational termination | 0.857143 |  |  |  |  |  |  |
| GO:0046922 | 7 | 6 | 0.000809 | 0.006121 | peptide-O-fucosyltransferase activity | 0.857143 |  |  |  |  |  |  |
| GO:0004035 | 13 | 11 | 4.32E-05 | 0.000425 | alkaline phosphatase activity | 0.846154 |  |  |  |  |  |  |
| GO:0005272 | 13 | 11 | 4.32E-05 | 0.000425 | sodium channel activity | 0.846154 |  |  |  |  |  |  |
| GO:0035725 | 13 | 11 | 4.32E-05 | 0.000425 | sodium ion transmembrane transport | 0.846154 |  |  |  |  |  |  |
| GO:0007243 | 18 | 15 | 5.67E-06 | 6.63E-05 | intracellular protein kinase cascade | 0.833333 |  |  |  |  |  |  |
| GO:0010389 | 12 | 10 | 0.000111 | 0.001038 | regulation of G2/M transition of mitotic cell cycle | 0.833333 |  |  |  |  |  |  |
| GO:0007605 | 6 | 5 | 0.002238 | 0.015103 | sensory perception of sound | 0.833333 |  |  |  |  |  |  |
| GO:0008158 | 6 | 5 | 0.002238 | 0.015103 | hedgehog receptor activity | 0.833333 |  |  |  |  |  |  |
| GO:0008543 | 6 | 5 | 0.002238 | 0.015103 | fibroblast growth factor receptor signaling pathway | 0.833333 |  |  |  |  |  |  |
| GO:0015075 | 6 | 5 | 0.002238 | 0.015103 | ion transmembrane transporter activity | 0.833333 |  |  |  |  |  |  |
| GO:0016337 | 6 | 5 | 0.002238 | 0.015103 | cell-cell adhesion | 0.833333 |  |  |  |  |  |  |
| GO:0016812 | 6 | 5 | 0.002238 | 0.015103 | hydrolase activity. acting on carbon-nitrogen (but not peptide) bonds. in cyclic amides | 0.833333 |  |  |  |  |  |  |
| GO:0030900 | 6 | 5 | 0.002238 | 0.015103 | forebrain development | 0.833333 |  |  |  |  |  |  |
| GO:0004453 | 32 | 26 | 2.75E-08 | 3.70E-07 | juvenile-hormone esterase activity | 0.8125 |  |  |  |  |  |  |
| GO:0005625 | 16 | 13 | 3.44E-05 | 0.000355 | soluble fraction | 0.8125 |  |  |  |  |  |  |
| GO:0005912 | 15 | 12 | 8.37E-05 | 0.000805 | adherens junction | 0.8 |  |  |  |  |  |  |
| GO:0005388 | 10 | 8 | 0.000713 | 0.005637 | calcium-transporting ATPase activity | 0.8 |  |  |  |  |  |  |
| GO:0007050 | 10 | 8 | 0.000713 | 0.005637 | cell cycle arrest | 0.8 |  |  |  |  |  |  |
| GO:0015077 | 10 | 8 | 0.000713 | 0.005637 | monovalent inorganic cation transmembrane transporter activity | 0.8 |  |  |  |  |  |  |
| GO:0003958 | 5 | 4 | 0.006189 | 0.035561 | NADPH-hemoprotein reductase activity | 0.8 |  |  |  |  |  |  |
| GO:0004012 | 5 | 4 | 0.006189 | 0.035561 | phospholipid-translocating ATPase activity | 0.8 |  |  |  |  |  |  |
| GO:0004311 | 5 | 4 | 0.006189 | 0.035561 | farnesyltranstransferase activity | 0.8 |  |  |  |  |  |  |
| GO:0004396 | 5 | 4 | 0.006189 | 0.035561 | hexokinase activity | 0.8 |  |  |  |  |  |  |
| GO:0004692 | 5 | 4 | 0.006189 | 0.035561 | cGMP-dependent protein kinase activity | 0.8 |  |  |  |  |  |  |
| GO:0004791 | 5 | 4 | 0.006189 | 0.035561 | thioredoxin-disulfide reductase activity | 0.8 |  |  |  |  |  |  |
| GO:0004864 | 5 | 4 | 0.006189 | 0.035561 | protein phosphatase inhibitor activity | 0.8 |  |  |  |  |  |  |
| GO:0004935 | 5 | 4 | 0.006189 | 0.035561 | adrenergic receptor activity | 0.8 |  |  |  |  |  |  |
| GO:0005859 | 5 | 4 | 0.006189 | 0.035561 | muscle myosin complex | 0.8 |  |  |  |  |  |  |
| GO:0005871 | 5 | 4 | 0.006189 | 0.035561 | kinesin complex | 0.8 |  |  |  |  |  |  |
| GO:0006720 | 5 | 4 | 0.006189 | 0.035561 | isoprenoid metabolic process | 0.8 |  |  |  |  |  |  |
| GO:0007213 | 5 | 4 | 0.006189 | 0.035561 | G-protein coupled acetylcholine receptor signaling pathway | 0.8 |  |  |  |  |  |  |
| GO:0015914 | 5 | 4 | 0.006189 | 0.035561 | phospholipid transport | 0.8 |  |  |  |  |  |  |
| GO:0016639 | 5 | 4 | 0.006189 | 0.035561 | oxidoreductase activity. acting on the CH-NH2 group of donors. NAD or NADP as acceptor | 0.8 |  |  |  |  |  |  |
| GO:0017040 | 5 | 4 | 0.006189 | 0.035561 | ceramidase activity | 0.8 |  |  |  |  |  |  |
| GO:0030048 | 5 | 4 | 0.006189 | 0.035561 | actin filament-based movement | 0.8 |  |  |  |  |  |  |
| GO:0030553 | 5 | 4 | 0.006189 | 0.035561 | cGMP binding | 0.8 |  |  |  |  |  |  |
| GO:0030898 | 5 | 4 | 0.006189 | 0.035561 | actin-dependent ATPase activity | 0.8 |  |  |  |  |  |  |
| GO:0043197 | 5 | 4 | 0.006189 | 0.035561 | dendritic spine | 0.8 |  |  |  |  |  |  |
| GO:0047131 | 5 | 4 | 0.006189 | 0.035561 | saccharopine dehydrogenase (NAD+. L-glutamate-forming) activity | 0.8 |  |  |  |  |  |  |
| GO:0051091 | 5 | 4 | 0.006189 | 0.035561 | positive regulation of sequence-specific DNA binding transcription factor activity | 0.8 |  |  |  |  |  |  |
| GO:0071875 | 5 | 4 | 0.006189 | 0.035561 | adrenergic receptor signaling pathway | 0.8 |  |  |  |  |  |  |
| GO:0071407 | 14 | 11 | 0.000202 | 0.001789 | cellular response to organic cyclic compound | 0.785714 |  |  |  |  |  |  |
| GO:0005978 | 9 | 7 | 0.001786 | 0.012664 | glycogen biosynthetic process | 0.777778 |  |  |  |  |  |  |
| GO:0009536 | 9 | 7 | 0.001786 | 0.012664 | plastid | 0.777778 |  |  |  |  |  |  |
| GO:0016765 | 9 | 7 | 0.001786 | 0.012664 | transferase activity. transferring alkyl or aryl (other than methyl) groups | 0.777778 |  |  |  |  |  |  |
| GO:0051056 | 26 | 20 | 4.17E-06 | 5.10E-05 | regulation of small GTPase mediated signal transduction | 0.769231 |  |  |  |  |  |  |
| GO:0051865 | 13 | 10 | 0.000482 | 0.00393 | protein autoubiquitination | 0.769231 |  |  |  |  |  |  |
| GO:0004768 | 47 | 36 | 2.97E-09 | 4.13E-08 | stearoyl-CoA 9-desaturase activity | 0.765957 |  |  |  |  |  |  |
| GO:0015662 | 28 | 21 | 5.63E-06 | 6.63E-05 | ATPase activity. coupled to transmembrane movement of ions. phosphorylative mechanism | 0.75 |  |  |  |  |  |  |
| GO:0031012 | 28 | 21 | 5.63E-06 | 6.63E-05 | extracellular matrix | 0.75 |  |  |  |  |  |  |
| GO:0006644 | 20 | 15 | 7.86E-05 | 0.000759 | phospholipid metabolic process | 0.75 |  |  |  |  |  |  |
| GO:0008378 | 16 | 12 | 0.000297 | 0.002539 | galactosyltransferase activity | 0.75 |  |  |  |  |  |  |
| GO:0046854 | 12 | 9 | 0.001139 | 0.00838 | phosphatidylinositol phosphorylation | 0.75 |  |  |  |  |  |  |
| GO:0003916 | 8 | 6 | 0.004425 | 0.027587 | DNA topoisomerase activity | 0.75 |  |  |  |  |  |  |
| GO:0006535 | 8 | 6 | 0.004425 | 0.027587 | cysteine biosynthetic process from serine | 0.75 |  |  |  |  |  |  |
| GO:0006725 | 8 | 6 | 0.004425 | 0.027587 | cellular aromatic compound metabolic process | 0.75 |  |  |  |  |  |  |
| GO:0008271 | 8 | 6 | 0.004425 | 0.027587 | secondary active sulfate transmembrane transporter activity | 0.75 |  |  |  |  |  |  |
| GO:0008272 | 8 | 6 | 0.004425 | 0.027587 | sulfate transport | 0.75 |  |  |  |  |  |  |
| GO:0051015 | 8 | 6 | 0.004425 | 0.027587 | actin filament binding | 0.75 |  |  |  |  |  |  |
| GO:0000145 | 4 | 3 | 0.017115 | 0.079446 | exocyst | 0.75 |  |  |  |  |  |  |
| GO:0001541 | 4 | 3 | 0.017115 | 0.079446 | ovarian follicle development | 0.75 |  |  |  |  |  |  |
| GO:0001756 | 4 | 3 | 0.017115 | 0.079446 | somitogenesis | 0.75 |  |  |  |  |  |  |
| GO:0003705 | 4 | 3 | 0.017115 | 0.079446 | RNA polymerase II distal enhancer sequence-specific DNA binding transcription factor activity | 0.75 |  |  |  |  |  |  |
| GO:0004335 | 4 | 3 | 0.017115 | 0.079446 | galactokinase activity | 0.75 |  |  |  |  |  |  |
| GO:0004342 | 4 | 3 | 0.017115 | 0.079446 | glucosamine-6-phosphate deaminase activity | 0.75 |  |  |  |  |  |  |
| GO:0004564 | 4 | 3 | 0.017115 | 0.079446 | beta-fructofuranosidase activity | 0.75 |  |  |  |  |  |  |
| GO:0004575 | 4 | 3 | 0.017115 | 0.079446 | sucrose alpha-glucosidase activity | 0.75 |  |  |  |  |  |  |
| GO:0005057 | 4 | 3 | 0.017115 | 0.079446 | receptor signaling protein activity | 0.75 |  |  |  |  |  |  |
| GO:0005337 | 4 | 3 | 0.017115 | 0.079446 | nucleoside transmembrane transporter activity | 0.75 |  |  |  |  |  |  |
| GO:0005338 | 4 | 3 | 0.017115 | 0.079446 | nucleotide-sugar transmembrane transporter activity | 0.75 |  |  |  |  |  |  |
| GO:0006043 | 4 | 3 | 0.017115 | 0.079446 | glucosamine catabolic process | 0.75 |  |  |  |  |  |  |
| GO:0006166 | 4 | 3 | 0.017115 | 0.079446 | purine ribonucleoside salvage | 0.75 |  |  |  |  |  |  |
| GO:0006537 | 4 | 3 | 0.017115 | 0.079446 | glutamate biosynthetic process | 0.75 |  |  |  |  |  |  |
| GO:0008107 | 4 | 3 | 0.017115 | 0.079446 | galactoside 2-alpha-L-fucosyltransferase activity | 0.75 |  |  |  |  |  |  |
| GO:0008295 | 4 | 3 | 0.017115 | 0.079446 | spermidine biosynthetic process | 0.75 |  |  |  |  |  |  |
| GO:0008407 | 4 | 3 | 0.017115 | 0.079446 | chaeta morphogenesis | 0.75 |  |  |  |  |  |  |
| GO:0015175 | 4 | 3 | 0.017115 | 0.079446 | neutral amino acid transmembrane transporter activity | 0.75 |  |  |  |  |  |  |
| GO:0015780 | 4 | 3 | 0.017115 | 0.079446 | nucleotide-sugar transport | 0.75 |  |  |  |  |  |  |
| GO:0015858 | 4 | 3 | 0.017115 | 0.079446 | nucleoside transport | 0.75 |  |  |  |  |  |  |
| GO:0016197 | 4 | 3 | 0.017115 | 0.079446 | endosomal transport | 0.75 |  |  |  |  |  |  |
| GO:0016298 | 4 | 3 | 0.017115 | 0.079446 | lipase activity | 0.75 |  |  |  |  |  |  |
| GO:0030674 | 4 | 3 | 0.017115 | 0.079446 | protein binding. bridging | 0.75 |  |  |  |  |  |  |
| GO:0030855 | 4 | 3 | 0.017115 | 0.079446 | epithelial cell differentiation | 0.75 |  |  |  |  |  |  |
| GO:0031258 | 4 | 3 | 0.017115 | 0.079446 | lamellipodium membrane | 0.75 |  |  |  |  |  |  |
| GO:0032027 | 4 | 3 | 0.017115 | 0.079446 | myosin light chain binding | 0.75 |  |  |  |  |  |  |
| GO:0032266 | 4 | 3 | 0.017115 | 0.079446 | phosphatidylinositol-3-phosphate binding | 0.75 |  |  |  |  |  |  |
| GO:0035317 | 4 | 3 | 0.017115 | 0.079446 | imaginal disc-derived wing hair organization | 0.75 |  |  |  |  |  |  |
| GO:0044403 | 4 | 3 | 0.017115 | 0.079446 | symbiosis. encompassing mutualism through parasitism | 0.75 |  |  |  |  |  |  |
| GO:0046835 | 4 | 3 | 0.017115 | 0.079446 | carbohydrate phosphorylation | 0.75 |  |  |  |  |  |  |
| GO:0048800 | 4 | 3 | 0.017115 | 0.079446 | antennal morphogenesis | 0.75 |  |  |  |  |  |  |
| GO:0090090 | 4 | 3 | 0.017115 | 0.079446 | negative regulation of canonical Wnt receptor signaling pathway | 0.75 |  |  |  |  |  |  |
| GO:0000060 | 19 | 14 | 0.000176 | 0.001588 | protein import into nucleus. translocation | 0.736842 |  |  |  |  |  |  |
| GO:0007156 | 30 | 22 | 7.16E-06 | 8.02E-05 | homophilic cell adhesion | 0.733333 |  |  |  |  |  |  |
| GO:0003777 | 41 | 30 | 3.01E-07 | 3.88E-06 | microtubule motor activity | 0.731707 |  |  |  |  |  |  |
| GO:0042381 | 26 | 19 | 2.68E-05 | 0.000284 | hemolymph coagulation | 0.730769 |  |  |  |  |  |  |
| GO:0004047 | 11 | 8 | 0.002653 | 0.017625 | aminomethyltransferase activity | 0.727273 |  |  |  |  |  |  |
| GO:0005096 | 53 | 38 | 3.17E-08 | 4.22E-07 | GTPase activator activity | 0.716981 |  |  |  |  |  |  |
| GO:0043547 | 53 | 38 | 3.17E-08 | 4.22E-07 | positive regulation of GTPase activity | 0.716981 |  |  |  |  |  |  |
| GO:0009072 | 28 | 20 | 3.25E-05 | 0.000337 | aromatic amino acid family metabolic process | 0.714286 |  |  |  |  |  |  |
| GO:0031224 | 28 | 20 | 3.25E-05 | 0.000337 | intrinsic to membrane | 0.714286 |  |  |  |  |  |  |
| GO:0004850 | 7 | 5 | 0.01081 | 0.057598 | uridine phosphorylase activity | 0.714286 |  |  |  |  |  |  |
| GO:0005158 | 7 | 5 | 0.01081 | 0.057598 | insulin receptor binding | 0.714286 |  |  |  |  |  |  |
| GO:0005242 | 7 | 5 | 0.01081 | 0.057598 | inward rectifier potassium channel activity | 0.714286 |  |  |  |  |  |  |
| GO:0007420 | 7 | 5 | 0.01081 | 0.057598 | brain development | 0.714286 |  |  |  |  |  |  |
| GO:0008093 | 7 | 5 | 0.01081 | 0.057598 | cytoskeletal adaptor activity | 0.714286 |  |  |  |  |  |  |
| GO:0030317 | 7 | 5 | 0.01081 | 0.057598 | sperm motility | 0.714286 |  |  |  |  |  |  |
| GO:0042373 | 7 | 5 | 0.01081 | 0.057598 | vitamin K metabolic process | 0.714286 |  |  |  |  |  |  |
| GO:0046847 | 7 | 5 | 0.01081 | 0.057598 | filopodium assembly | 0.714286 |  |  |  |  |  |  |
| GO:0047021 | 7 | 5 | 0.01081 | 0.057598 | 15-hydroxyprostaglandin dehydrogenase (NADP+) activity | 0.714286 |  |  |  |  |  |  |
| GO:0048488 | 7 | 5 | 0.01081 | 0.057598 | synaptic vesicle endocytosis | 0.714286 |  |  |  |  |  |  |
| GO:0050221 | 7 | 5 | 0.01081 | 0.057598 | prostaglandin-E2 9-reductase activity | 0.714286 |  |  |  |  |  |  |
| GO:0004683 | 24 | 17 | 0.000122 | 0.001136 | calmodulin-dependent protein kinase activity | 0.708333 |  |  |  |  |  |  |
| GO:0004714 | 24 | 17 | 0.000122 | 0.001136 | transmembrane receptor protein tyrosine kinase activity | 0.708333 |  |  |  |  |  |  |
| GO:0008241 | 17 | 12 | 0.000843 | 0.006313 | peptidyl-dipeptidase activity | 0.705882 |  |  |  |  |  |  |
| GO:0019236 | 17 | 12 | 0.000843 | 0.006313 | response to pheromone | 0.705882 |  |  |  |  |  |  |
| GO:0046777 | 17 | 12 | 0.000843 | 0.006313 | protein autophosphorylation | 0.705882 |  |  |  |  |  |  |
| GO:0006032 | 40 | 28 | 3.16E-06 | 3.90E-05 | chitin catabolic process | 0.7 |  |  |  |  |  |  |
| GO:0004089 | 20 | 14 | 0.000466 | 0.003843 | carbonate dehydratase activity | 0.7 |  |  |  |  |  |  |
| GO:0001707 | 10 | 7 | 0.006079 | 0.035561 | mesoderm formation | 0.7 |  |  |  |  |  |  |
| GO:0046907 | 10 | 7 | 0.006079 | 0.035561 | intracellular transport | 0.7 |  |  |  |  |  |  |
| GO:0051726 | 36 | 25 | 1.16E-05 | 0.000128 | regulation of cell cycle | 0.694444 |  |  |  |  |  |  |
| GO:0016717 | 52 | 36 | 3.00E-07 | 3.88E-06 | oxidoreductase activity. acting on paired donors. with oxidation of a pair of donors resulting in the reduction of molecular oxygen to two molecules of water | 0.692308 |  |  |  |  |  |  |
| GO:0005667 | 32 | 22 | 4.26E-05 | 0.000425 | transcription factor complex | 0.6875 |  |  |  |  |  |  |
| GO:0004879 | 16 | 11 | 0.001808 | 0.012664 | ligand-activated sequence-specific DNA binding RNA polymerase II transcription factor activity | 0.6875 |  |  |  |  |  |  |
| GO:0030522 | 16 | 11 | 0.001808 | 0.012664 | intracellular receptor mediated signaling pathway | 0.6875 |  |  |  |  |  |  |
| GO:0007507 | 22 | 15 | 0.000532 | 0.004317 | heart development | 0.681818 |  |  |  |  |  |  |
| GO:0004623 | 25 | 17 | 0.000289 | 0.002507 | phospholipase A2 activity | 0.68 |  |  |  |  |  |  |
| GO:0007229 | 28 | 19 | 0.000157 | 0.001447 | integrin-mediated signaling pathway | 0.678571 |  |  |  |  |  |  |
| GO:0005102 | 27 | 18 | 0.000315 | 0.002665 | receptor binding | 0.666667 |  |  |  |  |  |  |
| GO:0019898 | 15 | 10 | 0.00381 | 0.024544 | extrinsic to membrane | 0.666667 |  |  |  |  |  |  |
| GO:0045727 | 15 | 10 | 0.00381 | 0.024544 | positive regulation of translation | 0.666667 |  |  |  |  |  |  |
| GO:0005802 | 12 | 8 | 0.007194 | 0.04057 | trans-Golgi network | 0.666667 |  |  |  |  |  |  |
| GO:0006338 | 12 | 8 | 0.007194 | 0.04057 | chromatin remodeling | 0.666667 |  |  |  |  |  |  |
| GO:0008305 | 12 | 8 | 0.007194 | 0.04057 | integrin complex | 0.666667 |  |  |  |  |  |  |
| GO:0015672 | 12 | 8 | 0.007194 | 0.04057 | monovalent inorganic cation transport | 0.666667 |  |  |  |  |  |  |
| GO:0060070 | 12 | 8 | 0.007194 | 0.04057 | canonical Wnt receptor signaling pathway | 0.666667 |  |  |  |  |  |  |
| GO:0000159 | 9 | 6 | 0.013659 | 0.071351 | protein phosphatase type 2A complex | 0.666667 |  |  |  |  |  |  |
| GO:0005086 | 9 | 6 | 0.013659 | 0.071351 | ARF guanyl-nucleotide exchange factor activity | 0.666667 |  |  |  |  |  |  |
| GO:0006004 | 9 | 6 | 0.013659 | 0.071351 | fucose metabolic process | 0.666667 |  |  |  |  |  |  |
| GO:0006493 | 9 | 6 | 0.013659 | 0.071351 | protein O-linked glycosylation | 0.666667 |  |  |  |  |  |  |
| GO:0019028 | 9 | 6 | 0.013659 | 0.071351 | viral capsid | 0.666667 |  |  |  |  |  |  |
| GO:0032012 | 9 | 6 | 0.013659 | 0.071351 | regulation of ARF protein signal transduction | 0.666667 |  |  |  |  |  |  |
| GO:0003917 | 6 | 4 | 0.025945 | 0.113029 | DNA topoisomerase type I activity | 0.666667 |  |  |  |  |  |  |
| GO:0004075 | 6 | 4 | 0.025945 | 0.113029 | biotin carboxylase activity | 0.666667 |  |  |  |  |  |  |
| GO:0004122 | 6 | 4 | 0.025945 | 0.113029 | cystathionine beta-synthase activity | 0.666667 |  |  |  |  |  |  |
| GO:0004303 | 6 | 4 | 0.025945 | 0.113029 | estradiol 17-beta-dehydrogenase activity | 0.666667 |  |  |  |  |  |  |
| GO:0008023 | 6 | 4 | 0.025945 | 0.113029 | transcription elongation factor complex | 0.666667 |  |  |  |  |  |  |
| GO:0008177 | 6 | 4 | 0.025945 | 0.113029 | succinate dehydrogenase (ubiquinone) activity | 0.666667 |  |  |  |  |  |  |
| GO:0008527 | 6 | 4 | 0.025945 | 0.113029 | taste receptor activity | 0.666667 |  |  |  |  |  |  |
| GO:0008607 | 6 | 4 | 0.025945 | 0.113029 | phosphorylase kinase regulator activity | 0.666667 |  |  |  |  |  |  |
| GO:0009952 | 6 | 4 | 0.025945 | 0.113029 | anterior/posterior pattern specification | 0.666667 |  |  |  |  |  |  |
| GO:0015979 | 6 | 4 | 0.025945 | 0.113029 | photosynthesis | 0.666667 |  |  |  |  |  |  |
| GO:0016715 | 6 | 4 | 0.025945 | 0.113029 | oxidoreductase activity. acting on paired donors. with incorporation or reduction of molecular oxygen. reduced ascorbate as one donor. and incorporation of one atom of oxygen | 0.666667 |  |  |  |  |  |  |
| GO:0018095 | 6 | 4 | 0.025945 | 0.113029 | protein polyglutamylation | 0.666667 |  |  |  |  |  |  |
| GO:0019343 | 6 | 4 | 0.025945 | 0.113029 | cysteine biosynthetic process via cystathionine | 0.666667 |  |  |  |  |  |  |
| GO:0030027 | 6 | 4 | 0.025945 | 0.113029 | lamellipodium | 0.666667 |  |  |  |  |  |  |
| GO:0050912 | 6 | 4 | 0.025945 | 0.113029 | detection of chemical stimulus involved in sensory perception of taste | 0.666667 |  |  |  |  |  |  |
| GO:0051020 | 6 | 4 | 0.025945 | 0.113029 | GTPase binding | 0.666667 |  |  |  |  |  |  |
| GO:0060027 | 6 | 4 | 0.025945 | 0.113029 | convergent extension involved in gastrulation | 0.666667 |  |  |  |  |  |  |
| GO:0071920 | 6 | 4 | 0.025945 | 0.113029 | cleavage body | 0.666667 |  |  |  |  |  |  |
| GO:0005938 | 26 | 17 | 0.000621 | 0.004986 | cell cortex | 0.653846 |  |  |  |  |  |  |
| GO:0000079 | 34 | 22 | 0.000188 | 0.001684 | regulation of cyclin-dependent protein kinase activity | 0.647059 |  |  |  |  |  |  |
| GO:0004114 | 14 | 9 | 0.00787 | 0.044033 | 3'.5'-cyclic-nucleotide phosphodiesterase activity | 0.642857 |  |  |  |  |  |  |
| GO:0008601 | 14 | 9 | 0.00787 | 0.044033 | protein phosphatase type 2A regulator activity | 0.642857 |  |  |  |  |  |  |
| GO:0044262 | 25 | 16 | 0.001209 | 0.008859 | cellular carbohydrate metabolic process | 0.64 |  |  |  |  |  |  |
| GO:0006813 | 61 | 39 | 2.84E-06 | 3.53E-05 | potassium ion transport | 0.639344 |  |  |  |  |  |  |
| GO:0005847 | 11 | 7 | 0.015215 | 0.077944 | mRNA cleavage and polyadenylation specificity factor complex | 0.636364 |  |  |  |  |  |  |
| GO:0009966 | 11 | 7 | 0.015215 | 0.077944 | regulation of signal transduction | 0.636364 |  |  |  |  |  |  |
| GO:0031105 | 11 | 7 | 0.015215 | 0.077944 | septin complex | 0.636364 |  |  |  |  |  |  |
| GO:0005391 | 19 | 12 | 0.0043 | 0.027288 | sodium:potassium-exchanging ATPase activity | 0.631579 |  |  |  |  |  |  |
| GO:0033961 | 19 | 12 | 0.0043 | 0.027288 | cis-stilbene-oxide hydrolase activity | 0.631579 |  |  |  |  |  |  |
| GO:0005244 | 35 | 22 | 0.00036 | 0.003016 | voltage-gated ion channel activity | 0.628571 |  |  |  |  |  |  |
| GO:0034765 | 35 | 22 | 0.00036 | 0.003016 | regulation of ion transmembrane transport | 0.628571 |  |  |  |  |  |  |
| GO:0019001 | 32 | 20 | 0.000668 | 0.005337 | guanyl nucleotide binding | 0.625 |  |  |  |  |  |  |
| GO:0004697 | 24 | 15 | 0.002315 | 0.015508 | protein kinase C activity | 0.625 |  |  |  |  |  |  |
| GO:0070979 | 16 | 10 | 0.008213 | 0.045482 | protein K11-linked ubiquitination | 0.625 |  |  |  |  |  |  |
| GO:0004602 | 8 | 5 | 0.029965 | 0.126459 | glutathione peroxidase activity | 0.625 |  |  |  |  |  |  |
| GO:0006301 | 8 | 5 | 0.029965 | 0.126459 | postreplication repair | 0.625 |  |  |  |  |  |  |
| GO:0006513 | 8 | 5 | 0.029965 | 0.126459 | protein monoubiquitination | 0.625 |  |  |  |  |  |  |
| GO:0009411 | 8 | 5 | 0.029965 | 0.126459 | response to UV | 0.625 |  |  |  |  |  |  |
| GO:0050750 | 8 | 5 | 0.029965 | 0.126459 | low-density lipoprotein particle receptor binding | 0.625 |  |  |  |  |  |  |
| GO:0008299 | 42 | 26 | 0.000194 | 0.001737 | isoprenoid biosynthetic process | 0.619048 |  |  |  |  |  |  |
| GO:0004568 | 26 | 16 | 0.00232 | 0.015508 | chitinase activity | 0.615385 |  |  |  |  |  |  |
| GO:0009331 | 13 | 8 | 0.015893 | 0.079446 | glycerol-3-phosphate dehydrogenase complex | 0.615385 |  |  |  |  |  |  |
| GO:0030247 | 13 | 8 | 0.015893 | 0.079446 | polysaccharide binding | 0.615385 |  |  |  |  |  |  |
| GO:0003707 | 59 | 36 | 3.00E-05 | 0.000314 | steroid hormone receptor activity | 0.610169 |  |  |  |  |  |  |
| GO:0043401 | 59 | 36 | 3.00E-05 | 0.000314 | steroid hormone mediated signaling pathway | 0.610169 |  |  |  |  |  |  |
| GO:0030246 | 33 | 20 | 0.001213 | 0.008859 | carbohydrate binding | 0.606061 |  |  |  |  |  |  |
| GO:0016773 | 78 | 47 | 4.45E-06 | 5.40E-05 | phosphotransferase activity. alcohol group as acceptor | 0.602564 |  |  |  |  |  |  |
| GO:0005496 | 25 | 15 | 0.004282 | 0.027288 | steroid binding | 0.6 |  |  |  |  |  |  |
| GO:0005319 | 20 | 12 | 0.008236 | 0.045482 | lipid transporter activity | 0.6 |  |  |  |  |  |  |
| GO:0005925 | 20 | 12 | 0.008236 | 0.045482 | focal adhesion | 0.6 |  |  |  |  |  |  |
| GO:0030016 | 20 | 12 | 0.008236 | 0.045482 | myofibril | 0.6 |  |  |  |  |  |  |
| GO:0004046 | 15 | 9 | 0.015992 | 0.079446 | aminoacylase activity | 0.6 |  |  |  |  |  |  |
| GO:0005618 | 15 | 9 | 0.015992 | 0.079446 | cell wall | 0.6 |  |  |  |  |  |  |
| GO:0030672 | 15 | 9 | 0.015992 | 0.079446 | synaptic vesicle membrane | 0.6 |  |  |  |  |  |  |
| GO:0042423 | 15 | 9 | 0.015992 | 0.079446 | catecholamine biosynthetic process | 0.6 |  |  |  |  |  |  |
| GO:0000086 | 10 | 6 | 0.031347 | 0.126459 | G2/M transition of mitotic cell cycle | 0.6 |  |  |  |  |  |  |
| GO:0003689 | 10 | 6 | 0.031347 | 0.126459 | DNA clamp loader activity | 0.6 |  |  |  |  |  |  |
| GO:0003939 | 10 | 6 | 0.031347 | 0.126459 | L-iditol 2-dehydrogenase activity | 0.6 |  |  |  |  |  |  |
| GO:0004370 | 10 | 6 | 0.031347 | 0.126459 | glycerol kinase activity | 0.6 |  |  |  |  |  |  |
| GO:0004462 | 10 | 6 | 0.031347 | 0.126459 | lactoylglutathione lyase activity | 0.6 |  |  |  |  |  |  |
| GO:0005663 | 10 | 6 | 0.031347 | 0.126459 | DNA replication factor C complex | 0.6 |  |  |  |  |  |  |
| GO:0006265 | 10 | 6 | 0.031347 | 0.126459 | DNA topological change | 0.6 |  |  |  |  |  |  |
| GO:0008889 | 10 | 6 | 0.031347 | 0.126459 | glycerophosphodiester phosphodiesterase activity | 0.6 |  |  |  |  |  |  |
| GO:0030976 | 10 | 6 | 0.031347 | 0.126459 | thiamine pyrophosphate binding | 0.6 |  |  |  |  |  |  |
| GO:0043065 | 10 | 6 | 0.031347 | 0.126459 | positive regulation of apoptotic process | 0.6 |  |  |  |  |  |  |
| GO:0051087 | 10 | 6 | 0.031347 | 0.126459 | chaperone binding | 0.6 |  |  |  |  |  |  |
| GO:0019901 | 52 | 31 | 0.000172 | 0.001569 | protein kinase binding | 0.596154 |  |  |  |  |  |  |
| GO:0004871 | 297 | 177 | 4.34E-17 | 6.68E-16 | signal transducer activity | 0.59596 |  |  |  |  |  |  |
| GO:0005516 | 42 | 25 | 0.000606 | 0.004879 | calmodulin binding | 0.595238 |  |  |  |  |  |  |
| GO:0035556 | 143 | 85 | 4.32E-09 | 5.96E-08 | intracellular signal transduction | 0.594406 |  |  |  |  |  |  |
| GO:0007165 | 546 | 324 | 1.82E-29 | 2.84E-28 | signal transduction | 0.593407 |  |  |  |  |  |  |
| GO:0006072 | 22 | 13 | 0.008038 | 0.044854 | glycerol-3-phosphate metabolic process | 0.590909 |  |  |  |  |  |  |
| GO:0004185 | 17 | 10 | 0.015712 | 0.079446 | serine-type carboxypeptidase activity | 0.588235 |  |  |  |  |  |  |
| GO:0004767 | 17 | 10 | 0.015712 | 0.079446 | sphingomyelin phosphodiesterase activity | 0.588235 |  |  |  |  |  |  |
| GO:0080030 | 75 | 44 | 2.22E-05 | 0.000236 | methyl indole-3-acetate esterase activity | 0.586667 |  |  |  |  |  |  |
| GO:0080031 | 75 | 44 | 2.22E-05 | 0.000236 | methyl salicylate esterase activity | 0.586667 |  |  |  |  |  |  |
| GO:0080032 | 75 | 44 | 2.22E-05 | 0.000236 | methyl jasmonate esterase activity | 0.586667 |  |  |  |  |  |  |
| GO:0043565 | 360 | 210 | 1.98E-18 | 3.06E-17 | sequence-specific DNA binding | 0.583333 |  |  |  |  |  |  |
| GO:0004221 | 60 | 35 | 0.000144 | 0.001333 | ubiquitin thiolesterase activity | 0.583333 |  |  |  |  |  |  |
| GO:0001701 | 12 | 7 | 0.031256 | 0.126459 | in utero embryonic development | 0.583333 |  |  |  |  |  |  |
| GO:0004707 | 12 | 7 | 0.031256 | 0.126459 | MAP kinase activity | 0.583333 |  |  |  |  |  |  |
| GO:0007409 | 12 | 7 | 0.031256 | 0.126459 | axonogenesis | 0.583333 |  |  |  |  |  |  |
| GO:0008104 | 12 | 7 | 0.031256 | 0.126459 | protein localization | 0.583333 |  |  |  |  |  |  |
| GO:0010430 | 12 | 7 | 0.031256 | 0.126459 | fatty acid omega-oxidation | 0.583333 |  |  |  |  |  |  |
| GO:0009636 | 31 | 18 | 0.003842 | 0.024679 | response to toxin | 0.580645 |  |  |  |  |  |  |
| GO:0005089 | 19 | 11 | 0.015191 | 0.077944 | Rho guanyl-nucleotide exchange factor activity | 0.578947 |  |  |  |  |  |  |
| GO:0035023 | 19 | 11 | 0.015191 | 0.077944 | regulation of Rho protein signal transduction | 0.578947 |  |  |  |  |  |  |
| GO:0042995 | 97 | 56 | 4.73E-06 | 5.72E-05 | cell projection | 0.57732 |  |  |  |  |  |  |
| GO:0042626 | 52 | 30 | 0.000482 | 0.00393 | ATPase activity. coupled to transmembrane movement of substances | 0.576923 |  |  |  |  |  |  |
| GO:0004693 | 26 | 15 | 0.007422 | 0.041743 | cyclin-dependent protein kinase activity | 0.576923 |  |  |  |  |  |  |
| GO:0015074 | 92 | 53 | 8.75E-06 | 9.69E-05 | DNA integration | 0.576087 |  |  |  |  |  |  |
| GO:0004344 | 33 | 19 | 0.003658 | 0.02371 | glucose dehydrogenase activity | 0.575758 |  |  |  |  |  |  |
| GO:0000226 | 40 | 23 | 0.001817 | 0.012664 | microtubule cytoskeleton organization | 0.575 |  |  |  |  |  |  |
| GO:0008134 | 40 | 23 | 0.001817 | 0.012664 | transcription factor binding | 0.575 |  |  |  |  |  |  |
| GO:0032313 | 40 | 23 | 0.001817 | 0.012664 | regulation of Rab GTPase activity | 0.575 |  |  |  |  |  |  |
| GO:0016023 | 28 | 16 | 0.007054 | 0.040313 | cytoplasmic membrane-bounded vesicle | 0.571429 |  |  |  |  |  |  |
| GO:0004437 | 21 | 12 | 0.014521 | 0.075297 | inositol or phosphatidylinositol phosphatase activity | 0.571429 |  |  |  |  |  |  |
| GO:0019439 | 21 | 12 | 0.014521 | 0.075297 | aromatic compound catabolic process | 0.571429 |  |  |  |  |  |  |
| GO:0004854 | 14 | 8 | 0.030335 | 0.126459 | xanthine dehydrogenase activity | 0.571429 |  |  |  |  |  |  |
| GO:0004855 | 14 | 8 | 0.030335 | 0.126459 | xanthine oxidase activity | 0.571429 |  |  |  |  |  |  |
| GO:0008060 | 14 | 8 | 0.030335 | 0.126459 | ARF GTPase activator activity | 0.571429 |  |  |  |  |  |  |
| GO:0008559 | 14 | 8 | 0.030335 | 0.126459 | xenobiotic-transporting ATPase activity | 0.571429 |  |  |  |  |  |  |
| GO:0009115 | 14 | 8 | 0.030335 | 0.126459 | xanthine catabolic process | 0.571429 |  |  |  |  |  |  |
| GO:0032312 | 14 | 8 | 0.030335 | 0.126459 | regulation of ARF GTPase activity | 0.571429 |  |  |  |  |  |  |
| GO:0043546 | 14 | 8 | 0.030335 | 0.126459 | molybdopterin cofactor binding | 0.571429 |  |  |  |  |  |  |
| GO:0060047 | 14 | 8 | 0.030335 | 0.126459 | heart contraction | 0.571429 |  |  |  |  |  |  |
| GO:0008081 | 44 | 25 | 0.001621 | 0.011685 | phosphoric diester hydrolase activity | 0.568182 |  |  |  |  |  |  |
| GO:0016798 | 162 | 92 | 2.71E-08 | 3.67E-07 | hydrolase activity. acting on glycosyl bonds | 0.567901 |  |  |  |  |  |  |
| GO:0008643 | 104 | 59 | 6.11E-06 | 7.06E-05 | carbohydrate transport | 0.567308 |  |  |  |  |  |  |
| GO:0004713 | 78 | 44 | 8.57E-05 | 0.000821 | protein tyrosine kinase activity | 0.564103 |  |  |  |  |  |  |
| GO:0005097 | 39 | 22 | 0.00308 | 0.020156 | Rab GTPase activator activity | 0.564103 |  |  |  |  |  |  |
| GO:0009986 | 39 | 22 | 0.00308 | 0.020156 | cell surface | 0.564103 |  |  |  |  |  |  |
| GO:0032851 | 39 | 22 | 0.00308 | 0.020156 | positive regulation of Rab GTPase activity | 0.564103 |  |  |  |  |  |  |
| GO:0004553 | 144 | 81 | 2.84E-07 | 3.70E-06 | hydrolase activity. hydrolyzing O-glycosyl compounds | 0.5625 |  |  |  |  |  |  |
| GO:0007169 | 16 | 9 | 0.028956 | 0.124365 | transmembrane receptor protein tyrosine kinase signaling pathway | 0.5625 |  |  |  |  |  |  |
| GO:0009408 | 16 | 9 | 0.028956 | 0.124365 | response to heat | 0.5625 |  |  |  |  |  |  |
| GO:0017124 | 16 | 9 | 0.028956 | 0.124365 | SH3 domain binding | 0.5625 |  |  |  |  |  |  |
| GO:0007186 | 249 | 139 | 7.13E-11 | 1.04E-09 | G-protein coupled receptor signaling pathway | 0.558233 |  |  |  |  |  |  |
| GO:0003333 | 18 | 10 | 0.02734 | 0.118382 | amino acid transmembrane transport | 0.555556 |  |  |  |  |  |  |
| GO:0009405 | 18 | 10 | 0.02734 | 0.118382 | pathogenesis | 0.555556 |  |  |  |  |  |  |
| GO:0015171 | 18 | 10 | 0.02734 | 0.118382 | amino acid transmembrane transporter activity | 0.555556 |  |  |  |  |  |  |
| GO:0016459 | 60 | 33 | 0.000948 | 0.007045 | myosin complex | 0.55 |  |  |  |  |  |  |
| GO:0001522 | 20 | 11 | 0.025622 | 0.113029 | pseudouridine synthesis | 0.55 |  |  |  |  |  |  |
| GO:0004888 | 66 | 36 | 0.00076 | 0.005981 | transmembrane signaling receptor activity | 0.545455 |  |  |  |  |  |  |
| GO:0004301 | 22 | 12 | 0.023885 | 0.108968 | epoxide hydrolase activity | 0.545455 |  |  |  |  |  |  |
| GO:0005044 | 22 | 12 | 0.023885 | 0.108968 | scavenger receptor activity | 0.545455 |  |  |  |  |  |  |
| GO:0007155 | 158 | 86 | 9.12E-07 | 1.15E-05 | cell adhesion | 0.544304 |  |  |  |  |  |  |
| GO:0003700 | 480 | 261 | 6.42E-17 | 9.82E-16 | sequence-specific DNA binding transcription factor activity | 0.54375 |  |  |  |  |  |  |
| GO:0006066 | 48 | 26 | 0.003551 | 0.023088 | alcohol metabolic process | 0.541667 |  |  |  |  |  |  |
| GO:0008812 | 48 | 26 | 0.003551 | 0.023088 | choline dehydrogenase activity | 0.541667 |  |  |  |  |  |  |
| GO:0000036 | 24 | 13 | 0.022179 | 0.102061 | ACP phosphopantetheine attachment site binding involved in fatty acid biosynthetic process | 0.541667 |  |  |  |  |  |  |
| GO:0004725 | 85 | 46 | 0.000243 | 0.002129 | protein tyrosine phosphatase activity | 0.541176 |  |  |  |  |  |  |
| GO:0035335 | 85 | 46 | 0.000243 | 0.002129 | peptidyl-tyrosine dephosphorylation | 0.541176 |  |  |  |  |  |  |
| GO:0004930 | 182 | 98 | 3.63E-07 | 4.62E-06 | G-protein coupled receptor activity | 0.538462 |  |  |  |  |  |  |
| GO:0003993 | 26 | 14 | 0.020535 | 0.094908 | acid phosphatase activity | 0.538462 |  |  |  |  |  |  |
| GO:0006278 | 245 | 131 | 9.77E-09 | 1.34E-07 | RNA-dependent DNA replication | 0.534694 |  |  |  |  |  |  |
| GO:0005929 | 45 | 24 | 0.00614 | 0.035561 | cilium | 0.533333 |  |  |  |  |  |  |
| GO:0019285 | 32 | 17 | 0.016116 | 0.079446 | glycine betaine biosynthetic process from choline | 0.53125 |  |  |  |  |  |  |
| GO:0030054 | 162 | 86 | 3.64E-06 | 4.47E-05 | cell junction | 0.530864 |  |  |  |  |  |  |
| GO:0016597 | 17 | 9 | 0.047876 | 0.188142 | amino acid binding | 0.529412 |  |  |  |  |  |  |
| GO:0043169 | 155 | 82 | 6.99E-06 | 7.86E-05 | cation binding | 0.529032 |  |  |  |  |  |  |
| GO:0003779 | 159 | 84 | 5.94E-06 | 6.91E-05 | actin binding | 0.528302 |  |  |  |  |  |  |
| GO:0016772 | 459 | 242 | 8.67E-14 | 1.28E-12 | transferase activity. transferring phosphorus-containing groups | 0.527233 |  |  |  |  |  |  |
| GO:0008092 | 19 | 10 | 0.044046 | 0.173732 | cytoskeletal protein binding | 0.526316 |  |  |  |  |  |  |
| GO:0008287 | 19 | 10 | 0.044046 | 0.173732 | protein serine/threonine phosphatase complex | 0.526316 |  |  |  |  |  |  |
| GO:0006814 | 99 | 52 | 0.000303 | 0.002575 | sodium ion transport | 0.525253 |  |  |  |  |  |  |
| GO:0005581 | 21 | 11 | 0.040424 | 0.160638 | collagen | 0.52381 |  |  |  |  |  |  |
| GO:0016811 | 21 | 11 | 0.040424 | 0.160638 | hydrolase activity. acting on carbon-nitrogen (but not peptide) bonds. in linear amides | 0.52381 |  |  |  |  |  |  |
| GO:0004867 | 138 | 72 | 4.18E-05 | 0.000419 | serine-type endopeptidase inhibitor activity | 0.521739 |  |  |  |  |  |  |
| GO:0004674 | 319 | 166 | 1.98E-09 | 2.77E-08 | protein serine/threonine kinase activity | 0.520376 |  |  |  |  |  |  |
| GO:0003774 | 79 | 41 | 0.001471 | 0.010675 | motor activity | 0.518987 |  |  |  |  |  |  |
| GO:0006470 | 109 | 56 | 0.000408 | 0.003391 | protein dephosphorylation | 0.513761 |  |  |  |  |  |  |
| GO:0016311 | 111 | 57 | 0.000375 | 0.003125 | dephosphorylation | 0.513514 |  |  |  |  |  |  |
| GO:0005085 | 39 | 20 | 0.018027 | 0.083497 | guanyl-nucleotide exchange factor activity | 0.512821 |  |  |  |  |  |  |
| GO:0016301 | 670 | 343 | 2.18E-16 | 3.30E-15 | kinase activity | 0.51194 |  |  |  |  |  |  |
| GO:0004222 | 86 | 44 | 0.00156 | 0.011283 | metalloendopeptidase activity | 0.511628 |  |  |  |  |  |  |
| GO:0016310 | 672 | 343 | 3.82E-16 | 5.72E-15 | phosphorylation | 0.510417 |  |  |  |  |  |  |
| GO:0005874 | 198 | 101 | 6.61E-06 | 7.47E-05 | microtubule | 0.510101 |  |  |  |  |  |  |
| GO:0004672 | 415 | 211 | 2.46E-10 | 3.54E-09 | protein kinase activity | 0.508434 |  |  |  |  |  |  |
| GO:0007586 | 69 | 35 | 0.004683 | 0.029028 | digestion | 0.507246 |  |  |  |  |  |  |
| GO:0006468 | 420 | 213 | 2.73E-10 | 3.90E-09 | protein phosphorylation | 0.507143 |  |  |  |  |  |  |
| GO:0016757 | 296 | 150 | 1.03E-07 | 1.35E-06 | transferase activity. transferring glycosyl groups | 0.506757 |  |  |  |  |  |  |
| GO:0016791 | 103 | 52 | 0.001047 | 0.007752 | phosphatase activity | 0.504854 |  |  |  |  |  |  |
| GO:0005506 | 442 | 223 | 1.99E-10 | 2.87E-09 | iron ion binding | 0.504525 |  |  |  |  |  |  |
| GO:0005886 | 775 | 388 | 2.40E-16 | 3.62E-15 | plasma membrane | 0.500645 |  |  |  |  |  |  |
| GO:0016055 | 60 | 30 | 0.009969 | 0.054909 | Wnt receptor signaling pathway | 0.5 |  |  |  |  |  |  |
| GO:0005578 | 42 | 21 | 0.022966 | 0.105453 | proteinaceous extracellular matrix | 0.5 |  |  |  |  |  |  |
| GO:0004722 | 36 | 18 | 0.030521 | 0.126459 | protein serine/threonine phosphatase activity | 0.5 |  |  |  |  |  |  |
| GO:0019904 | 36 | 18 | 0.030521 | 0.126459 | protein domain specific binding | 0.5 |  |  |  |  |  |  |
| GO:0007283 | 34 | 17 | 0.033586 | 0.134514 | spermatogenesis | 0.5 |  |  |  |  |  |  |
| GO:0007219 | 32 | 16 | 0.036976 | 0.147764 | Notch signaling pathway | 0.5 |  |  |  |  |  |  |
| GO:0004889 | 30 | 15 | 0.040728 | 0.161544 | acetylcholine-activated cation-selective channel activity | 0.5 |  |  |  |  |  |  |
| GO:0009451 | 26 | 13 | 0.049491 | 0.193773 | RNA modification | 0.5 |  |  |  |  |  |  |
| GO:0005216 | 179 | 89 | 7.27E-05 | 0.000706 | ion channel activity | 0.497207 |  |  |  |  |  |  |
| GO:0007218 | 159 | 79 | 0.000173 | 0.001569 | neuropeptide signaling pathway | 0.496855 |  |  |  |  |  |  |
| GO:0006633 | 137 | 68 | 0.000449 | 0.003716 | fatty acid biosynthetic process | 0.49635 |  |  |  |  |  |  |
| GO:0006200 | 166 | 82 | 0.000173 | 0.001569 | ATP catabolic process | 0.493976 |  |  |  |  |  |  |
| GO:0006812 | 51 | 25 | 0.021332 | 0.098378 | cation transport | 0.490196 |  |  |  |  |  |  |
| GO:0016887 | 164 | 80 | 0.00034 | 0.002865 | ATPase activity | 0.487805 |  |  |  |  |  |  |
| GO:0003964 | 279 | 136 | 5.67E-06 | 6.63E-05 | RNA-directed DNA polymerase activity | 0.487455 |  |  |  |  |  |  |
| GO:0004721 | 119 | 58 | 0.001815 | 0.012664 | phosphoprotein phosphatase activity | 0.487395 |  |  |  |  |  |  |
| GO:0007154 | 39 | 19 | 0.037983 | 0.151503 | cell communication | 0.487179 |  |  |  |  |  |  |
| GO:0022857 | 139 | 67 | 0.001342 | 0.009772 | transmembrane transporter activity | 0.482014 |  |  |  |  |  |  |
| GO:0005792 | 247 | 119 | 3.86E-05 | 0.000389 | microsome | 0.481781 |  |  |  |  |  |  |
| GO:0045202 | 113 | 54 | 0.004213 | 0.02698 | synapse | 0.477876 |  |  |  |  |  |  |
| GO:0030286 | 44 | 21 | 0.04163 | 0.164815 | dynein complex | 0.477273 |  |  |  |  |  |  |
| GO:0004104 | 42 | 20 | 0.045936 | 0.180852 | cholinesterase activity | 0.47619 |  |  |  |  |  |  |
| GO:0006629 | 328 | 156 | 7.83E-06 | 8.72E-05 | lipid metabolic process | 0.47561 |  |  |  |  |  |  |
| GO:0042470 | 91 | 43 | 0.011256 | 0.059827 | melanosome | 0.472527 |  |  |  |  |  |  |
| GO:0004190 | 68 | 32 | 0.02431 | 0.110671 | aspartic-type endopeptidase activity | 0.470588 |  |  |  |  |  |  |
| GO:0005509 | 440 | 205 | 2.27E-06 | 2.84E-05 | calcium ion binding | 0.465909 |  |  |  |  |  |  |
| GO:0008610 | 202 | 94 | 0.000936 | 0.006985 | lipid biosynthetic process | 0.465347 |  |  |  |  |  |  |
| GO:0050660 | 175 | 81 | 0.002283 | 0.015356 | flavin adenine dinucleotide binding | 0.462857 |  |  |  |  |  |  |
| GO:0020037 | 394 | 182 | 1.48E-05 | 0.00016 | heme binding | 0.461929 |  |  |  |  |  |  |
| GO:0004091 | 104 | 48 | 0.013977 | 0.072831 | carboxylesterase activity | 0.461538 |  |  |  |  |  |  |
| GO:0006979 | 76 | 35 | 0.029171 | 0.125037 | response to oxidative stress | 0.460526 |  |  |  |  |  |  |
| GO:0004180 | 87 | 40 | 0.023024 | 0.105493 | carboxypeptidase activity | 0.45977 |  |  |  |  |  |  |
| GO:0016705 | 296 | 136 | 0.000201 | 0.001788 | oxidoreductase activity. acting on paired donors. with incorporation or reduction of molecular oxygen | 0.459459 |  |  |  |  |  |  |
| GO:0005515 | 122 | 56 | 0.010494 | 0.057598 | protein binding | 0.459016 |  |  |  |  |  |  |
| GO:0016614 | 83 | 38 | 0.027553 | 0.119059 | oxidoreductase activity. acting on CH-OH group of donors | 0.457831 |  |  |  |  |  |  |
| GO:0016758 | 127 | 58 | 0.010773 | 0.057598 | transferase activity. transferring hexosyl groups | 0.456693 |  |  |  |  |  |  |
| GO:0004497 | 368 | 168 | 6.54E-05 | 0.000638 | monooxygenase activity | 0.456522 |  |  |  |  |  |  |
| GO:0005975 | 368 | 167 | 0.0001 | 0.000956 | carbohydrate metabolic process | 0.453804 |  |  |  |  |  |  |
| GO:0005549 | 170 | 77 | 0.00563 | 0.034797 | odorant binding | 0.452941 |  |  |  |  |  |  |
| GO:0008236 | 228 | 103 | 0.001995 | 0.013772 | serine-type peptidase activity | 0.451754 |  |  |  |  |  |  |
| GO:0007275 | 473 | 212 | 3.69E-05 | 0.000379 | multicellular organismal development | 0.448203 |  |  |  |  |  |  |
| GO:0055085 | 621 | 274 | 1.40E-05 | 0.000154 | transmembrane transport | 0.441224 |  |  |  |  |  |  |
| GO:0004519 | 166 | 73 | 0.01538 | 0.078598 | endonuclease activity | 0.439759 |  |  |  |  |  |  |
| GO:0006511 | 116 | 51 | 0.033508 | 0.134514 | ubiquitin-dependent protein catabolic process | 0.439655 |  |  |  |  |  |  |
| GO:0005789 | 544 | 239 | 6.50E-05 | 0.000637 | endoplasmic reticulum membrane | 0.439338 |  |  |  |  |  |  |
| GO:0005576 | 1144 | 498 | 4.92E-08 | 6.50E-07 | extracellular region | 0.435315 |  |  |  |  |  |  |
| GO:0017111 | 273 | 118 | 0.006547 | 0.037516 | nucleoside-triphosphatase activity | 0.432234 |  |  |  |  |  |  |
| GO:0016021 | 2638 | 1123 | 7.96E-14 | 1.18E-12 | integral to membrane | 0.425701 |  |  |  |  |  |  |
| GO:0005615 | 369 | 157 | 0.00459 | 0.028535 | extracellular space | 0.425474 |  |  |  |  |  |  |
| GO:0004252 | 275 | 117 | 0.011937 | 0.063288 | serine-type endopeptidase activity | 0.425455 |  |  |  |  |  |  |
| GO:0006508 | 706 | 299 | 0.000238 | 0.002103 | proteolysis | 0.423513 |  |  |  |  |  |  |
| GO:0009055 | 437 | 185 | 0.003081 | 0.020156 | electron carrier activity | 0.423341 |  |  |  |  |  |  |
| GO:0051301 | 215 | 91 | 0.025926 | 0.113029 | cell division | 0.423256 |  |  |  |  |  |  |
| GO:0008270 | 1572 | 659 | 3.60E-07 | 4.61E-06 | zinc ion binding | 0.419211 |  |  |  |  |  |  |
| GO:0004872 | 810 | 337 | 0.000477 | 0.003918 | receptor activity | 0.416049 |  |  |  |  |  |  |
| GO:0046872 | 2459 | 1021 | 1.50E-09 | 2.11E-08 | metal ion binding | 0.415209 |  |  |  |  |  |  |
| GO:0016020 | 3481 | 1443 | 2.62E-13 | 3.84E-12 | membrane | 0.414536 |  |  |  |  |  |  |
| GO:0003824 | 1474 | 607 | 1.48E-05 | 0.00016 | catalytic activity | 0.411805 |  |  |  |  |  |  |
| GO:0005215 | 317 | 130 | 0.031774 | 0.127938 | transporter activity | 0.410095 |  |  |  |  |  |  |
| GO:0005783 | 710 | 291 | 0.003035 | 0.020102 | endoplasmic reticulum | 0.409859 |  |  |  |  |  |  |
| GO:0006351 | 1087 | 440 | 0.001099 | 0.008112 | transcription. DNA-dependent | 0.404784 |  |  |  |  |  |  |
| GO:0016740 | 1605 | 646 | 0.000178 | 0.001603 | transferase activity | 0.402492 |  |  |  |  |  |  |
| GO:0016787 | 2091 | 838 | 3.75E-05 | 0.000383 | hydrolase activity | 0.400765 |  |  |  |  |  |  |
| GO:0006355 | 1257 | 502 | 0.001892 | 0.013148 | regulation of transcription. DNA-dependent | 0.399364 |  |  |  |  |  |  |
| GO:0008152 | 1362 | 542 | 0.001811 | 0.012664 | metabolic process | 0.397944 |  |  |  |  |  |  |
| GO:0003677 | 1378 | 546 | 0.00263 | 0.017525 | DNA binding | 0.396226 |  |  |  |  |  |  |
| GO:0005524 | 1708 | 672 | 0.001965 | 0.013609 | ATP binding | 0.393443 |  |  |  |  |  |  |
| GO:0055114 | 1506 | 577 | 0.033597 | 0.134514 | oxidation-reduction process | 0.383134 |  |  |  |  |  |  |
| GO:0016491 | 1396 | 533 | 0.049325 | 0.193479 | oxidoreductase activity | 0.381805 |  |  |  |  |  |  |
|  | | | | | | | |  |  |  |  |  |

Table S4 The enriched GO term for down-regulated DEGs

| GO | All_transcripts_with_GO_annotation(17360) | DETs_with_GO_annotation(2626) | Pvalue | Qvalue | GO_term | ratio |
| --- | --- | --- | --- | --- | --- | --- |
| GO:0004613 | 8 | 8 | 0 | 0 | phosphoenolpyruvate carboxykinase (GTP) activity | 1 |
| GO:0060968 | 7 | 7 | 0 | 0 | regulation of gene silencing | 1 |
| GO:0000028 | 6 | 6 | 0 | 0 | ribosomal small subunit assembly | 1 |
| GO:0004556 | 6 | 6 | 0 | 0 | alpha-amylase activity | 1 |
| GO:0006069 | 6 | 6 | 0 | 0 | ethanol oxidation | 1 |
| GO:0008747 | 6 | 6 | 0 | 0 | N-acetylneuraminate lyase activity | 1 |
| GO:0051903 | 6 | 6 | 0 | 0 | S-(hydroxymethyl)glutathione dehydrogenase activity | 1 |
| GO:0004359 | 5 | 5 | 0 | 0 | glutaminase activity | 1 |
| GO:0007338 | 5 | 5 | 0 | 0 | single fertilization | 1 |
| GO:0031088 | 5 | 5 | 0 | 0 | platelet dense granule membrane | 1 |
| GO:0032091 | 5 | 5 | 0 | 0 | negative regulation of protein binding | 1 |
| GO:0043526 | 5 | 5 | 0 | 0 | neuroprotection | 1 |
| GO:0045298 | 5 | 5 | 0 | 0 | tubulin complex | 1 |
| GO:0060548 | 5 | 5 | 0 | 0 | negative regulation of cell death | 1 |
| GO:0070301 | 5 | 5 | 0 | 0 | cellular response to hydrogen peroxide | 1 |
| GO:2000277 | 5 | 5 | 0 | 0 | positive regulation of oxidative phosphorylation uncoupler activity | 1 |
| GO:0000105 | 4 | 4 | 0 | 0 | histidine biosynthetic process | 1 |
| GO:0000811 | 4 | 4 | 0 | 0 | GINS complex | 1 |
| GO:0001893 | 4 | 4 | 0 | 0 | maternal placenta development | 1 |
| GO:0004021 | 4 | 4 | 0 | 0 | L-alanine:2-oxoglutarate aminotransferase activity | 1 |
| GO:0004329 | 4 | 4 | 0 | 0 | formate-tetrahydrofolate ligase activity | 1 |
| GO:0004477 | 4 | 4 | 0 | 0 | methenyltetrahydrofolate cyclohydrolase activity | 1 |
| GO:0004731 | 4 | 4 | 0 | 0 | purine-nucleoside phosphorylase activity | 1 |
| GO:0004751 | 4 | 4 | 0 | 0 | ribose-5-phosphate isomerase activity | 1 |
| GO:0008385 | 4 | 4 | 0 | 0 | IkappaB kinase complex | 1 |
| GO:0009052 | 4 | 4 | 0 | 0 | pentose-phosphate shunt. non-oxidative branch | 1 |
| GO:0030099 | 4 | 4 | 0 | 0 | myeloid cell differentiation | 1 |
| GO:0032496 | 4 | 4 | 0 | 0 | response to lipopolysaccharide | 1 |
| GO:0033673 | 4 | 4 | 0 | 0 | negative regulation of kinase activity | 1 |
| GO:0042132 | 4 | 4 | 0 | 0 | fructose 1.6-bisphosphate 1-phosphatase activity | 1 |
| GO:0045968 | 4 | 4 | 0 | 0 | negative regulation of juvenile hormone biosynthetic process | 1 |
| GO:0008200 | 8 | 7 | 2.72E-07 | 1.09E-05 | ion channel inhibitor activity | 0.875 |
| GO:0005811 | 55 | 48 | 4.93E-34 | 2.83E-32 | lipid particle | 0.872727 |
| GO:0034599 | 6 | 5 | 1.19E-05 | 0.000361 | cellular response to oxidative stress | 0.833333 |
| GO:0007005 | 11 | 9 | 5.87E-08 | 2.45E-06 | mitochondrion organization | 0.818182 |
| GO:0008768 | 11 | 9 | 5.87E-08 | 2.45E-06 | UDP-sugar diphosphatase activity | 0.818182 |
| GO:0047936 | 11 | 9 | 5.87E-08 | 2.45E-06 | glucose 1-dehydrogenase [NAD(P)] activity | 0.818182 |
| GO:0006271 | 5 | 4 | 7.89E-05 | 0.001629 | DNA strand elongation involved in DNA replication | 0.8 |
| GO:0007498 | 5 | 4 | 7.89E-05 | 0.001629 | mesoderm development | 0.8 |
| GO:0019900 | 5 | 4 | 7.89E-05 | 0.001629 | kinase binding | 0.8 |
| GO:0045665 | 5 | 4 | 7.89E-05 | 0.001629 | negative regulation of neuron differentiation | 0.8 |
| GO:0000786 | 104 | 83 | 2.21E-50 | 1.42E-48 | nucleosome | 0.798077 |
| GO:0006334 | 109 | 83 | 2.21E-47 | 1.38E-45 | nucleosome assembly | 0.761468 |
| GO:0006154 | 8 | 6 | 1.25E-05 | 0.000361 | adenosine catabolic process | 0.75 |
| GO:0031685 | 8 | 6 | 1.25E-05 | 0.000361 | adenosine receptor binding | 0.75 |
| GO:0043394 | 8 | 6 | 1.25E-05 | 0.000361 | proteoglycan binding | 0.75 |
| GO:0045088 | 8 | 6 | 1.25E-05 | 0.000361 | regulation of innate immune response | 0.75 |
| GO:0051635 | 8 | 6 | 1.25E-05 | 0.000361 | bacterial cell surface binding | 0.75 |
| GO:0006111 | 4 | 3 | 0.000523 | 0.006858 | regulation of gluconeogenesis | 0.75 |
| GO:0006898 | 4 | 3 | 0.000523 | 0.006858 | receptor-mediated endocytosis | 0.75 |
| GO:0007629 | 4 | 3 | 0.000523 | 0.006858 | flight behavior | 0.75 |
| GO:0010259 | 4 | 3 | 0.000523 | 0.006858 | multicellular organismal aging | 0.75 |
| GO:0016594 | 4 | 3 | 0.000523 | 0.006858 | glycine binding | 0.75 |
| GO:0017148 | 4 | 3 | 0.000523 | 0.006858 | negative regulation of translation | 0.75 |
| GO:0017174 | 4 | 3 | 0.000523 | 0.006858 | glycine N-methyltransferase activity | 0.75 |
| GO:0019216 | 4 | 3 | 0.000523 | 0.006858 | regulation of lipid metabolic process | 0.75 |
| GO:0046500 | 4 | 3 | 0.000523 | 0.006858 | S-adenosylmethionine metabolic process | 0.75 |
| GO:0004611 | 11 | 8 | 1.68E-06 | 5.87E-05 | phosphoenolpyruvate carboxykinase activity | 0.727273 |
| GO:0017076 | 11 | 8 | 1.68E-06 | 5.87E-05 | purine nucleotide binding | 0.727273 |
| GO:0047964 | 11 | 8 | 1.68E-06 | 5.87E-05 | glyoxylate reductase activity | 0.727273 |
| GO:0008453 | 7 | 5 | 7.27E-05 | 0.001576 | alanine-glyoxylate transaminase activity | 0.714286 |
| GO:0009755 | 7 | 5 | 7.27E-05 | 0.001576 | hormone-mediated signaling pathway | 0.714286 |
| GO:0050727 | 7 | 5 | 7.27E-05 | 0.001576 | regulation of inflammatory response | 0.714286 |
| GO:0050830 | 15 | 10 | 7.03E-07 | 2.62E-05 | defense response to Gram-positive bacterium | 0.666667 |
| GO:0008308 | 9 | 6 | 4.88E-05 | 0.001104 | voltage-gated anion channel activity | 0.666667 |
| GO:0015288 | 9 | 6 | 4.88E-05 | 0.001104 | porin activity | 0.666667 |
| GO:0044070 | 9 | 6 | 4.88E-05 | 0.001104 | regulation of anion transport | 0.666667 |
| GO:0046930 | 9 | 6 | 4.88E-05 | 0.001104 | pore complex | 0.666667 |
| GO:0002098 | 6 | 4 | 0.000414 | 0.005787 | tRNA wobble uridine modification | 0.666667 |
| GO:0003980 | 6 | 4 | 0.000414 | 0.005787 | UDP-glucose:glycoprotein glucosyltransferase activity | 0.666667 |
| GO:0004034 | 6 | 4 | 0.000414 | 0.005787 | aldose 1-epimerase activity | 0.666667 |
| GO:0004351 | 6 | 4 | 0.000414 | 0.005787 | glutamate decarboxylase activity | 0.666667 |
| GO:0007034 | 6 | 4 | 0.000414 | 0.005787 | vacuolar transport | 0.666667 |
| GO:0016679 | 6 | 4 | 0.000414 | 0.005787 | oxidoreductase activity. acting on diphenols and related substances as donors | 0.666667 |
| GO:0019318 | 6 | 4 | 0.000414 | 0.005787 | hexose metabolic process | 0.666667 |
| GO:0030904 | 6 | 4 | 0.000414 | 0.005787 | retromer complex | 0.666667 |
| GO:0034227 | 6 | 4 | 0.000414 | 0.005787 | tRNA thio-modification | 0.666667 |
| GO:0034614 | 6 | 4 | 0.000414 | 0.005787 | cellular response to reactive oxygen species | 0.666667 |
| GO:0045900 | 6 | 4 | 0.000414 | 0.005787 | negative regulation of translational elongation | 0.666667 |
| GO:0050253 | 6 | 4 | 0.000414 | 0.005787 | retinyl-palmitate esterase activity | 0.666667 |
| GO:0035267 | 34 | 22 | 6.51E-12 | 3.08E-10 | NuA4 histone acetyltransferase complex | 0.647059 |
| GO:0030435 | 11 | 7 | 2.91E-05 | 0.000764 | sporulation resulting in formation of a cellular spore | 0.636364 |
| GO:0045980 | 8 | 5 | 0.000253 | 0.004172 | negative regulation of nucleotide metabolic process | 0.625 |
| GO:0005786 | 13 | 8 | 1.64E-05 | 0.000464 | signal recognition particle. endoplasmic reticulum targeting | 0.615385 |
| GO:0009166 | 20 | 12 | 5.73E-07 | 2.18E-05 | nucleotide catabolic process | 0.6 |
| GO:0005665 | 10 | 6 | 0.000141 | 0.002505 | DNA-directed RNA polymerase II. core complex | 0.6 |
| GO:0031409 | 10 | 6 | 0.000141 | 0.002505 | pigment binding | 0.6 |
| GO:0000188 | 5 | 3 | 0.002297 | 0.022531 | inactivation of MAPK activity | 0.6 |
| GO:0000989 | 5 | 3 | 0.002297 | 0.022531 | transcription factor binding transcription factor activity | 0.6 |
| GO:0001755 | 5 | 3 | 0.002297 | 0.022531 | neural crest cell migration | 0.6 |
| GO:0001772 | 5 | 3 | 0.002297 | 0.022531 | immunological synapse | 0.6 |
| GO:0004735 | 5 | 3 | 0.002297 | 0.022531 | pyrroline-5-carboxylate reductase activity | 0.6 |
| GO:0006555 | 5 | 3 | 0.002297 | 0.022531 | methionine metabolic process | 0.6 |
| GO:0007368 | 5 | 3 | 0.002297 | 0.022531 | determination of left/right symmetry | 0.6 |
| GO:0033549 | 5 | 3 | 0.002297 | 0.022531 | MAP kinase phosphatase activity | 0.6 |
| GO:0043409 | 5 | 3 | 0.002297 | 0.022531 | negative regulation of MAPK cascade | 0.6 |
| GO:0046329 | 5 | 3 | 0.002297 | 0.022531 | negative regulation of JNK cascade | 0.6 |
| GO:0050860 | 5 | 3 | 0.002297 | 0.022531 | negative regulation of T cell receptor signaling pathway | 0.6 |
| GO:0050868 | 5 | 3 | 0.002297 | 0.022531 | negative regulation of T cell activation | 0.6 |
| GO:0070373 | 5 | 3 | 0.002297 | 0.022531 | negative regulation of ERK1 and ERK2 cascade | 0.6 |
| GO:0008083 | 41 | 24 | 2.42E-11 | 1.12E-09 | growth factor activity | 0.585366 |
| GO:0016630 | 12 | 7 | 7.57E-05 | 0.001626 | protochlorophyllide reductase activity | 0.583333 |
| GO:0004316 | 21 | 12 | 1.30E-06 | 4.75E-05 | 3-oxoacyl-[acyl-carrier-protein] reductase (NADPH) activity | 0.571429 |
| GO:0004341 | 14 | 8 | 3.97E-05 | 0.000992 | gluconolactonase activity | 0.571429 |
| GO:0019853 | 14 | 8 | 3.97E-05 | 0.000992 | L-ascorbic acid biosynthetic process | 0.571429 |
| GO:0050848 | 14 | 8 | 3.97E-05 | 0.000992 | regulation of calcium-mediated signaling | 0.571429 |
| GO:0003837 | 7 | 4 | 0.001268 | 0.014411 | beta-ureidopropionase activity | 0.571429 |
| GO:0007389 | 7 | 4 | 0.001268 | 0.014411 | pattern specification process | 0.571429 |
| GO:0016763 | 7 | 4 | 0.001268 | 0.014411 | transferase activity. transferring pentosyl groups | 0.571429 |
| GO:0021794 | 7 | 4 | 0.001268 | 0.014411 | thalamus development | 0.571429 |
| GO:0030942 | 7 | 4 | 0.001268 | 0.014411 | endoplasmic reticulum signal peptide binding | 0.571429 |
| GO:0019395 | 9 | 5 | 0.000662 | 0.008227 | fatty acid oxidation | 0.555556 |
| GO:0001505 | 11 | 6 | 0.000338 | 0.005204 | regulation of neurotransmitter levels | 0.545455 |
| GO:0005932 | 11 | 6 | 0.000338 | 0.005204 | microtubule basal body | 0.545455 |
| GO:0008503 | 11 | 6 | 0.000338 | 0.005204 | benzodiazepine receptor activity | 0.545455 |
| GO:0030594 | 11 | 6 | 0.000338 | 0.005204 | neurotransmitter receptor activity | 0.545455 |
| GO:0030658 | 11 | 6 | 0.000338 | 0.005204 | transport vesicle membrane | 0.545455 |
| GO:0008312 | 15 | 8 | 8.58E-05 | 0.001705 | 7S RNA binding | 0.533333 |
| GO:0009168 | 15 | 8 | 8.58E-05 | 0.001705 | purine ribonucleoside monophosphate biosynthetic process | 0.533333 |
| GO:0019239 | 15 | 8 | 8.58E-05 | 0.001705 | deaminase activity | 0.533333 |
| GO:0048500 | 15 | 8 | 8.58E-05 | 0.001705 | signal recognition particle | 0.533333 |
| GO:0006954 | 17 | 9 | 4.30E-05 | 0.001028 | inflammatory response | 0.529412 |
| GO:0006874 | 19 | 10 | 2.15E-05 | 0.000595 | cellular calcium ion homeostasis | 0.526316 |
| GO:0045735 | 21 | 11 | 1.08E-05 | 0.000347 | nutrient reservoir activity | 0.52381 |
| GO:0006094 | 23 | 12 | 5.40E-06 | 0.000179 | gluconeogenesis | 0.521739 |
| GO:0042742 | 139 | 72 | 6.74E-25 | 3.67E-23 | defense response to bacterium | 0.517986 |
| GO:0005213 | 72 | 36 | 6.36E-13 | 3.07E-11 | structural constituent of chorion | 0.5 |
| GO:0007304 | 72 | 36 | 6.36E-13 | 3.07E-11 | chorion-containing eggshell formation | 0.5 |
| GO:0042600 | 72 | 36 | 6.36E-13 | 3.07E-11 | chorion | 0.5 |
| GO:0006693 | 16 | 8 | 0.00017 | 0.002934 | prostaglandin metabolic process | 0.5 |
| GO:0004032 | 14 | 7 | 0.000345 | 0.005283 | alditol:NADP+ 1-oxidoreductase activity | 0.5 |
| GO:0030151 | 12 | 6 | 0.000705 | 0.008711 | molybdenum ion binding | 0.5 |
| GO:0016671 | 10 | 5 | 0.001442 | 0.015724 | oxidoreductase activity. acting on a sulfur group of donors. disulfide as acceptor | 0.5 |
| GO:0004348 | 8 | 4 | 0.002958 | 0.027584 | glucosylceramidase activity | 0.5 |
| GO:0004470 | 8 | 4 | 0.002958 | 0.027584 | malic enzyme activity | 0.5 |
| GO:0004473 | 8 | 4 | 0.002958 | 0.027584 | malate dehydrogenase (oxaloacetate-decarboxylating) (NADP+) activity | 0.5 |
| GO:0004488 | 8 | 4 | 0.002958 | 0.027584 | methylenetetrahydrofolate dehydrogenase (NADP+) activity | 0.5 |
| GO:0004596 | 8 | 4 | 0.002958 | 0.027584 | peptide alpha-N-acetyltransferase activity | 0.5 |
| GO:0016619 | 8 | 4 | 0.002958 | 0.027584 | malate dehydrogenase (oxaloacetate-decarboxylating) activity | 0.5 |
| GO:0000178 | 6 | 3 | 0.006063 | 0.05115 | exosome (RNase complex) | 0.5 |
| GO:0003884 | 6 | 3 | 0.006063 | 0.05115 | D-amino-acid oxidase activity | 0.5 |
| GO:0004152 | 6 | 3 | 0.006063 | 0.05115 | dihydroorotate dehydrogenase activity | 0.5 |
| GO:0004158 | 6 | 3 | 0.006063 | 0.05115 | dihydroorotate oxidase activity | 0.5 |
| GO:0008624 | 6 | 3 | 0.006063 | 0.05115 | induction of apoptosis by extracellular signals | 0.5 |
| GO:0019367 | 6 | 3 | 0.006063 | 0.05115 | fatty acid elongation. saturated fatty acid | 0.5 |
| GO:0019464 | 6 | 3 | 0.006063 | 0.05115 | glycine decarboxylation via glycine cleavage system | 0.5 |
| GO:0042761 | 6 | 3 | 0.006063 | 0.05115 | very long-chain fatty acid biosynthetic process | 0.5 |
| GO:0080008 | 6 | 3 | 0.006063 | 0.05115 | Cul4-RING ubiquitin ligase complex | 0.5 |
| GO:0000331 | 4 | 2 | 0.012264 | 0.077892 | contractile vacuole | 0.5 |
| GO:0001921 | 4 | 2 | 0.012264 | 0.077892 | positive regulation of receptor recycling | 0.5 |
| GO:0001974 | 4 | 2 | 0.012264 | 0.077892 | blood vessel remodeling | 0.5 |
| GO:0004500 | 4 | 2 | 0.012264 | 0.077892 | dopamine beta-monooxygenase activity | 0.5 |
| GO:0004815 | 4 | 2 | 0.012264 | 0.077892 | aspartate-tRNA ligase activity | 0.5 |
| GO:0004818 | 4 | 2 | 0.012264 | 0.077892 | glutamate-tRNA ligase activity | 0.5 |
| GO:0004946 | 4 | 2 | 0.012264 | 0.077892 | bombesin receptor activity | 0.5 |
| GO:0004995 | 4 | 2 | 0.012264 | 0.077892 | tachykinin receptor activity | 0.5 |
| GO:0005283 | 4 | 2 | 0.012264 | 0.077892 | sodium:amino acid symporter activity | 0.5 |
| GO:0005879 | 4 | 2 | 0.012264 | 0.077892 | axonemal microtubule | 0.5 |
| GO:0006264 | 4 | 2 | 0.012264 | 0.077892 | mitochondrial DNA replication | 0.5 |
| GO:0006424 | 4 | 2 | 0.012264 | 0.077892 | glutamyl-tRNA aminoacylation | 0.5 |
| GO:0006584 | 4 | 2 | 0.012264 | 0.077892 | catecholamine metabolic process | 0.5 |
| GO:0006857 | 4 | 2 | 0.012264 | 0.077892 | oligopeptide transport | 0.5 |
| GO:0007217 | 4 | 2 | 0.012264 | 0.077892 | tachykinin receptor signaling pathway | 0.5 |
| GO:0007613 | 4 | 2 | 0.012264 | 0.077892 | memory | 0.5 |
| GO:0008191 | 4 | 2 | 0.012264 | 0.077892 | metalloendopeptidase inhibitor activity | 0.5 |
| GO:0008306 | 4 | 2 | 0.012264 | 0.077892 | associative learning | 0.5 |
| GO:0010814 | 4 | 2 | 0.012264 | 0.077892 | substance P catabolic process | 0.5 |
| GO:0010815 | 4 | 2 | 0.012264 | 0.077892 | bradykinin catabolic process | 0.5 |
| GO:0010816 | 4 | 2 | 0.012264 | 0.077892 | calcitonin catabolic process | 0.5 |
| GO:0018169 | 4 | 2 | 0.012264 | 0.077892 | ribosomal S6-glutamic acid ligase activity | 0.5 |
| GO:0019799 | 4 | 2 | 0.012264 | 0.077892 | tubulin N-acetyltransferase activity | 0.5 |
| GO:0021539 | 4 | 2 | 0.012264 | 0.077892 | subthalamus development | 0.5 |
| GO:0021548 | 4 | 2 | 0.012264 | 0.077892 | pons development | 0.5 |
| GO:0021766 | 4 | 2 | 0.012264 | 0.077892 | hippocampus development | 0.5 |
| GO:0021860 | 4 | 2 | 0.012264 | 0.077892 | pyramidal neuron development | 0.5 |
| GO:0030901 | 4 | 2 | 0.012264 | 0.077892 | midbrain development | 0.5 |
| GO:0031989 | 4 | 2 | 0.012264 | 0.077892 | bombesin receptor signaling pathway | 0.5 |
| GO:0033093 | 4 | 2 | 0.012264 | 0.077892 | Weibel-Palade body | 0.5 |
| GO:0034959 | 4 | 2 | 0.012264 | 0.077892 | endothelin maturation | 0.5 |
| GO:0035589 | 4 | 2 | 0.012264 | 0.077892 | G-protein coupled purinergic nucleotide receptor signaling pathway | 0.5 |
| GO:0042447 | 4 | 2 | 0.012264 | 0.077892 | hormone catabolic process | 0.5 |
| GO:0042733 | 4 | 2 | 0.012264 | 0.077892 | embryonic digit morphogenesis | 0.5 |
| GO:0043484 | 4 | 2 | 0.012264 | 0.077892 | regulation of RNA splicing | 0.5 |
| GO:0043583 | 4 | 2 | 0.012264 | 0.077892 | ear development | 0.5 |
| GO:0045028 | 4 | 2 | 0.012264 | 0.077892 | G-protein coupled purinergic nucleotide receptor activity | 0.5 |
| GO:0047305 | 4 | 2 | 0.012264 | 0.077892 | (R)-3-amino-2-methylpropionate-pyruvate transaminase activity | 0.5 |
| GO:0048149 | 4 | 2 | 0.012264 | 0.077892 | behavioral response to ethanol | 0.5 |
| GO:0050900 | 4 | 2 | 0.012264 | 0.077892 | leukocyte migration | 0.5 |
| GO:0060037 | 4 | 2 | 0.012264 | 0.077892 | pharyngeal system development | 0.5 |
| GO:0061136 | 4 | 2 | 0.012264 | 0.077892 | regulation of proteasomal protein catabolic process | 0.5 |
| GO:0070740 | 4 | 2 | 0.012264 | 0.077892 | tubulin-glutamic acid ligase activity | 0.5 |
| GO:0005125 | 25 | 12 | 1.82E-05 | 0.00051 | cytokine activity | 0.48 |
| GO:0004022 | 19 | 9 | 0.000153 | 0.002683 | alcohol dehydrogenase (NAD) activity | 0.473684 |
| GO:0004857 | 15 | 7 | 0.000642 | 0.008077 | enzyme inhibitor activity | 0.466667 |
| GO:0008408 | 15 | 7 | 0.000642 | 0.008077 | 3'-5' exonuclease activity | 0.466667 |
| GO:0043086 | 15 | 7 | 0.000642 | 0.008077 | negative regulation of catalytic activity | 0.466667 |
| GO:0042302 | 351 | 162 | 5.04E-45 | 3.06E-43 | structural constituent of cuticle | 0.461538 |
| GO:0006820 | 13 | 6 | 0.001328 | 0.014858 | anion transport | 0.461538 |
| GO:0007214 | 13 | 6 | 0.001328 | 0.014858 | gamma-aminobutyric acid signaling pathway | 0.461538 |
| GO:0005694 | 193 | 89 | 1.75E-25 | 9.79E-24 | chromosome | 0.46114 |
| GO:0005762 | 11 | 5 | 0.002768 | 0.026745 | mitochondrial large ribosomal subunit | 0.454545 |
| GO:0001525 | 20 | 9 | 0.000264 | 0.004285 | angiogenesis | 0.45 |
| GO:0008253 | 20 | 9 | 0.000264 | 0.004285 | 5'-nucleotidase activity | 0.45 |
| GO:0045087 | 181 | 81 | 2.58E-22 | 1.34E-20 | innate immune response | 0.447514 |
| GO:0006614 | 18 | 8 | 0.000541 | 0.006971 | SRP-dependent cotranslational protein targeting to membrane | 0.444444 |
| GO:0032781 | 18 | 8 | 0.000541 | 0.006971 | positive regulation of ATPase activity | 0.444444 |
| GO:0051920 | 18 | 8 | 0.000541 | 0.006971 | peroxiredoxin activity | 0.444444 |
| GO:0016051 | 9 | 4 | 0.005829 | 0.05115 | carbohydrate biosynthetic process | 0.444444 |
| GO:0042542 | 9 | 4 | 0.005829 | 0.05115 | response to hydrogen peroxide | 0.444444 |
| GO:0005179 | 75 | 33 | 4.39E-10 | 1.98E-08 | hormone activity | 0.44 |
| GO:0018833 | 25 | 11 | 0.000106 | 0.001952 | DDT-dehydrochlorinase activity | 0.44 |
| GO:0004890 | 16 | 7 | 0.001114 | 0.013089 | GABA-A receptor activity | 0.4375 |
| GO:0007369 | 16 | 7 | 0.001114 | 0.013089 | gastrulation | 0.4375 |
| GO:0005344 | 30 | 13 | 4.17E-05 | 0.001008 | oxygen transporter activity | 0.433333 |
| GO:0050790 | 30 | 13 | 4.17E-05 | 0.001008 | regulation of catalytic activity | 0.433333 |
| GO:0004000 | 14 | 6 | 0.00231 | 0.022531 | adenosine deaminase activity | 0.428571 |
| GO:0009086 | 14 | 6 | 0.00231 | 0.022531 | methionine biosynthetic process | 0.428571 |
| GO:0000104 | 7 | 3 | 0.012457 | 0.077892 | succinate dehydrogenase activity | 0.428571 |
| GO:0004053 | 7 | 3 | 0.012457 | 0.077892 | arginase activity | 0.428571 |
| GO:0004957 | 7 | 3 | 0.012457 | 0.077892 | prostaglandin E receptor activity | 0.428571 |
| GO:0005746 | 7 | 3 | 0.012457 | 0.077892 | mitochondrial respiratory chain | 0.428571 |
| GO:0005798 | 7 | 3 | 0.012457 | 0.077892 | Golgi-associated vesicle | 0.428571 |
| GO:0006401 | 7 | 3 | 0.012457 | 0.077892 | RNA catabolic process | 0.428571 |
| GO:0006525 | 7 | 3 | 0.012457 | 0.077892 | arginine metabolic process | 0.428571 |
| GO:0006572 | 7 | 3 | 0.012457 | 0.077892 | tyrosine catabolic process | 0.428571 |
| GO:0007565 | 7 | 3 | 0.012457 | 0.077892 | female pregnancy | 0.428571 |
| GO:0007567 | 7 | 3 | 0.012457 | 0.077892 | parturition | 0.428571 |
| GO:0008853 | 7 | 3 | 0.012457 | 0.077892 | exodeoxyribonuclease III activity | 0.428571 |
| GO:0016404 | 7 | 3 | 0.012457 | 0.077892 | 15-hydroxyprostaglandin dehydrogenase (NAD+) activity | 0.428571 |
| GO:0016641 | 7 | 3 | 0.012457 | 0.077892 | oxidoreductase activity. acting on the CH-NH2 group of donors. oxygen as acceptor | 0.428571 |
| GO:0019432 | 7 | 3 | 0.012457 | 0.077892 | triglyceride biosynthetic process | 0.428571 |
| GO:0042832 | 7 | 3 | 0.012457 | 0.077892 | defense response to protozoan | 0.428571 |
| GO:0045666 | 7 | 3 | 0.012457 | 0.077892 | positive regulation of neuron differentiation | 0.428571 |
| GO:0045786 | 7 | 3 | 0.012457 | 0.077892 | negative regulation of cell cycle | 0.428571 |
| GO:0046592 | 7 | 3 | 0.012457 | 0.077892 | polyamine oxidase activity | 0.428571 |
| GO:0005315 | 33 | 14 | 3.27E-05 | 0.000849 | inorganic phosphate transmembrane transporter activity | 0.424242 |
| GO:0004806 | 19 | 8 | 0.000891 | 0.010881 | triglyceride lipase activity | 0.421053 |
| GO:0003997 | 12 | 5 | 0.00483 | 0.043149 | acyl-CoA oxidase activity | 0.416667 |
| GO:0032784 | 12 | 5 | 0.00483 | 0.043149 | regulation of DNA-dependent transcription. elongation | 0.416667 |
| GO:0072593 | 12 | 5 | 0.00483 | 0.043149 | reactive oxygen species metabolic process | 0.416667 |
| GO:0005212 | 41 | 17 | 9.83E-06 | 0.000322 | structural constituent of eye lens | 0.414634 |
| GO:0006817 | 34 | 14 | 5.04E-05 | 0.001129 | phosphate ion transport | 0.411765 |
| GO:0015114 | 32 | 13 | 0.0001 | 0.001887 | phosphate ion transmembrane transporter activity | 0.40625 |
| GO:0015671 | 32 | 13 | 0.0001 | 0.001887 | oxygen transport | 0.40625 |
| GO:0035435 | 32 | 13 | 0.0001 | 0.001887 | phosphate ion transmembrane transport | 0.40625 |
| GO:0022627 | 20 | 8 | 0.001403 | 0.015386 | cytosolic small ribosomal subunit | 0.4 |
| GO:0004835 | 15 | 6 | 0.00377 | 0.034697 | tubulin-tyrosine ligase activity | 0.4 |
| GO:0006833 | 15 | 6 | 0.00377 | 0.034697 | water transport | 0.4 |
| GO:0004550 | 10 | 4 | 0.010215 | 0.077892 | nucleoside diphosphate kinase activity | 0.4 |
| GO:0006165 | 10 | 4 | 0.010215 | 0.077892 | nucleoside diphosphate phosphorylation | 0.4 |
| GO:0006183 | 10 | 4 | 0.010215 | 0.077892 | GTP biosynthetic process | 0.4 |
| GO:0006228 | 10 | 4 | 0.010215 | 0.077892 | UTP biosynthetic process | 0.4 |
| GO:0006241 | 10 | 4 | 0.010215 | 0.077892 | CTP biosynthetic process | 0.4 |
| GO:0051881 | 10 | 4 | 0.010215 | 0.077892 | regulation of mitochondrial membrane potential | 0.4 |
| GO:0000015 | 5 | 2 | 0.027214 | 0.144644 | phosphopyruvate hydratase complex | 0.4 |
| GO:0000118 | 5 | 2 | 0.027214 | 0.144644 | histone deacetylase complex | 0.4 |
| GO:0000214 | 5 | 2 | 0.027214 | 0.144644 | tRNA-intron endonuclease complex | 0.4 |
| GO:0004634 | 5 | 2 | 0.027214 | 0.144644 | phosphopyruvate hydratase activity | 0.4 |
| GO:0008173 | 5 | 2 | 0.027214 | 0.144644 | RNA methyltransferase activity | 0.4 |
| GO:0009434 | 5 | 2 | 0.027214 | 0.144644 | microtubule-based flagellum | 0.4 |
| GO:0009653 | 5 | 2 | 0.027214 | 0.144644 | anatomical structure morphogenesis | 0.4 |
| GO:0016486 | 5 | 2 | 0.027214 | 0.144644 | peptide hormone processing | 0.4 |
| GO:0021680 | 5 | 2 | 0.027214 | 0.144644 | cerebellar Purkinje cell layer development | 0.4 |
| GO:0021854 | 5 | 2 | 0.027214 | 0.144644 | hypothalamus development | 0.4 |
| GO:0030137 | 5 | 2 | 0.027214 | 0.144644 | COPI-coated vesicle | 0.4 |
| GO:0032225 | 5 | 2 | 0.027214 | 0.144644 | regulation of synaptic transmission. dopaminergic | 0.4 |
| GO:0042255 | 5 | 2 | 0.027214 | 0.144644 | ribosome assembly | 0.4 |
| GO:0042597 | 5 | 2 | 0.027214 | 0.144644 | periplasmic space | 0.4 |
| GO:0048793 | 5 | 2 | 0.027214 | 0.144644 | pronephros development | 0.4 |
| GO:0050220 | 5 | 2 | 0.027214 | 0.144644 | prostaglandin-E synthase activity | 0.4 |
| GO:0050614 | 5 | 2 | 0.027214 | 0.144644 | delta24-sterol reductase activity | 0.4 |
| GO:0030663 | 23 | 9 | 0.001059 | 0.012572 | COPI coated vesicle membrane | 0.391304 |
| GO:0031640 | 23 | 9 | 0.001059 | 0.012572 | killing of cells of other organism | 0.391304 |
| GO:0050832 | 23 | 9 | 0.001059 | 0.012572 | defense response to fungus | 0.391304 |
| GO:0009405 | 18 | 7 | 0.002856 | 0.027353 | pathogenesis | 0.388889 |
| GO:0046477 | 18 | 7 | 0.002856 | 0.027353 | glycosylceramide catabolic process | 0.388889 |
| GO:0051092 | 13 | 5 | 0.007833 | 0.063802 | positive regulation of NF-kappaB transcription factor activity | 0.384615 |
| GO:0007596 | 21 | 8 | 0.002129 | 0.022402 | blood coagulation | 0.380952 |
| GO:0008422 | 21 | 8 | 0.002129 | 0.022402 | beta-glucosidase activity | 0.380952 |
| GO:0042645 | 21 | 8 | 0.002129 | 0.022402 | mitochondrial nucleoid | 0.380952 |
| GO:0030234 | 24 | 9 | 0.001571 | 0.01695 | enzyme regulator activity | 0.375 |
| GO:0002756 | 8 | 3 | 0.021956 | 0.127534 | MyD88-independent toll-like receptor signaling pathway | 0.375 |
| GO:0003998 | 8 | 3 | 0.021956 | 0.127534 | acylphosphatase activity | 0.375 |
| GO:0004517 | 8 | 3 | 0.021956 | 0.127534 | nitric-oxide synthase activity | 0.375 |
| GO:0004771 | 8 | 3 | 0.021956 | 0.127534 | sterol esterase activity | 0.375 |
| GO:0004794 | 8 | 3 | 0.021956 | 0.127534 | L-threonine ammonia-lyase activity | 0.375 |
| GO:0005542 | 8 | 3 | 0.021956 | 0.127534 | folic acid binding | 0.375 |
| GO:0005960 | 8 | 3 | 0.021956 | 0.127534 | glycine cleavage complex | 0.375 |
| GO:0006809 | 8 | 3 | 0.021956 | 0.127534 | nitric oxide biosynthetic process | 0.375 |
| GO:0014069 | 8 | 3 | 0.021956 | 0.127534 | postsynaptic density | 0.375 |
| GO:0016813 | 8 | 3 | 0.021956 | 0.127534 | hydrolase activity. acting on carbon-nitrogen (but not peptide) bonds. in linear amidines | 0.375 |
| GO:0016847 | 8 | 3 | 0.021956 | 0.127534 | 1-aminocyclopropane-1-carboxylate synthase activity | 0.375 |
| GO:0030133 | 8 | 3 | 0.021956 | 0.127534 | transport vesicle | 0.375 |
| GO:0042218 | 8 | 3 | 0.021956 | 0.127534 | 1-aminocyclopropane-1-carboxylate biosynthetic process | 0.375 |
| GO:0004181 | 43 | 16 | 8.77E-05 | 0.001724 | metallocarboxypeptidase activity | 0.372093 |
| GO:0004499 | 11 | 4 | 0.016422 | 0.100614 | N.N-dimethylaniline monooxygenase activity | 0.363636 |
| GO:0042578 | 11 | 4 | 0.016422 | 0.100614 | phosphoric ester hydrolase activity | 0.363636 |
| GO:0005328 | 25 | 9 | 0.002266 | 0.022531 | neurotransmitter:sodium symporter activity | 0.36 |
| GO:0005615 | 369 | 132 | 1.13E-23 | 6.00E-22 | extracellular space | 0.357724 |
| GO:0016810 | 48 | 17 | 0.000123 | 0.002257 | hydrolase activity. acting on carbon-nitrogen (but not peptide) bonds | 0.354167 |
| GO:0008201 | 17 | 6 | 0.008641 | 0.069158 | heparin binding | 0.352941 |
| GO:0019538 | 17 | 6 | 0.008641 | 0.069158 | protein metabolic process | 0.352941 |
| GO:0030285 | 17 | 6 | 0.008641 | 0.069158 | integral to synaptic vesicle membrane | 0.352941 |
| GO:0005765 | 57 | 20 | 4.68E-05 | 0.001104 | lysosomal membrane | 0.350877 |
| GO:0042493 | 23 | 8 | 0.004451 | 0.040439 | response to drug | 0.347826 |
| GO:0016758 | 127 | 44 | 1.18E-08 | 5.21E-07 | transferase activity. transferring hexosyl groups | 0.346457 |
| GO:0008121 | 26 | 9 | 0.003189 | 0.029609 | ubiquinol-cytochrome-c reductase activity | 0.346154 |
| GO:0016831 | 35 | 12 | 0.001175 | 0.013722 | carboxy-lyase activity | 0.342857 |
| GO:0003954 | 48 | 16 | 0.000416 | 0.005787 | NADH dehydrogenase activity | 0.333333 |
| GO:0004565 | 21 | 7 | 0.008718 | 0.069158 | beta-galactosidase activity | 0.333333 |
| GO:0043154 | 21 | 7 | 0.008718 | 0.069158 | negative regulation of cysteine-type endopeptidase activity involved in apoptotic process | 0.333333 |
| GO:0006952 | 18 | 6 | 0.012323 | 0.077892 | defense response | 0.333333 |
| GO:0007160 | 15 | 5 | 0.017444 | 0.10596 | cell-matrix adhesion | 0.333333 |
| GO:0042593 | 15 | 5 | 0.017444 | 0.10596 | glucose homeostasis | 0.333333 |
| GO:0008484 | 12 | 4 | 0.024702 | 0.137836 | sulfuric ester hydrolase activity | 0.333333 |
| GO:0001516 | 9 | 3 | 0.034857 | 0.178139 | prostaglandin biosynthetic process | 0.333333 |
| GO:0005749 | 9 | 3 | 0.034857 | 0.178139 | mitochondrial respiratory chain complex II | 0.333333 |
| GO:0005853 | 9 | 3 | 0.034857 | 0.178139 | eukaryotic translation elongation factor 1 complex | 0.333333 |
| GO:0006222 | 9 | 3 | 0.034857 | 0.178139 | UMP biosynthetic process | 0.333333 |
| GO:0007040 | 9 | 3 | 0.034857 | 0.178139 | lysosome organization | 0.333333 |
| GO:0042765 | 9 | 3 | 0.034857 | 0.178139 | GPI-anchor transamidase complex | 0.333333 |
| GO:0044419 | 9 | 3 | 0.034857 | 0.178139 | interspecies interaction between organisms | 0.333333 |
| GO:0051259 | 9 | 3 | 0.034857 | 0.178139 | protein oligomerization | 0.333333 |
| GO:0051781 | 9 | 3 | 0.034857 | 0.178139 | positive regulation of cell division | 0.333333 |
| GO:0004045 | 6 | 2 | 0.048366 | 0.231069 | aminoacyl-tRNA hydrolase activity | 0.333333 |
| GO:0005923 | 6 | 2 | 0.048366 | 0.231069 | tight junction | 0.333333 |
| GO:0005997 | 6 | 2 | 0.048366 | 0.231069 | xylulose metabolic process | 0.333333 |
| GO:0006388 | 6 | 2 | 0.048366 | 0.231069 | tRNA splicing. via endonucleolytic cleavage and ligation | 0.333333 |
| GO:0006569 | 6 | 2 | 0.048366 | 0.231069 | tryptophan catabolic process | 0.333333 |
| GO:0006835 | 6 | 2 | 0.048366 | 0.231069 | dicarboxylic acid transport | 0.333333 |
| GO:0007367 | 6 | 2 | 0.048366 | 0.231069 | segment polarity determination | 0.333333 |
| GO:0009888 | 6 | 2 | 0.048366 | 0.231069 | tissue development | 0.333333 |
| GO:0015128 | 6 | 2 | 0.048366 | 0.231069 | gluconate transmembrane transporter activity | 0.333333 |
| GO:0016715 | 6 | 2 | 0.048366 | 0.231069 | oxidoreductase activity. acting on paired donors. with incorporation or reduction of molecular oxygen. reduced ascorbate as one donor. and incorporation of one atom of oxygen | 0.333333 |
| GO:0017137 | 6 | 2 | 0.048366 | 0.231069 | Rab GTPase binding | 0.333333 |
| GO:0017153 | 6 | 2 | 0.048366 | 0.231069 | sodium:dicarboxylate symporter activity | 0.333333 |
| GO:0018095 | 6 | 2 | 0.048366 | 0.231069 | protein polyglutamylation | 0.333333 |
| GO:0033014 | 6 | 2 | 0.048366 | 0.231069 | tetrapyrrole biosynthetic process | 0.333333 |
| GO:0050038 | 6 | 2 | 0.048366 | 0.231069 | L-xylulose reductase (NADP+) activity | 0.333333 |
| GO:0060027 | 6 | 2 | 0.048366 | 0.231069 | convergent extension involved in gastrulation | 0.333333 |
| GO:0070330 | 115 | 38 | 4.06E-07 | 1.57E-05 | aromatase activity | 0.330435 |
| GO:0005747 | 76 | 25 | 2.80E-05 | 0.000744 | mitochondrial respiratory chain complex I | 0.328947 |
| GO:0006836 | 46 | 15 | 0.000767 | 0.009427 | neurotransmitter transport | 0.326087 |
| GO:0003995 | 31 | 10 | 0.004186 | 0.038197 | acyl-CoA dehydrogenase activity | 0.322581 |
| GO:0015293 | 90 | 29 | 1.26E-05 | 0.000361 | symporter activity | 0.322222 |
| GO:0006030 | 88 | 28 | 2.23E-05 | 0.0006 | chitin metabolic process | 0.318182 |
| GO:0008061 | 88 | 28 | 2.23E-05 | 0.0006 | chitin binding | 0.318182 |
| GO:0004298 | 44 | 14 | 0.001404 | 0.015386 | threonine-type endopeptidase activity | 0.318182 |
| GO:0005839 | 44 | 14 | 0.001404 | 0.015386 | proteasome core complex | 0.318182 |
| GO:0051603 | 44 | 14 | 0.001404 | 0.015386 | proteolysis involved in cellular protein catabolic process | 0.318182 |
| GO:0034707 | 19 | 6 | 0.017011 | 0.103922 | chloride channel complex | 0.315789 |
| GO:0006091 | 16 | 5 | 0.024409 | 0.136563 | generation of precursor metabolites and energy | 0.3125 |
| GO:0006635 | 16 | 5 | 0.024409 | 0.136563 | fatty acid beta-oxidation | 0.3125 |
| GO:0006917 | 16 | 5 | 0.024409 | 0.136563 | induction of apoptosis | 0.3125 |
| GO:0019752 | 16 | 5 | 0.024409 | 0.136563 | carboxylic acid metabolic process | 0.3125 |
| GO:0043027 | 16 | 5 | 0.024409 | 0.136563 | cysteine-type endopeptidase inhibitor activity involved in apoptotic process | 0.3125 |
| GO:0017042 | 29 | 9 | 0.007824 | 0.063802 | glycosylceramidase activity | 0.310345 |
| GO:0006465 | 13 | 4 | 0.035245 | 0.179259 | signal peptide processing | 0.307692 |
| GO:0006829 | 13 | 4 | 0.035245 | 0.179259 | zinc ion transport | 0.307692 |
| GO:0008022 | 23 | 7 | 0.015896 | 0.097953 | protein C-terminus binding | 0.304348 |
| GO:0004175 | 53 | 16 | 0.001499 | 0.016255 | endopeptidase activity | 0.301887 |
| GO:0004383 | 20 | 6 | 0.022828 | 0.130813 | guanylate cyclase activity | 0.3 |
| GO:0006182 | 20 | 6 | 0.022828 | 0.130813 | cGMP biosynthetic process | 0.3 |
| GO:0009117 | 20 | 6 | 0.022828 | 0.130813 | nucleotide metabolic process | 0.3 |
| GO:0035085 | 20 | 6 | 0.022828 | 0.130813 | cilium axoneme | 0.3 |
| GO:0005576 | 1144 | 342 | 3.95E-40 | 2.33E-38 | extracellular region | 0.298951 |
| GO:0004983 | 27 | 8 | 0.014498 | 0.089742 | neuropeptide Y receptor activity | 0.296296 |
| GO:0008146 | 34 | 10 | 0.009178 | 0.072536 | sulfotransferase activity | 0.294118 |
| GO:0008137 | 69 | 20 | 0.000956 | 0.011614 | NADH dehydrogenase (ubiquinone) activity | 0.289855 |
| GO:0008483 | 38 | 11 | 0.0082 | 0.066539 | transaminase activity | 0.289474 |
| GO:0007218 | 159 | 46 | 2.56E-06 | 8.77E-05 | neuropeptide signaling pathway | 0.289308 |
| GO:0016627 | 45 | 13 | 0.005178 | 0.046064 | oxidoreductase activity. acting on the CH-CH group of donors | 0.288889 |
| GO:0008199 | 28 | 8 | 0.018565 | 0.112445 | ferric iron binding | 0.285714 |
| GO:0005254 | 21 | 6 | 0.029883 | 0.157643 | chloride channel activity | 0.285714 |
| GO:0006665 | 21 | 6 | 0.029883 | 0.157643 | sphingolipid metabolic process | 0.285714 |
| GO:0050909 | 14 | 4 | 0.048173 | 0.231069 | sensory perception of taste | 0.285714 |
| GO:0051287 | 118 | 33 | 0.000104 | 0.001939 | NAD binding | 0.279661 |
| GO:0016884 | 18 | 5 | 0.043353 | 0.215853 | carbon-nitrogen ligase activity. with glutamine as amido-N-donor | 0.277778 |
| GO:0030308 | 29 | 8 | 0.023398 | 0.133003 | negative regulation of cell growth | 0.275862 |
| GO:0006814 | 99 | 27 | 0.000567 | 0.007268 | sodium ion transport | 0.272727 |
| GO:0006730 | 44 | 12 | 0.011065 | 0.077892 | one-carbon metabolic process | 0.272727 |
| GO:0006108 | 22 | 6 | 0.038267 | 0.192333 | malate metabolic process | 0.272727 |
| GO:0006826 | 22 | 6 | 0.038267 | 0.192333 | iron ion transport | 0.272727 |
| GO:0010466 | 118 | 32 | 0.000242 | 0.004016 | negative regulation of peptidase activity | 0.271186 |
| GO:0030414 | 118 | 32 | 0.000242 | 0.004016 | peptidase inhibitor activity | 0.271186 |
| GO:0070469 | 126 | 34 | 0.000184 | 0.00313 | respiratory chain | 0.269841 |
| GO:0004364 | 63 | 17 | 0.004583 | 0.04146 | glutathione transferase activity | 0.269841 |
| GO:0004568 | 26 | 7 | 0.033442 | 0.17512 | chitinase activity | 0.269231 |
| GO:0004252 | 275 | 74 | 1.28E-07 | 5.23E-06 | serine-type endopeptidase activity | 0.269091 |
| GO:0004180 | 87 | 23 | 0.001973 | 0.021076 | carboxypeptidase activity | 0.264368 |
| GO:0007586 | 69 | 18 | 0.005662 | 0.050154 | digestion | 0.26087 |
| GO:0048037 | 50 | 13 | 0.014 | 0.087026 | cofactor binding | 0.26 |
| GO:0005758 | 27 | 7 | 0.041419 | 0.206706 | mitochondrial intermembrane space | 0.259259 |
| GO:0007268 | 31 | 8 | 0.035624 | 0.180755 | synaptic transmission | 0.258065 |
| GO:0005764 | 149 | 38 | 0.000316 | 0.00505 | lysosome | 0.255034 |
| GO:0006950 | 169 | 43 | 0.000154 | 0.002691 | response to stress | 0.254438 |
| GO:0005549 | 170 | 43 | 0.000178 | 0.003057 | odorant binding | 0.252941 |
| GO:0004601 | 60 | 15 | 0.014521 | 0.089742 | peroxidase activity | 0.25 |
| GO:0019137 | 36 | 9 | 0.03682 | 0.186382 | thioglucosidase activity | 0.25 |
| GO:0004129 | 53 | 13 | 0.023193 | 0.132193 | cytochrome-c oxidase activity | 0.245283 |
| GO:0022857 | 139 | 34 | 0.001316 | 0.014858 | transmembrane transporter activity | 0.244604 |
| GO:0016829 | 213 | 52 | 0.000126 | 0.002292 | lyase activity | 0.244131 |
| GO:0007166 | 41 | 10 | 0.037312 | 0.188419 | cell surface receptor signaling pathway | 0.243902 |
| GO:0000502 | 119 | 29 | 0.002746 | 0.026653 | proteasome complex | 0.243697 |
| GO:0004984 | 100 | 24 | 0.006622 | 0.054779 | olfactory receptor activity | 0.24 |
| GO:0050911 | 100 | 24 | 0.006622 | 0.054779 | detection of chemical stimulus involved in sensory perception of smell | 0.24 |
| GO:0005215 | 317 | 76 | 1.09E-05 | 0.000347 | transporter activity | 0.239748 |
| GO:0004527 | 63 | 15 | 0.022897 | 0.13086 | exonuclease activity | 0.238095 |
| GO:0022900 | 178 | 42 | 0.000999 | 0.012065 | electron transport chain | 0.235955 |
| GO:0031410 | 120 | 28 | 0.006088 | 0.051162 | cytoplasmic vesicle | 0.233333 |
| GO:0019843 | 69 | 16 | 0.025816 | 0.143679 | rRNA binding | 0.231884 |
| GO:0003899 | 66 | 15 | 0.034484 | 0.178139 | DNA-directed RNA polymerase activity | 0.227273 |
| GO:0016705 | 296 | 66 | 0.000363 | 0.005514 | oxidoreductase activity. acting on paired donors. with incorporation or reduction of molecular oxygen | 0.222973 |
| GO:0005975 | 368 | 82 | 8.84E-05 | 0.001724 | carbohydrate metabolic process | 0.222826 |
| GO:0003735 | 425 | 94 | 4.09E-05 | 0.001008 | structural constituent of ribosome | 0.221176 |
| GO:0016788 | 86 | 19 | 0.030042 | 0.15783 | hydrolase activity. acting on ester bonds | 0.22093 |
| GO:0043169 | 155 | 34 | 0.008654 | 0.069158 | cation binding | 0.219355 |
| GO:0008233 | 708 | 155 | 3.95E-07 | 1.56E-05 | peptidase activity | 0.218927 |
| GO:0020037 | 394 | 86 | 0.000133 | 0.002387 | heme binding | 0.218274 |
| GO:0004867 | 138 | 30 | 0.013875 | 0.086508 | serine-type endopeptidase inhibitor activity | 0.217391 |
| GO:0008610 | 202 | 43 | 0.007075 | 0.058302 | lipid biosynthetic process | 0.212871 |
| GO:0004497 | 368 | 78 | 0.000649 | 0.008122 | monooxygenase activity | 0.211957 |
| GO:0007608 | 118 | 25 | 0.028559 | 0.151415 | sensory perception of smell | 0.211864 |
| GO:0006633 | 137 | 29 | 0.021488 | 0.127534 | fatty acid biosynthetic process | 0.211679 |
| GO:0050660 | 175 | 37 | 0.012263 | 0.077892 | flavin adenine dinucleotide binding | 0.211429 |
| GO:0008236 | 228 | 48 | 0.00613 | 0.051306 | serine-type peptidase activity | 0.210526 |
| GO:0030170 | 105 | 22 | 0.040087 | 0.200531 | pyridoxal phosphate binding | 0.209524 |
| GO:0030529 | 565 | 115 | 0.000277 | 0.004468 | ribonucleoprotein complex | 0.20354 |
| GO:0004930 | 182 | 37 | 0.022355 | 0.129502 | G-protein coupled receptor activity | 0.203297 |
| GO:0016757 | 296 | 60 | 0.006487 | 0.054081 | transferase activity. transferring glycosyl groups | 0.202703 |
| GO:0004553 | 144 | 29 | 0.039883 | 0.199977 | hydrolase activity. hydrolyzing O-glycosyl compounds | 0.201389 |
| GO:0006508 | 706 | 140 | 0.000238 | 0.004016 | proteolysis | 0.1983 |
| GO:0005840 | 500 | 99 | 0.001726 | 0.018534 | ribosome | 0.198 |
| GO:0016491 | 1396 | 270 | 3.65E-06 | 0.000123 | oxidoreductase activity | 0.19341 |
| GO:0005506 | 442 | 85 | 0.007395 | 0.060703 | iron ion binding | 0.192308 |
| GO:0009055 | 437 | 81 | 0.020807 | 0.125667 | electron carrier activity | 0.185355 |
| GO:0055114 | 1506 | 278 | 9.72E-05 | 0.00188 | oxidation-reduction process | 0.184595 |
| GO:0006412 | 657 | 117 | 0.023969 | 0.13589 | translation | 0.178082 |
| GO:0003824 | 1474 | 258 | 0.003938 | 0.036091 | catalytic activity | 0.175034 |
| GO:0008152 | 1362 | 238 | 0.005894 | 0.05115 | metabolic process | 0.174743 |
| GO:0004872 | 810 | 141 | 0.030066 | 0.15783 | receptor activity | 0.174074 |
